# Stereoselective synthesis of protected l- and d-dideoxysugars and analogues *via* Prins cyclisations†
†Electronic supplementary information (ESI) available. See DOI: 10.1039/c5sc04144a


**DOI:** 10.1039/c5sc04144a

**Published:** 2016-01-11

**Authors:** Ryan J. Beattie, Thomas W. Hornsby, Gemma Craig, M. Carmen Galan, Christine L. Willis

**Affiliations:** a School of Chemistry , University of Bristol , Cantock's Close , Bristol BS8 1TS , UK . Email: m.c.galan@bristol.ac.uk ; Email: chris.willis@bristol.ac.uk

## Abstract

A *de novo* approach for the rapid construction of orthogonally protected l- and d-deoxysugars and analogues is described. A novel and robust silicon-acetal undergoes Prins cyclisations with a series of homoallylic alcohols in high yield and excellent stereocontrol. Modified Tamao–Fleming oxidation of the resulting silyltetrahydropyrans gives direct access to deoxyglycoside analogues and the approach was showcased in the synthesis of protected l-oliose, a component of the anticancer agent aclacinomycin A.

## Abstract 2

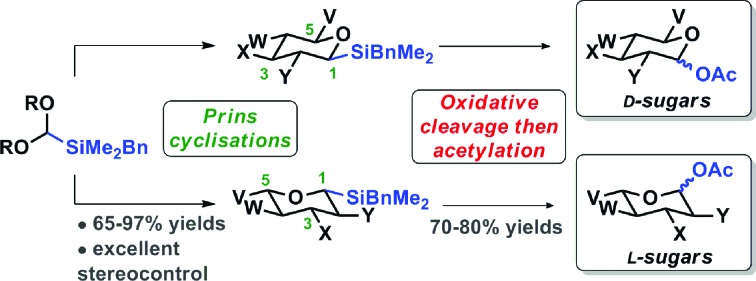
Cyclisation of a silicon acetal with homoallylic alcohols to generate silyltetrahydropyrans and subsequent oxidation gives rapid access to deoxyglycoside analogues.

## Introduction

Deoxyglycosides are important components of a wide variety of natural products isolated from plants, fungi and bacteria including compounds exhibiting anticancer and antibiotic activities. Some have proved effective for use in the clinic (*e.g.* the antibiotic vancomycin and the anthracycline antibiotic altromycin B) or as lead compounds to pharmaceuticals.^1^ In addition, deoxyglycans are also prevalent in bacterial membrane glycoproteins, thus being a viable target for drug discovery and vaccine development.^2^

The ability to fully understand and exploit the glycobiology of rare deoxysugars and analogues is hindered by the challenges of isolating pure materials in reasonable quantities from natural sources.^1^ In addition, synthetic approaches from naturally-abundant carbohydrates often require lengthy synthetic routes which make rare sugars very expensive.^3^ An alternative and potentially more versatile approach is the *de novo* asymmetric synthesis of deoxy sugars.^4^,^5^ An ideal synthetic strategy would be efficient, robust and readily adapted for the construction of a series of deoxysugars and derivatives. To this end, we have developed a new approach for the enantioselective synthesis of differentially-protected l- and d-deoxyglycosides and analogues *via* Prins cyclisations and its utility exemplified by the synthesis of 2,4- and 2,6-dideoxyglycosides including protected l-oliose.

**Scheme 1:**
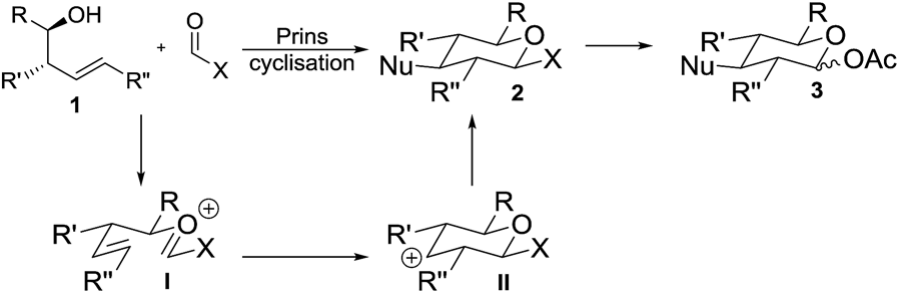
Proposed synthetic approach to deoxysugars.

Prins cyclisations involve acid-mediated reactions of homoallylic alcohols **1** (or derivatives thereof) to form an oxycarbenium ion **I** which cyclises *via* carbocation **II** and is subsequently trapped by a nucleophile, giving tetrahydropyrans **2** with excellent stereocontrol (Scheme 1).^6^ Reddy, Yadav and co-workers have used sugar derivatives as substrates in Prins cyclisations.^7^ Success of our proposed approach to deoxyglycosides relied upon use of a suitable electrophile bearing a hydroxyl surrogate (X in Scheme 1) which would need to be both stable to the acidic conditions required for the cyclisation and would readily be converted to a suitable functional group (*e.g.* acetate **3**) for use in glycosylation reactions. An orthoformate was considered as the electrophile to directly introduce a 1-*O*-alkyl side-chain, but these have rarely been used in Prins cyclisations and are limited to substrates in which the reaction proceeds *via* a tertiary carbocation.^8^,^9^

## Results and discussion

**Scheme 2:**
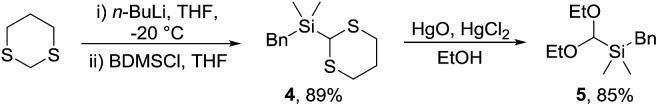
Synthesis of acetal **5**.

A trialkylsilane was considered a suitable hydroxyl surrogate as, following cyclisation, a Tamao–Fleming oxidation would lead to the required acetal. Whilst dimethylphenylsilanes have been widely used,^10^ Hosomi and co-workers reported the benzyldimethylsilyl group (BDMS) as an attractive alternative that is readily oxidised to alcohols.^11^ An important criterion for our synthetic strategy was that the electrophile should be stable and readily prepared on a synthetically valuable scale. Thus, novel silyl acetal **5** was prepared in two steps and 76% overall yield *via* treatment of 2-lithio-1,3-dithiane^12^ with benzyldimethylsilyl chloride (BDMSCl) to give dithiane **4** followed by mercuric-mediated deprotection in ethanol (Scheme 2).^13^ The reaction was conducted on a multigram scale and the acetal is stable with no apparent decomposition after 6 months on the bench.

**Scheme 3:**
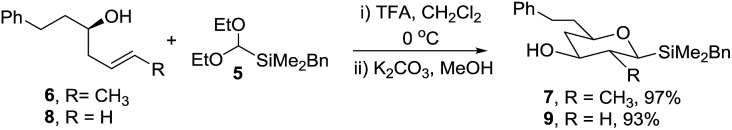
Cyclisation of homoallylic alcohols **6** and **8**.

Initially, the key Prins cyclisation was optimised using the known (*R*)-homoallylic alcohol **6** prepared from dihydrocinnamaldehyde *via* a Nokami crotyl transfer reaction.^14^ Several methods have been reported for the introduction of oxygen nucleophiles,^15^ and in this case treatment of alcohol **6** and acetal **5** with trifluoroacetic acid (TFA) at 0 °C,^16^ then hydrolysis of the resultant ester gave alcohol **7** in 97% yield (Scheme 3). A single diastereomer was isolated in which all four substituents were equatorial.

Our ultimate targets, 2,6- and 2,4-dideoxysugar analogues, lack a substituent at C-2 and their synthesis requires a substrate with a terminal alkene. Hence (*R*)-homoallylic alcohol **8** (prepared *via* a Brown allylation^17^) was treated with acetal **5** under the optimised reaction conditions to give alcohol **9** in 93% yield. It is known that the mechanism of Prins cyclisations is not simple and, depending on the nature of the substrate and reaction conditions, competing processes may occur.^18^ To ensure that there was no loss of stereochemical integrity during the cyclisation, the enantiopurity (97.5 : 2.5 e.r.) of **9** was confirmed by chiral SFC.

**Scheme 4:**
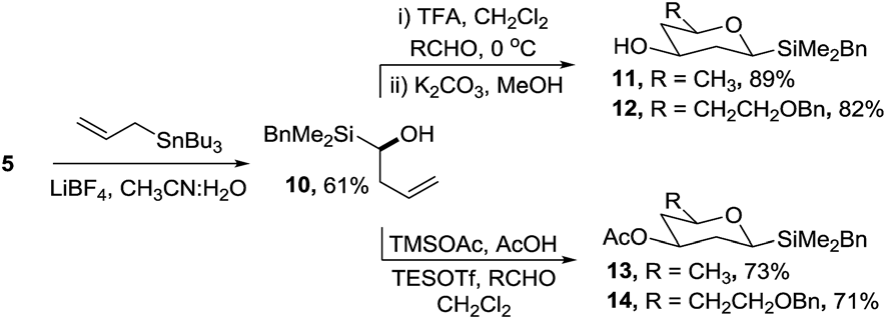
Alternative cyclisation strategy to silyltetrahydropyrans.

An alternative synthetic approach to the silyltetrahydropyrans was to incorporate the silyl moiety into the alkene coupling partner which on reaction with an aldehyde would enable the facile introduction of a range of side-chains at C-5 of the target deoxysugars (Scheme 4). Several methods were investigated for the synthesis of α-silyl-homoallylic alcohol **10***via* acid-mediated allylation of acetal **5** (*e.g.* in the presence of InCl_3_, AgNO_3_, SnCl_2_) but none of the required product was isolated. In contrast, when silyl acetal **5** was treated with allyltributylstannane and LiBF_4_ in wet acetonitrile, alcohol **10** was isolated in 61% yield.^19^ The TFA mediated reaction of **10** with either acetaldehyde or 3-benzyloxypropanal followed by hydrolysis of the resultant ester gave silyltetrahydropyrans **11** and **12** in 89% and 82% yields, respectively, from acetal **5**. By varying the reaction conditions the analogous acetates **13** and **14** were readily prepared. Further studies are ongoing to investigate the enantioselective allylation of acetal **5**.

**Scheme 5:**
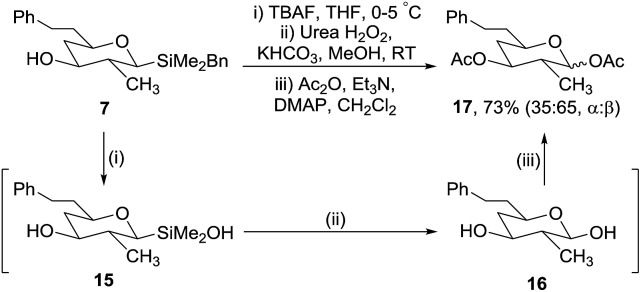
Oxidation of silane **7**.

The second stage of our synthetic strategy required oxidation of the benzyldimethylsilyl group; Trost and Donohoe have reported the use of tetrabutylammonium fluoride (TBAF) followed by hydrogen peroxide for similar transformations.^20^ Following detailed investigations we established suitable conditions for the successful oxidation of silane **7** (Scheme 5). It was evident that two steps are involved. First addition of TBAF converted silane **7** to silanol **15** which could be isolated and characterised.^21^

However, it was not necessary to isolate **15** as it was converted *in situ* to hemiacetal **16***via* a urea hydrogen peroxide oxidation and then directly acetylated to give **17** in 73% yield from silane **7**. It proved vital to keep the temperature of the fluoride activation step in the range 0–5 °C, as at higher temperatures disiloxanes were formed from the condensation of two silanols, which were only slowly oxidised under the reaction conditions.^22^

**Scheme 6:**
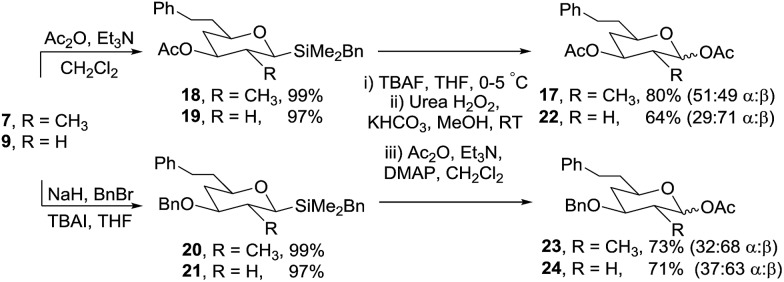
Oxidation of acetoxy and benzyloxy derivatives.

To confirm that the oxidation/acetylation protocol was compatible with different protecting groups commonly used in carbohydrate chemistry, the secondary alcohols in **7** and **9** were converted to acetates (**18** and **19**) and benzyl ethers (**20** and **21**) in high yields using standard reaction conditions (Scheme 6). Oxidation of each silane gave the corresponding anomeric acetates (**17**, **22–24**) in 64–80% isolated yield.

**Scheme 7:**
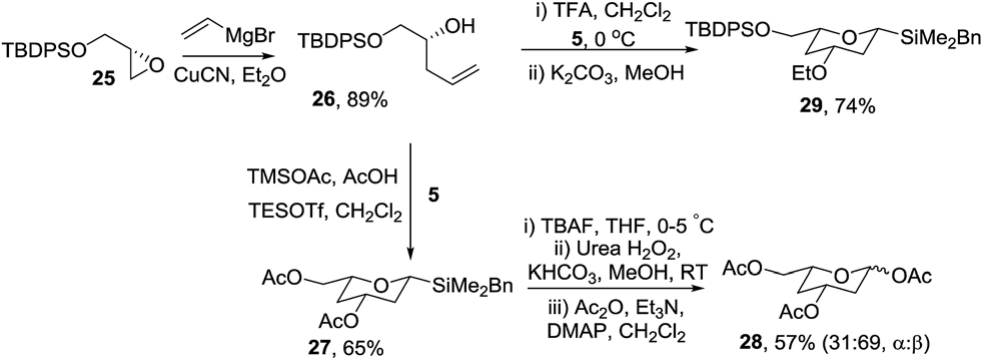
Preparation of 2,4-dideoxysugar **28**.

Next the optimised cyclisation/oxidation/acetylation strategy was applied to the preparation of protected 2,4-dideoxyglycosides (Scheme 7). Homoallylic alcohol **26** was prepared in 88% overall yield from (*S*)-glycidol *via* protection of the alcohol as silyl ether **25** and ring opening of the oxirane with vinylmagnesium bromide and CuCN. Treatment of **26** with silyl acetal **5** and TESOTf in acetic acid^23^ gave silyltetrahydropyran **27** in 65% yield which was readily converted to triacetate **28** as a 1 : 2 mixture of anomers using the oxidation/acetylation protocol. Interestingly, treatment of the mixture of homoallylic alcohol **26** and acetal **5** with TFA, our standard cyclisation conditions, gave none of the expected product, instead the analogous ethyl ether **29** was isolated.^24^ Some deoxysugars indeed have ethers at C-3, for example, d-oleandrose is a component of the highly potent and selective anticancer agent apoptolidin^25^ and l-cymarose, found in the DNA-helicase inhibitor, heliquinomycin^26^ and so this unexpected result has potential significant synthetic value.

**Scheme 8:**
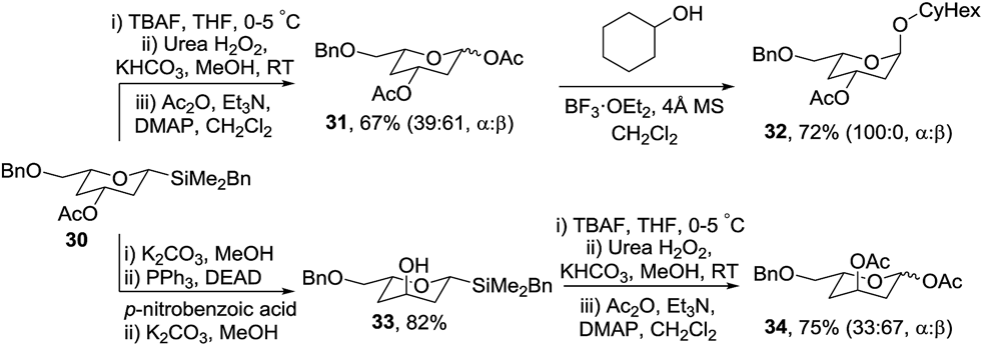
Synthesis of protected 2,4-dideoxyglycosides **31** and **34**.

To access orthogonally protected 2,4-dideoxyglycosides, benzyl-protected tetrahydropyran **30** was prepared *via* a similar protection/vinyl addition/cyclisation strategy from (*S*)-glycidol benzyl ether in 71% overall yield (Scheme 8). Oxidation of **30** gave diacetate **31** which subsequently was used to glycosylate cyclohexanol in the presence of BF_3_·OEt_2_ giving **32** as exclusively the α-anomer in 72% yield. The synthetic approach was extended to 2,4-dideoxyglycosides with an axial C-3 oxygenated substituent *via* hydrolysis of acetate **30** and Mitsunobu inversion to give **33** which was oxidised to protected glycoside **34** in 75% yield.

**Fig. 1:**
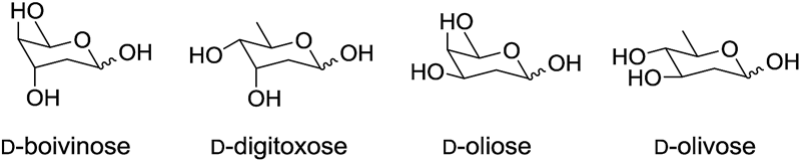
Examples of 2,6-dideoxyhexoses.

Next we turned out attention to the synthesis of protected 2,6-dideoxysugars as all diastereoisomers of d- and l-2,6-dideoxyhexoses have been found in biologically active natural products *e.g.*d-olivose is a component of angucylcline antibiotic landomycin A^27^ whilst d-digitoxose is present in the steroidal glycoside digitoxin (Fig. 1).

**Scheme 9:**
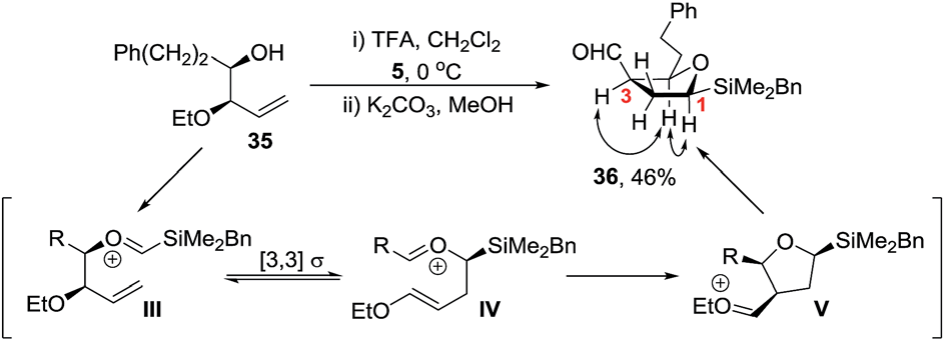
Cyclisation to tetrahydrofuran **36** and nOe correlations.

Initial studies revealed that whilst homoallylic alcohol **35** was readily prepared *via* Brown allylation of allyl ethyl ether,^28^ reaction of **35** with silyl acetal **5** under our standard TFA conditions gave the 5-membered ring aldehyde **36** in 46% yield (Scheme 9). Aldehyde **36** is likely to be formed *via* a Prins-pinacol reaction^29^ involving oxonia-Cope rearrangement of oxycarbenium ion **III** to enol ether **IV** followed by cyclisation to tetrahydrofuran **V** and finally *O*-alkyl cleavage to generate the carbonyl group.^30^ Hence to favour formation of a tetrahydropyran over a tetrahydrofuran we reasoned that an electron withdrawing group rather than an ether was required and a carbamate protecting group was selected.^31^

Thus an asymmetric synthesis of homoallylic alcohol **40** was required which could be readily adapted for both the l- or d-protected 2,6-dideoxysugars since, for example, l-oliose is a component of aclarubicin, clinically used for the treatment of acute leukaemias,^32^ whilst d-oliose is present in the antitumour drugs mithramycin and chromocyclomycin,^33^ as well as the HIV-inhibitor durhamycin A.^34^

**Scheme 10:**
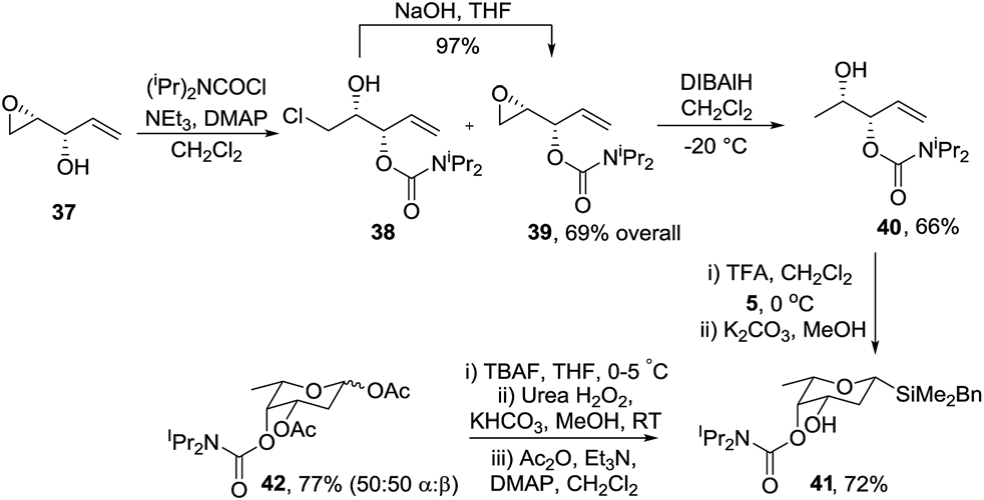
Enantioselective synthesis of protected l-oliose **42**.

**Scheme 11:**
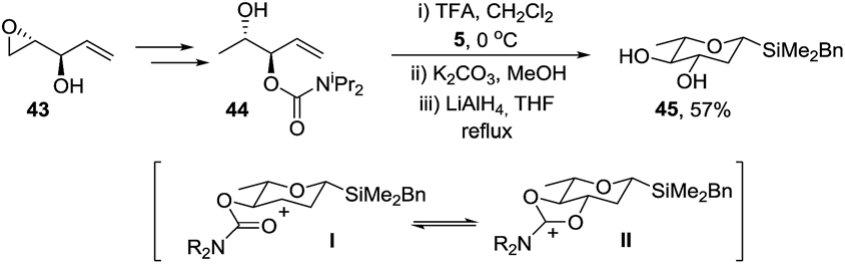
Enantioselective synthesis of l-olivose precursor **45**.

Singh and Guiry reported that Sharpless asymmetric epoxidation (SAE) of divinylcarbinol, followed by Mitsunobu inversion of the resulting alcohol gives epoxide **37** (Scheme 10).^35^ Importantly, choice of (–)-DIPT or (+)-DIPT in the SAE step allows access to the d- and l-series, respectively. Protection of alcohol **37** with *N*,*N*-diisopropylcarbamoyl chloride gave a mixture of chlorohydrin **38** and the required epoxide **39**. Chlorohydrin **38** was readily converted to **39** by treatment with NaOH in THF at room temperature within a matter of minutes. Reductive ring opening of the oxirane with DIBALH gave mono-protected *syn* allylic diol **40** which cyclised with acetal **5** to give the required tetrahydropyran **41** with an equatorial C-3 alcohol and the axial C-4 protected hydroxyl. Finally oxidation of silane **41** gave protected l-oliose **42** in 77% yield as a 1 : 1 mixture of anomers. The methodology could be extended to the synthesis of l-olivose *via* protected alcohol **44** which was prepared from epoxide **43** using the same conditions as for assembly of the diastereomer **40** (Scheme 11). Interestingly, reaction of **44** with acetal **5** under the standard conditions gave a mixture of products due to migration of the carbamoyl group but on reduction of the mixture with LiAlH_4_, diol **45** was isolated in 57% yield. It is possible that neighbouring group participation by the carbamoyl group traps the intermediate carbocation **I** in the Prins cyclisation giving **II** and resulting in migration of the carbomyl group but this has not been proven.

## Conclusions

In conclusion, a *de novo* approach for the rapid construction of a series of orthogonally protected l- and d-dideoxyglycosides and analogues is described from simple starting materials. A stable acetal **5** was prepared in two high yielding steps and used in a series of acid-mediated Prins cyclisations with different homoallylic alcohols to give the corresponding tetrahydropyrans in good yield and excellent diastereoselectivity. These reactions are readily performed on gram scales. A modified Tamao–Fleming oxidation/acetylation protocol gave the target 2,4-dideoxysugars with an acetyl group at the anomeric position. Extending the utility of the new methodology to the synthesis of 2,6-dideoxysugars revealed the importance of the choice of protecting group to avoid formation of tetrahydrofuranals. The enantioselective synthesis of protected l-oliose is described using *N*,*N*-diisopropylcarbamoyl as a protecting group. Silane **41** has potential value for the synthesis of other 2,6-dideoxyhexoses for example methylation of the free hydroxyl group will lead to protected l-diginose while Mitsunobu inversion will give l-boivinose derivatives and subsequent methylation to l-sarmentose and these investigations are ongoing in our laboratories.

## Supplementary Material

Supplementary information
