# Supplementary material for: Stereoselective synthesis of protected l- and d-dideoxysugars and analogues via Prins cyclisations
Source: Chem Sci. 2016 Jan 11;7(4):2743–7. doi: 10.1039/c5sc04144a (PMC5477037; doi:10.1039/c5sc04144a)
Supplement: Supplementary file 1 [file SC-007-C5SC04144A-s001.pdf]

Electronic Supplementary Material (ESI) for Chemical Science.
This journal is © The Royal Society of Chemistry 2016

Stereoselective Synthesis of Protected L- and D-Dideoxysugars and Analogues *via* Prins Cyclisations

Ryan J. Beattie, Thomas W. Hornsby, Gemma Craig, M. Carmen Galan and Christine L. Willis**

Supporting Information

1. General Information, *2*
2. Preparation of Compounds, *3*
3. Chiral SFC Data, *36*
4. ^{1}H and ^{13}C NMR Spectra, *37*
5. References, *75*

General Procedures

Unless otherwise stated, all reagents were purchased from commercial suppliers and used without further purification. Anhydrous solvents were used which were dried using the Anhydrous Engineering Ltd. double alumina and alumina-copper catalysed drying columns. All moisture or air sensitive reactions were carried out in flame dried glassware under a positive pressure of N2 using standard syringe/septa techniques. Flash column chromatography was performed on silica gel (Merck Kieselgel 60, 230-400 mesh). Thin layer chromatography was carried out on Polygram 0.2 mm silica gel TLC plates visualising with 254 nm UV light and developing with either a $KMnO_4$, phosphomolybdic aicd or Vanillin dip, where appropriate.

Optical rotations were determined with the sodium D line (λ= 589 nm) using a Perkin Elmer 241 MC polarimeter. $[\alpha]_D^{22}$ values are quoted in units 10^{-1} deg cm^2 g^{-1}. Infrared (IR) spectroscopy was recorded on a Perkin Elmer Spectrum 100 FT-IR spectrometer with an ATR diamond cell irradiating between 4000 cm^{-1} and 600 cm^{-1}. Melting points were determined using an electrothermal melting point apparatus and are uncorrected. Electron impact (EI) and chemical ionisation (CI) mass spectra were recorded on a VG Analytical Autospec mass spectrometer. Methane was the ionization gas used for CI. Electrospray ionisation (ESI) mass spectra were recorded on a Micromass LCT mass spectrometer or a VG Quattro mass spectrometer.

NMR spectra were recorded using either a Varian 400 MHz or JEOL ecp 400 MHz spectrometer. Chemical shifts (δ_H) are quoted in parts per million (ppm), *J* values are given in Hz and referenced to the appropriate residual solvent peak. Data reported as follows: chemical shift, integration, multiplicity (s = singlet, br s = broad singlet, d = doublet, t = triplet, q= quartet, qi = quintet, sx = sextet, hept = heptet, m = multiplet, dd = doublet of doublet, etc.), coupling constants, assignment. Chemical shifts (δ_C) are quoted in parts per million (ppm), referenced to the appropriate residual solvent peak. DEPT[135], COSY and HMQC were used for all new compounds in assigning NMR spectra. Chiral SFC was performed using Diacel Chiralpak IA, IB and IC columns (4.6 × 250 mm × 5 µm) or a Whelk O-1 column (4.6 × 250 mm × 5 µm) on a WatersTharSFC system and monitored by DAD (Diode Array Detector).

Preparation of compounds

Benzyl(1,3-dithiane)dimethylsilane 4

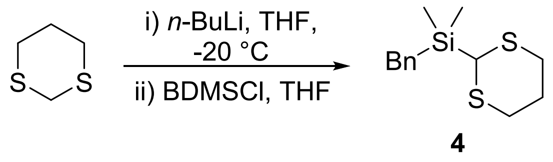

n-Butyllithium (14 mL, 1.57 M solution in hexanes, 22.0 mmol) was added dropwise to 1,3-dithiane (2.2 g, 18.3 mmol) in dry THF (60 mL) at -15 °C under N_2. This was stirred for 6 h, warming to RT slowly. The solution was then added dropwise *via* cannula to a solution of benzyl(dimethyl)chlorosilane (3.7 mL, 20.2 mmol) in dry THF (30 mL) at 0 °C and the reaction allowed to warm to RT and stirred for 14 h. A saturated solution of ammonium chloride (25 mL) was added, the organic phase separated and aqueous layer extracted with EtOAc (3 x 20 mL). The combined organic phases were dried ($MgSO_4$) and concentrated *in vacuo* to yield a dark brown oil. This was purified by column chromatography (Pet: EtOAc, 99:1) to afford a brown oil. Further purification by bulb to bulb distillation gave thioacetal **4** as a colourless oil (4.4 g, 89%); bp 235 °C at 8.0 mbar; ν_{max} (neat)/cm^{-1} 3059 (ArCH), 2953 (CH), 1599 (ArC=C); δ_H (400 MHz; $CDCl_3$) 0.14 (6H, s, 2 x Si*C*H_3), 1.99-2.18 (2H, m, CH_2), 2.27 (2H, s, CH_2Ph), 2.29 (2H, dt, *J* 14.3, 3.2, SCH_2), 2.78 (2H, td, *J* 14.3, 2.9, SCH_2), 3.72 (1H, s, SC*H*), 7.08-7.15 (3H, m, ArH), 7.23-7.25 (2H, m, ArH); δ_C (100 MHz; $CDCl_3$) -5.2 (2 x Si*C*H_3), 23.1 (*C*H_2Ph), 26.2 (*C*H_2), 31.0 (2 x S*C*H_2), 32.9 (*C*H), 124.3 (C-Ar), 128.1 (C-Ar), 128.3 (C-Ar), 138.9 (C-Ar); *m/z* (ESI) 291.0679 (MNa^+, 100%, $C_{13}H_{20}NaS_2Si$ requires 291.0668).

Benzyl(diethoxymethyl)dimethylsilane 5

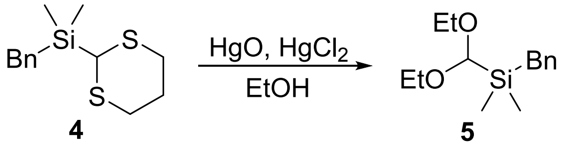

2-BDMS-1,3-dithiane **4** (6.35 g, 23.6 mmol) in dry ethanol (50 mL) was added to a two-neck round bottomed-flask equipped with a condenser and placed under N_2. Mercury (II) chloride

(19.2 g, 70.8 mmol) and mercury (II) oxide (1.63 g, 47.2 mmol) were added and the resulting suspension was stirred vigorously at reflux for 3 h. The reaction mixture was filtered through Celite®, washing with Et_2O (3 x 10 mL) and concentrated *in vacuo* to yield a white oily residue. Purification by column chromatography (Pentane: Et_2O, 98:2) gave the silyl acetal **5** as a colourless oil (5.05 g, 85%); ν_{max} (neat)/cm^{-1} 3060 (ArCH), 2958 (CH), 1601 (C=C), 1056 (C-O); δ_H (400 MHz; $CDCl_3$) 0.06 (6H, s, 2 x SiCH_3), 1.22 (6H, t, *J* 7.1, CH_3), 2.20 (2H, s, CH_2Ph), 3.48 (2H, q, *J* 7.1, CH_2), 3.77 (2H, q, *J* 7.1, CH_2), 4.38 (1H, s, C*H*), 7.04-7.10 (3H, m, ArH), 7.21-7.25 (2H, m, ArH); δ_C (100 MHz; $CDCl_3$) -5.5 (SiCH_3), 15.6 (2 x CH_3), 23.3 (CH_2Ph), 65.7 (OCH_2), 106.5 (*C*H), 124.1 (C-Ar), 128.2 (C-Ar), 128.3 (C-Ar), 139.4 (C-Ar); *m/z* (ESI) 275.1427 (MNa^+, 100%, $C_{14}H_{24}O_2NaSi$ requires 275.1438).

(5E,3R)-1-Phenylhept-5-en-3-ol 6[1]

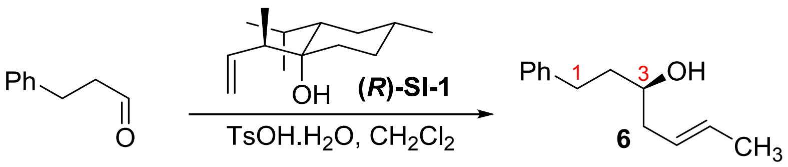

Para-toluenesulfonic acid monohydrate (0.21 g, 1.1 mmol) was added to a solution of dihydrocinnamaldehyde (1.46 mL, 11.1 mmol) and alcohol **(*R*)-SI-1**[1] (2.32 g, 11.1 mmol) in dry CH_2Cl_2 (15 mL) under N_2. The mixture was stirred for 24 h at RT, then aqueous saturated sodium hydrogen carbonate (35 mL) was added. Triethylamine was added until the pH >7 and the mixture was stirred for 20 minutes. The resulting biphasic solution was separated and the aqueous layer extracted with CH_2Cl_2 (4 x 30 mL). The combined organic phases were washed with aqueous saturated $NaHCO_3$ (30 mL), dried ($MgSO_4$) and concentrated *in vacuo*. Purification using column chromatography (Pet: EtOAc, 95:5) gave alcohol **6** as a yellow oil (1.45 g, 69%); $[\alpha]^{22}_D$ + 10.0 (*c* 1.05, $CHCl_3$), lit.[1] $[\alpha]^{25}_D$ + 14.0 (c 1.00, $CHCl_3$); ν_{max} (neat)/cm^{-1} 3383 (OH), 3062 (ArCH), 3026 (C=CH), 2917 (CH), 1603 (ArC=C); δ_H (400 MHz; $CDCl_3$) 1.70 (3H, dd, *J* 6.2, 1.0, 7-H_3), 1.75-1.82 (2H, m, 2-H_2), 2.09 (1H, m, 4-*H*H), 2.25 (1H, m, 4-H*H*), 2.69 (1H, dt, *J* 13.9, 8.4, 1-*H*H), 2.82 (1H, dt, *J* 13.9, 7.7, 1-H*H*), 3.62 (1H, m, 3-H), 5.43 (1H, dqt, *J* 15.2, 6.2, 1.5, 6-H), 5.57 (1H, tq, *J* 15.2, 6.2, 1.0, 5-H), 7.18-7.23 (3H, m, ArH), 7.28-7.32 (2H, m, ArH); δ_C (100 MHz; $CDCl_3$) 18.3 (C-7), 32.3 (C-2), 38.6 (C-1), 41.0 (C-4), 70.5 (C-3), 125.5 (C-Ar), 126.8 (C-6), 128.1 (C-Ar), 128.2 (C-Ar), 128.6 (C-5), 142.0 (ArC). Spectroscopic data were in accordance with the literature.[1]

(1*S*,2*R*,3*S*,5*R*)-1-(Benzyldimethylsilane)-2-methyl-3-hydroxy-5-(2'-phenylethyl)-tetrahydropyran 7

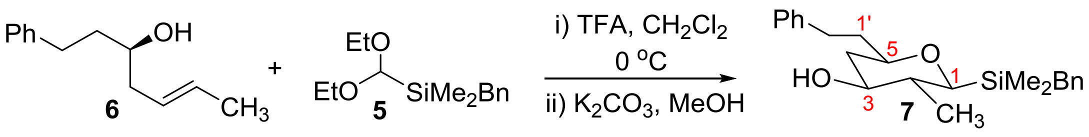

Trifluoroacetic acid (760 µl, 7.8 mmol) was added dropwise to a solution of alcohol **6** (99 mg, 0.52 mmol) and silyl acetal **5** (156 mg, 0.62 mmol) in dry CH_2Cl_2 (6 mL) at RT under N_2. The reaction was stirred for 6 h, then aqueous saturated $NaHCO_3$ (15 mL) was added carefully. The organic phase was separated and the aqueous phase extracted with CH_2Cl_2 (3 x 25 mL). The organic phases were combined, washed with aqueous saturated $NaHCO_3$ (10 mL) and then concentrated *in vacuo.* The resulting crude residue was redissolved in methanol (10 mL), to which K_2CO_3 (430 mg, 3.12 mmol) was added and stirred for 15 minutes. The methanol was removed under reduced pressure, water (10 mL) added and extracted with CH_2Cl_2 (3 x 20 mL). The combined organic phases were washed with brine (5 mL), dried ($MgSO_4$) and concentrated *in vacuo* to afford the crude residue. Which was purified by column chromatography (Pet: EtOAc, 85:5) to give alcohol **7** as a yellow oil (186 mg, 97%); $[\alpha]_D^{22}$ + 28.0 (*c* 1.01, $CHCl_3$); ν_{max} (neat)/cm^{-1} 3327 (OH), 3060 (ArCH), 3024 (ArCH), 2931 (CH), 1600 (ArC=C); δ_H (400 MHz; $CDCl_3$) 0.05 (3H, s, SiC*H₃*), 0.08 (3H, s, SiC*H₃*), 0.95 (3H, d, *J* 6.6, 2-C*H₃*), 1.29 (1H, app. q., *J* 11.3, 4-H_{ax}), 1.52 (1H, m, 2-H_{ax}), 1.73 (1H, m, 1'-*H*H), 1.87 (1H, m, 1'-H*H*), 1.94 (1H, ddd, *J* 11.3, 4.9, 2.0, 4-H_{eq}), 2.21 (1H, d, *J* 13.7, SiC*H*H), 2.31 (1H, d, *J* 13.7, SiCH*H*), 2.68 (1H, m, 2'-*H*H), 2.74 (1H, d, *J* 11.0, 1-H_{ax}), 2.81 (1H, m, 2'-H*H*), 3.23-3.29 (2H, m, 5-H and 3-H), 7.05-7.11 (3H, m, ArH), 7.17-7.31 (7H, m, ArH); δ_C (100 MHz; $CDCl_3$) -4.7 (Si*C*H₃), -3.6 (Si*C*H₃), 13.4 (2-*C*H₃), 23.9 (Si*C*H₂), 31.8 (C-2'), 38.0 (C-4), 41.2 (C-2), 41.5 (C-1'), 74.0 (C-1), 74.8 (C-3), 77.5 (C-5), 124.0 (C-Ar), 125.7 (C-Ar), 128.1 (C-Ar), 128.3 (C-Ar), 128.4 (C-Ar), 128.5 (C-Ar) 140.0 (C-Ar), 142.3 (C-Ar); *m/z* (ESI) 391.2051 (MNa^+, 100%, $C_{23}H_{32}O_2NaSi$ requires 391.2069).

(1*S*,3*S*,5*R*)-1-(Benzyldimethylsilane)-3-hydroxy-5-(2'-phenylethyl)-tetrahydropyran 9

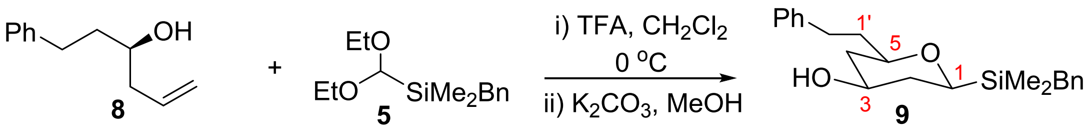

Trifluoroacetic acid (3.32 mL, 34.1 mmol) was added dropwise to a solution of alcohol **8** (200 mg, 1.14 mmol) and silyl acetal **5** (315 mg, 1.25 mmol) in dry CH_2Cl_2 (12 mL) at RT under N_2. This was stirred for 3 h at RT, then aqueous saturated $NaHCO_3$ (15 mL) was added carefully. The organic phase was separated and the aqueous phase extracted with CH_2Cl_2 (3 x 25 mL). The organic phases were combined, washed with aqueous saturated $NaHCO_3$ (10 mL) and then concentrated *in vacuo*. The resulting crude residue was redissolved in methanol (10 mL), to which K_2CO_3 (1.13 g, 8.19 mmol) was added and left to stir for 30 minutes. The methanol was removed under reduced pressure, water (25 mL) added and extracted with CH_2Cl_2 (3 x 30 mL). The combined organic phases were dried ($MgSO_4$) and concentrated *in vacuo* to afford the crude residue. Which was purified by column chromatography (Pet: EtOAc, 85:5) to give alcohol **9** as a yellow oil (374 mg, 93%); $[\alpha]_D^{22}$ + 34.0 (*c* 1.00, $CHCl_3$); ν_{max} (neat)/cm^{-1} 3323 (OH), 3062 (ArCH), 3021 (ArCH), 2934 (CH), 1601 (ArC=C); δ_H (400 MHz; $CDCl_3$) 0.02 (3H, s, SiCH_3), 0.05 (3H, s, SiCH_3), 1.20 (1H, app. q., *J* 11.0, 4-H_{ax}), 1.52 (1H, app. q, *J* 12.8, 2-H_{ax}), 1.51 (1H, br. s., O*H*), 1.71 (1H, m, 1'-*H*H), 1.79 (1H, m, 1'-H*H*), 1.88 (1H, m, 2-H_{eq}), 1.93 (1H, ddd, *J* 11.0, 5.0, 2.0, 4-H_{eq}), 2.17 (1H, d, *J* 13.6, SiC*H*H), 2.25 (1H, d, *J* 13.6, SiCH*H*), 2.71 (1H, m, 2'-*H*H), 2.81 (1H, m, 2'-H*H*), 3.04 (1H, dd, *J* 12.8, 2.0, 1-H), 3.23 (1H, tdd, *J* 11.0, 3.9, 2.0, 5-H), 3.71 (1H, br. ddt, *J* 12.8, 11.0, 5.0, 3-H), 7.04-7.11 (3H, m, ArH), 7.20-7.31 (7H, m, ArH); δ_C (100 MHz; $CDCl_3$) -6.0 (SiCH_3), -5.8 (SiCH_3), 23.0 (SiCH_2), 31.8 (C-2'), 36.4 (C-4), 38.0 (C-2), 41.8 (C-1'), 68.1 (C-1), 69.3 (C-3), 77.1 (C-5), 124.0 (C-Ar), 125.7 (C-Ar), 128.2 (C-Ar), 128.2 (C-Ar), 128.5 (C-Ar), 128.8 (C-Ar) 139.8 (C-Ar), 142.3 (C-Ar); *m/z* (ESI) 377.1907 (MNa^+, 100%, $C_{22}H_{30}O_2NaSi$ requires 377.1913).

1-(Benzyl(dimethyl)silyl)but-3-en-1-ol 10

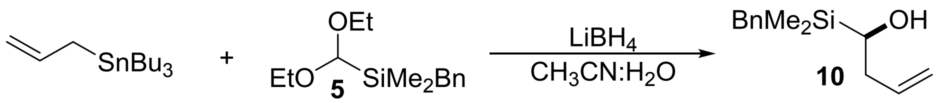

Lithium tetrafluoroborate (58 mg, 0.62 mmol) was added in one portion to silyl acetal **5** (142 mg, 0.56 mmol) and allyltributylstannane (350 µl, 1.13 mmol) in MeCN (2.8 ml) with H_2O (20 µl) at -15 °C. This was left to slowly warm to RT over 2 h. Saturated aqueous $NaHCO_3$ (5 ml) was then added and the organics were extracted with EtOAc (3 x 10 ml). The combined organic phases were then dried ($MgSO_4$) and concentrated *in vacuo*. Purification by column chromatography (Pet: Et_2O, 91:9) afforded the title compound **10** as a colorless oil (75 mg, 61%); ν_{max} (neat)/cm^{-1} 3319 (OH), 3061 (ArCH), 3032 (ArCH), 1601 (ArC=C); δ_H (400 MHz; $CDCl_3$) 0.01 (SiC*H3*), 0.06 (SiC*H3*), 2.15 (1H, m, SiC*H*HPh), 2.18 (1H, d, *J* 13.6, SiCH*H*Ph), 2.25 (1H, m, 2-*H*H), 2.34 (1H, m, 2-H*H*), 3.32 (1H, dd, *J* 11.2, 3.1, 1-H), 5.11–5.19 (2H, m, 4-H_2), 5.77 (1H, m, 3-H), 7.04- 7.26 (5H, m, Ar-H); δ_C (100 MHz; $CDCl_3$) -6.0 (Si*C*H3), -5.7 (Si*C*H3), 23.2 (Si*C*H2Ph), 38.0 (C-2), 62.3 (C-1), 118.1 (C-4), 124.1 (C-Ar), 128.2 (2 x C-Ar), 128.3 (2 x C-Ar), 135.8 (C-3), 139.6 (C-Ar); *m/z* (ESI) 243.1253 (MNa^+, 100%, $C_{13}H_{20}ONaSi$ requires 243.1283).

(1S*,3S*,5R*)-1-(Benzyl(dimethyl)silyl)-3-hydroxy-5-methyl-tetrahydropyran 11

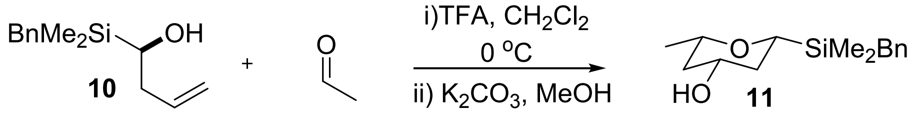

Trifluoroacetic acid (185 µl, 1.91 mmol) was added dropwise to a solution of alcohol **10** (21 mg, 0.10 mmol) and acetaldehyde (32 µl, 0.60 mmol) in dry CH_2Cl_2 (1 ml) at room temperature. This was stirred for 50 minutes at room temperature, then aqueous saturated $NaHCO_3$ (3 ml) and triethylamine was added until pH >7. The organic phase was separated and the aqueous phase extracted with CH_2Cl_2 (3 x 5 ml). The combined organic phase was then concentrated *in vacuo* and the resulting crude residue was redissolved in methanol (4 ml), to which K_2CO_3 (80 mg, 0.6 mmol) was added and left to stir for 30 minutes. The methanol was removed under reduced pressure, water (5 ml) added and extracted with CH_2Cl_2 (3 x 5 ml). The combined organic phases were dried ($MgSO_4$) and concentrated *in vacuo* to afford the crude residue. This was further

purified by column chromatography (Pet: EtOAc, 80:20) to afford alcohol **11** as a colorless oil (22 mg, 89%); ν_{max} (neat)/cm^{-1} 3321 (OH), 3063 (ArCH), 3021 (ArCH), 2934 (CH), 1600 (ArC=C); δ_H (400 MHz; $CDCl_3$) -0.04 (3H, s, SiC*H3*), 0.02 (3H, s, SiC*H3*), 1.14 (1H, app. q, *J* 11.0, 4-H_{ax}), 1.20 (3H, d, *J* 6.1, 1'-*H3*), 1.30 (1H, app. q, *J* 11.0, 2-H_{ax}), 1.58 (1H, br. s, O*H*), 1.76 (1H, ddd, *J* 11.0, 6.6, 2.0, 2-H_{eq}), 1.94 (1H, ddd, *J* 11.0, 6.6, 2.0, 4-H_{eq}), 2.12 (1H, d, *J* 13.5, SiC*H*HPh), 2.23 (1H, d, *J* 13.5, SiCH*H*Ph), 3.01 (1H, dd, *J* 11.0, 2.0, 1-H_{ax}), 3.33 (1H, app. sext. of d., *J* 6.6, 2.0, 5-H_{ax}), 3.71 (1H, ttd, *J* 11.0, 6.6, 2.0, 3-H_{ax}), 7.02 -7.10 (3H, m, Ar-H), 7.19- 7.23 (2H, m, Ar-H); δ_C (100 MHz; $CDCl_3$) -6.1 (Si*C*H_3), -5.9 (Si*C*H_3), 22.1 (C-1'), 23.0 (Si*C*H_2Ph), 36.1 (C-2), 43.5 (C-4), 68.0 (C-1), 69.3 (C-3), 74.4 (C-5), 124.0 (C-Ar), 128.1 (2 x C-Ar), 128.2 (2 x C-Ar), 139.9 (C-Ar); *m/z* (ESI) 287.1439 (MNa^+, 100%, $C_{15}H_{24}O_2NaSi$ requires 287.1438).

(1S*,3S*,5R*)-1-(Benzyl(dimethyl)silyl)-3-hydroxy-5-(2'-(benzyloxy)ethyl)-tetrahydropyran 12

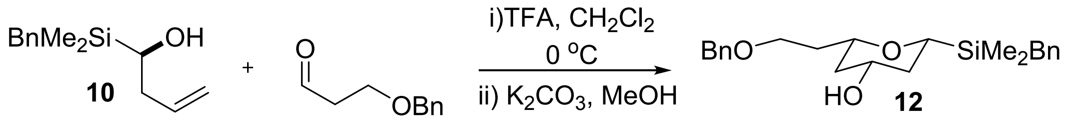

Trifluoroacetic acid (0.56 mL, 7.26 mmol) was added dropwise to a solution of alcohol **10** (80 mg, 0.36 mmol) and 3-benzyloxypropanal (120 mg, 0.73 mmol) in dry CH_2Cl_2 (5 ml) at RT. This was stirred for 50 minutes then aqueous saturated $NaHCO_3$ (3 ml) and triethylamine was added until pH >7. The organic phase was separated and the aqueous phase extracted with CH_2Cl_2 (3 x 10 mL). The combined organic phases were concentrated *in vacuo* and the resulting crude residue was redissolved in methanol (12 mL), to which K_2CO_3 (100 mg, 0.73 mmol) was added and left to stir for 30 minutes. The methanol was removed under reduced pressure, water (10 mL) added and extracted with CH_2Cl_2 (3 x 10 mL). The combined organic phases were dried ($MgSO_4$) and concentrated *in vacuo* to afford the crude residue which was purified by column chromatography (Pet: EtOAc, 90:10) to afford alcohol **12** as a colorless oil (114 mg, 82%); ν_{max} (neat)/cm^{-1} 3370 (OH), 3065 (ArCH), 3025 (ArCH), 2927 (CH), 1600 (ArC=C), 1028 (C-O); δ_H (400 MHz; $CDCl_3$) -6.0 (3H, s, SiC*H3*), 0.03 (3H, s, SiC*H3*), 1.19 (1H, app. q., *J* 12.0, 2-H_{ax}), 1.43 (1H, app. td, *J* 12.5, 10.5, 4-H_{ax}), 1.74-1.85 (3H, m, 1'-*H*$_2$ and 4-H_{eq}), 1.97 (1H, ddd, *J* 12.0, 4.5, 2.0, 2-H_{eq}), 2.12 (1H, d, *J* 13.5, SiC*H*H), 2.22 (1H, d, *J* 13.5, SiCH*H*), 3.11 (1H, dd, *J* 13.0, 2.0, 1-H), 3.43 (1H, dddd, J 10.5, 8.5, 4.5, 2.0, 5-H), 3.59-3.75 (3H, m, 2'-*H*$_2$ and 3-H), 4.54 (2H, s, C*H*$_2$Ph), 7.04 (2H, *J* 7.5, Ar-H), 7.09 (1H, t, *J* 7.5, Ar-H), 7.21 (2H, t, *J* 7.5, Ar-H) 7.30 (1H, m, ArH), 7.34-7.37 (4H, m,

ArH); δ_C (100 MHz; $CDCl_3$) -6.1 (SiCH_3), -5.8 (SiCH_3), 23.1 (SiCH_2), 36.4 (C-4), 36.7 (C-1'), 42.0 (C-2), 67.0 (C-2'), 68.0 (C-1), 69.3 (C-3), 73.1 (CH_2Ph), 75.2 (C-5), 124.1 (C-Ar), 127.6 (C-Ar), 127.7 (2 x C-Ar), 128.3 (2 x C-Ar), 128.3 (2 x C-Ar), 128.4 (2 x C-Ar), 138.6 (C-Ar), 139.9 (C-Ar),); *m/z* (ESI) 407.1994 (MNa^+, 100%, $C_{23}H_{32}O_3NaSi$ requires 407.2018).

(1S*,3S*,5R*)-3-*O*-Acetyl-1-(benzyldimethylsilyl)-5-methyl-tetrahydropyran 13

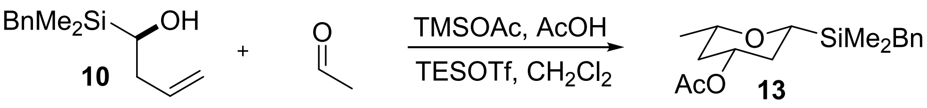

To a solution of alcohol **10** (80 mg, 0.36 mmol), acetaldehyde (41 µL, 0.73 mmol) and trimethylsilyl acetate (54 µl, 0.36 mmol) in acetic acid (0.35 mL) was added triethylsilyl trifluoromethanesulfonate (246 µl, 1.09 mmol). This was stirred for 5 minutes, then the reaction mixture was diluted with CH_2Cl_2 (10 mL) and saturated aqueous solution of $NaHCO_3$ (15 mL) added. The aqueous phase was extracted with CH_2Cl_2 (3 x 10 mL), combined, washed with saturated aqueous solution of $NaHCO_3$ (15 mL), dried ($MgSO_4$) and concentrated *in vacuo*. The crude residue was then purified by column chromatography (Pet: Et_2O, 90:10) to give acetate **13** as a yellow oil (81 mg, 73%); ν_{max} (neat)/cm^{-1} 3065 (ArCH), 3020 (ArCH), 2937 (CH), 1739 (C=O), 1600 (ArC=C), 1027 (C-O); δ_H (400 MHz; $CDCl_3$) -0.05 (3H, s, SiCH_3), 0.02 (3H, s, SiCH_3), 1.19 (3H, d, *J* 6.3, 1'-H_3), 1.26 (1H, app. q, *J* 11.5, 4-H_{ax}), 1.41 (1H, app. q, *J* 12.5, 2-H_{ax}), 1.80 (1H, ddd, *J* 12.5, 4.5, 2.0, 2-H_{eq}), 1.95 (1H, ddd, *J* 11.5, 4.5, 2.0, 4-H_{eq}), 2.03 (3H, s, C(O)CH_3), 2.12 (1H, d, *J* 13.5, SiC*H*HPh), 2.23 (1H, d, *J* 13.5, SiCH*H*Ph), 3.01 (1H, dd, *J* 12.5, 2.0, 1-H_{ax}), 3.40 (1H, app. sext. of d., *J* 6.3, 2.0, 5-H_{ax}), 4.82 (1H, tt, *J* 11.0, 5.0, 3-H_{ax}), 7.02 (2H, *J* 8.0, Ar-H), 7.07 (1H, t, *J* 7.5, Ar-H), 7.02 (2H, t, *J* 7.5, Ar-H); δ_C (100 MHz; $CDCl_3$) -6.1 (SiCH_3), -5.7 (SiCH_3), 21.5 (COCH_3), 22.1 (C-1'), 23.1 (SiCH_2Ph), 32.5 (C-2), 39.7 (C-4), 68.1 (C-1), 71.8 (C-3), 74.5 (C-5), 124.2 (C-Ar), 128.3 (2 x C-Ar), 128.4 (2 x C-Ar), 139.8 (C-Ar), 170.7 (*C*O); *m/z* (ESI) 329.1543 (MNa^+, 100%, $C_{17}H_{26}O_3NaSi$ requires 329.1549).

(1*S*,3*S*,5*S*)-3-*O*-Acetyl-1-(benzyldimethylsilyl)-5-(2'-(benzyloxy)ethyl)-tetrahydropyran 14

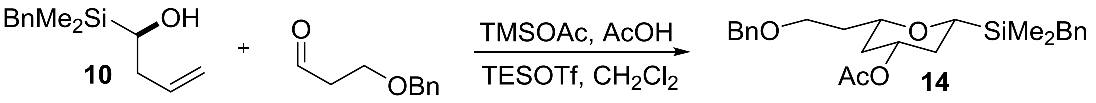

To a solution of alcohol **10** (80 mg, 0.36 mmol), 3-benzyloxypropanal (120 mg, 0.73 mmol) and trimethylsilyl acetate (54 µl, 0.36 mmol) in acetic acid (0.35 mL) was added triethylsilyl trifluoromethanesulfonate (246 µl, 1.09 mmol). This was stirred for 5 minutes, then the reaction mixture was diluted with CH_2Cl_2 (10 mL) and saturated aqueous solution of $NaHCO_3$ (15 mL) added. The aqueous phase was extracted with CH_2Cl_2 (3 x 10 mL), combined, washed with saturated aqueous solution of $NaHCO_3$ (15 mL), dried ($MgSO_4$) and concentrated *in vacuo*. The crude residue was then purified by column chromatography (Pet: Et_2O, 90:10) to give acetate **14** as a yellow oil (110 mg, 71%); ν_{max} (neat)/cm^{-1} 3059 (CH), 2951 (CH), 1738 (C=O), 1599 (C=C), 1026 (C-O); δ_H (400 MHz; $CDCl_3$) -0.06 (3H, s, SiC*H₃*), 0.01 (3H, s, SiC*H₃*), 1.32 (1H, app. q., *J* 12.0, 2-H_{ax}), 1.43 (1H, app. q., *J* 12.0, 4-H_{ax}), 1.75-1.83 (2H, m, 1'-H_2) 1.83 (1H, m, 4-H_{eq}), 1.97 (1H, ddt, *J* 12.0, 4.5, 2.0, 2-H_{eq}), 2.04 (3H, s, C(O)CH_3), 2.10 (1H, d, *J* 13.5, SiC*H*H), 2.20 (1H, d, *J* 13.5, SiCH*H*), 3.11 (1H, dd, *J* 12.0, 1.5, 1-H), 3.49 (1H, m, 5-H), 3.55-3.68 (2H, m, 2'-H_2), 4.52 (2H, s, CH_2Ph), 4.85 (1H, app. tt, *J* 11.0, 4.7, 3-H), 7.02 (2H, *J* 7.5, Ar-H), 7.07 (1H, t, *J* 7.5, Ar-H), 7.02 (2H, t, *J* 7.5, Ar-H) 7.29 (1H, m, ArH), 7.32-7.37 (4H, m, ArH); δ_C (100 MHz; $CDCl_3$) -6.1 (Si*C*H_3), -5.9 (Si*C*H_3), 21.5 (CO*C*H_3), 23.0 (Si*C*H_2), 32.7 (C-4), 36.7 (C-1'), 38.1 (C-2), 66.8 (C-2'), 67.9 (C-1), 71.7 (C-3), 73.1 (*C*H_2Ph), 75.2 (C-5), 124.2 (C-Ar), 127.6 (2 x C-Ar), 127.7 (2 x C-Ar), 128.3 (2 x C-Ar), 128.5 (2 x C-Ar), 138.6 (C-Ar), 139.9 (C-Ar), 170.6 (CO); *m/z* (ESI) 449.2116 (MNa^+, 100%, $C_{25}H_{34}O_4NaSi$ requires 449.2124).

(1*S*,2*R*,3*S*,5*R*)-3-*O*-Acetyl-1-(Benzyldimethylsilane)-2-methyl-5-(2'-phenylethyl)-tetrahydropyran 18

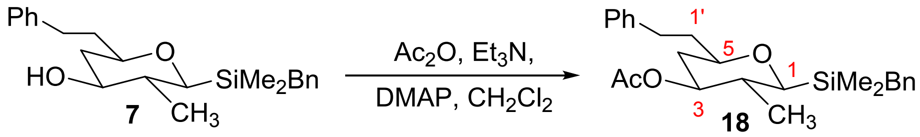

Acetic anhydride (1.52 mL, 2.28 mmol), triethylamine (1.52 mL, 3.80 mmol) and a single crystal of DMAP were added to a solution of alcohol **7** (270 mg, 0.73 mmol) in dry CH_2Cl_2 (8 mL) and stirred at RT under N_2 for 1 h. The reaction was diluted with water (10 mL) and extracted with CH_2Cl_2 (3 x 10 mL). The combined organic phases were washed with saturated aqueous $NaHCO_3$ solution (1 x 10 mL), dried ($MgSO_4$) and concentrated *in vacuo*. Purification by column chromatography (Pet: EtOAc, 98:2) gave acetate **18** as a colourless oil (298 mg, 99%); $[\alpha]_D^{20}$ + 17.6 (*c* 0.97, $CHCl_3$); ν_{max} (neat)/cm^{-1} 3061 (ArCH), 3025 (ArCH), 2928 (CH), 1731 (C=O), 1600 (ArC=C), 1238 (C-O); δ_H (400 MHz; $CDCl_3$) 0.05 (3H, s, SiC*H₃*), 0.08 (3H, s, SiC*H₃*), 0.83 (3H, d, *J* 6.6, 2-C*H₃*), 1.27-1.31 (1H, m, 1'-*H*H), 1.32 (1H, app. q., *J* 11.2, 4-H_{ax}), 1.67-1.89 (2H, m, 2-H_{ax} and 1'-H*H*), 2.01 (1H, ddd, *J* 11.2, 4.6, 1.7, 4-H_{eq}), 2.07 (3H, s, C(O)C*H₃*), 2.22 (1H, d, *J* 13.6, SiC*H*H), 2.30 (1H, d, *J* 13.6, SiCH*H*), 2.67 (1H, m, 2'-*H*H), 2.80 (1H, m, 2'-H*H*), 2.83 (1H, d, *J* 11.4, 1-H), 3.30 (1H, tdd, *J* 11.2, 4.0, 1.7, 5-H), 4.54 (1H, td, *J* 11.2, 4.6, 3-H), 7.04-7.32 (10H, m, ArH); δ_C (100 MHz; $CDCl_3$) -4.8 (Si*C*H₃), -3.7 (Si*C*H₃), 13.5 (2-*C*H₃), 21.2 (C(O)*C*H₃), 23.8 (Si*C*H₂), 31.8 (C-2'), 37.8 (C-4), 37.9 (C-2), 38.0 (C-1'), 74.1 (C-1), 76.9 (C-3), 77.1 (C-5), 124.1 (C-Ar), 125.7 (C-Ar), 128.2 (C-Ar), 128.3 (C-Ar), 128.4 (C-Ar), 128.5 (C-Ar), 139.8 (C-Ar), 142.2 (C-Ar), 170.9 (*C*O); *m/z* (ESI) 433.2170 (MNa^+, 100%, $C_{25}H_{34}NaSiO_3$ requires 433.2169).

(1*S*,3*S*,5*R*)-3-*O*-Acetyl-1-(Benzyldimethylsilane)-5-(2'-phenylethyl)-tetrahydropyran 19

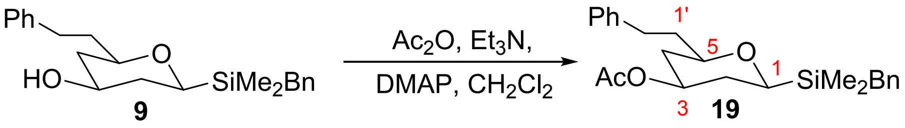

Acetic anhydride (507 µl, 0.76 mmol), triethylamine (605 µl, 1.25 mmol) and a single crystal of DMAP were added to a solution of alcohol **9** (90 mg, 0.25 mmol) in dry CH_2Cl_2 (2 mL) and stirred

at RT under N_2 for 1 h. The reaction was diluted with water (5 mL) and extracted with CH_2Cl_2 (3 x 10 mL). The combined organic phases were washed with saturated aqueous $NaHCO_3$ solution (1 x 10 mL), dried ($MgSO_4$) and concentrated *in vacuo*. Purification by column chromatography (Pet: EtOAc, 95:5) gave acetate **19** as a colourless oil (96 mg, 97%); $[\alpha]_D^{21}$ + 19 (*c* 1.00, $CHCl_3$); ν_{max} (neat)/cm^{-1} 3061 (ArCH), 2923 (CH), 1728 (C=O), 1601 (ArC=C), 1238 (C-O); δ_H (400 MHz; $CDCl_3$) 0.00 (3H, s, SiC*H₃*), 0.06 (3H, s, SiC*H₃*), 1.33 (1H, app. q., *J* 11.3, 4-H_{ax}), 1.47 (1H, app. q., *J* 11.3, 2-H_{ax}), 1.72 (1H, m, 1'-*H*H), 1.82-1.90 (2H, m, 2-H_{eq} and 1'-H*H*), 1.96 (1H, ddd, *J* 11.3, 6.4, 2.0, 4-H_{eq}), 2.05 (3H, s, C(O)C*H₃*), 2.17 (1H, d, *J* 13.7, SiC*H*H), 2.25 (1H, d, *J* 13.7, SiCH*H*), 2.71 (1H, m, 2'-*H*H), 2.82 (1H, m, 2'-H*H*), 3.11 (1H, dd, *J* 11.3, 2.0, 1-H), 3.28 (1H, tdd, *J* 11.3, 3.9, 2.0, 5-H), 4.83 (1H, app. tt, *J* 11.3, 4.9, 3-H), 7.04-7.11 (3H, m, ArH), 7.19-7.5 (7H, m, ArH); δ_C (100 MHz; $CDCl_3$) -6.1 (Si*C*H_3), -5.9 (Si*C*H_3), 21.4 (C(O)*C*H_3), 22.9 (Si*C*H_2), 31.7 (C-2'), 32.7 (C-1') 37.9 (C-4), 37.9 (C-2), 68.0 (C-1), 71.7 (C-3), 77.1 (C-5), 124.1 (C-Ar), 125.7 (C-Ar), 128.2 (2 x C-Ar), 128.2 (2 x C-Ar), 128.3 (2 x C-Ar), 128.4 (2 x C-Ar), 142.2 (2 x C-Ar), 170.5 (*C*O); *m/z* (ESI) 419.2000 (MNa^+, 100%, $C_{25}H_{34}NaSiO$ requires 419.2013).

(1*S*,2*R*,3*S*,5*R*)-3-*O*-Benzyl-1-(Benzyldimethylsilane)-2-methyl-5-(2'-phenylethyl)-tetrahydropyran 20

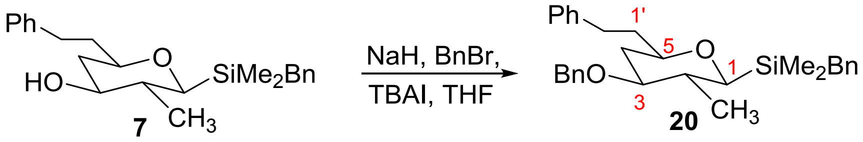

NaH (36 mg, 60% dispersion in oil, 0.88 mmol) was added to a solution of alcohol **7** (80 mg, 0.22 mmol) in dry THF (2 mL), cooled to 0 °C and stirred for 30 minutes under N_2. To the resulting suspension, TBAI (8 mg, 0.02 mmol) and benzyl bromide (150 mg, 0.88 mmol) were added and the reaction was allowed to warm to RT slowly. After stirring for 6 h, NH_4Cl solution (5 mL) was added and the reaction mixture diluted with CH_2Cl_2 (5 mL), separated and the aqueous layer extracted with CH_2Cl_2 (3 x 10 mL). The organic phases were combined and washed with brine (5 mL), dried ($MgSO_4$) and concentrated *in vacuo* to give a crude residue. Purification by column chromatography (EtOAc:Pet, 1:99) gave benzyl ether **20** as a yellow oil (99 mg, 99%); $[\alpha]_D^{23}$ + 29 (*c* 1.00 , $CHCl_3$); δ_H (400 MHz; $CDCl_3$) 0.05 (3H, s, SiC*H₃*), 0.08 (3H, s, SiC*H₃*), 0.96 (3H, d, *J* 6.4, 2-

CH_3), 1.31 (1H, app. q., *J* 11.4, 4-H_{ax}), 1.71-1.19 (2H, m, 2-H and 1'-*H*H), 1.87 (1H, m, 1'-H*H*), 2.11 (1H, ddd, *J* 11.4, 4.6, 2.0, 4-H_{eq}), 2.22 (1H, d, *J* 13.7, SiC*H*H), 2.31 (1H, d, *J* 13.7, SiCH*H*), 2.71 (1H, m, 2'-*H*H), 2.77 (1H, d, *J* 11.0, 1-H), 2.83 (1H, m, 2'-H*H*), 3.07 (1H, td, *J* 11.4, 4.6, 3-H), 3.22 (1H, tdd, *J* 11.4, 3.9, 2.0, 5-H), 4.43 (1H, d, *J* 11.5, C*H*HPh), 4.66 (1H, d, *J* 11.5, CH*H*Ph), 7.05-7.38 (15H, m, ArH); δ_C (100 MHz; $CDCl_3$) -4.8 (SiCH_3), -3.7 (SiCH_3), 13.8 (2-CH_3), 23.9 (SiCH_2), 31.8 (C-2'), 37.5 (C-4), 38.2 (C-1'), 39.2 (C-2), 70.3 (OCH_2Ph), 74.4 (C-1), 76.9 (C-5), 81.3 (C-3), 123.9 (C-Ar), 125.7 (C-Ar), 127.5 (C-Ar), 127.8 (2 x C-Ar), 128.1 (2 x C-Ar), 128.3 (2 x C-Ar), 128.4 (2 x C-Ar), 128.5 (2 x C-Ar), 130.5 (2 x C-Ar), 138.7 (C-Ar), 140.1 (C-Ar), 142.4 (C-Ar); *m/z* (ESI) 481.2521 (MNa^+, 100%, $C_{30}H_{38}NaSiO_2$ requires 481.2539).

(1*S*,3*S*,5*R*)-3-*O*-Benzyl-1-(Benzyldimethylsilane)-5-ethyl-(2'-phenylethyl)-tetrahydropyran 21

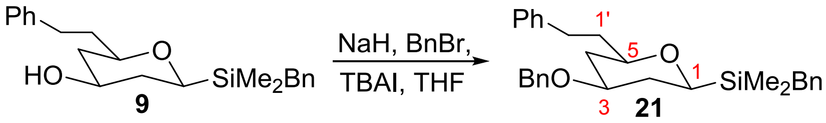

NaH (41 mg, 60% dispersion in oil, 1.02 mmol) was added to a solution of alcohol **9** (90 mg, 0.22 mmol) in dry THF (2.5 mL), cooled to 0 °C and stirred for 30 minutes under N_2. To the resulting suspension, TBAI (9 mg, 0.03 mmol) and benzyl bromide (174 mg, 1.02 mmol) were added and the reaction was allowed to warm to RT slowly. After stirring for 12 h, NH_4Cl solution (10 mL) was added and the reaction mixture diluted with CH_2Cl_2 (10 mL), separated and the aqueous layers extracted with CH_2Cl_2 (3 x 10 mL). The organic phases were combined and washed with brine (10 mL), dried ($MgSO_4$) and concentrated *in vacuo* to give a crude residue. Further purification by column chromatography (EtOAc:Pet, 2:98) gave benzyl ether **21** as yellow oil (110 mg, 97%); δ_H (400 MHz; $CDCl_3$) - 0.01 (3H, s, SiCH_3), 0.06 (3H, s, SiCH_3), 1.28 (1H, app. q., *J* 11.0, 4-H_{ax}), 1.42 (1H, app. q., *J* 12.7, 2-H_{ax}), 1.72 (1H, m, 1'-*H*H), 1.83-1.91 (2H, m, 1'-H*H* and 2-H_{eq}), 2.02 (1H, ddd, *J* 11.0, 4.7, 2.2, 4-H_{eq}), 2.17 (1H, d, *J* 13.7, SiC*H*H), 2.26 (1H, d, *J* 13.7, SiCH*H*) 2.70 (1H, m, 2'-*H*H), 2.82 (1H, m, 2'-H*H*), 3.01 (1H, dd, *J* 12.7, 1.7, 1-H_{ax}), 3.18 (1H, tdd, *J* 11.0, 4.2, 2.2, 5-H), 3.48 (1H, dtt, *J* 12.7, 11.0, 4.7, 3-H_{ax}), 4.55 (2H, s, OCH_2Ph), 7.04-7.10 (3H, m, ArH), 7.19-7.39 (12H, m, ArH); δ_C (100 MHz; $CDCl_3$) -6.0 (SiCH_3), -5.8 (SiCH_3), 23.1 (SiCH_2), 31.8 (C-2'), 33.4 (C-2), 38.1 (C-4), 38.8 (C-1'), 68.1 (C-1), 69.4 (OCH_2Ph), 75.8 (C-3), 77.2 (C-5), 124.0 (C-Ar), 125.7 (C-Ar), 127.5 (C-Ar), 127.6 (2 x C-Ar), 127.8 (2 x C-Ar), 128.2 (2 x C-Ar), 128.3 (2 x C-Ar), 128.4 (2 x

C-Ar), 128.5 (2 x C-Ar), 138.7 (C-Ar), 139.9 (C-Ar), 142.4 (C-Ar); *m/z* (ESI) 467.2361 (MNa^+, 100%, $C_{25}H_{34}NaSiO$ requires 467.2377).

General procedure for the oxidation of 1-silyl tetrahydropyrans

TBAF (1.5 eq, 0.25 M solution in THF) was added dropwise over 30 minutes to a solution of 1-silyl tetrahydropyran (0.1 mmol, 1 eq) in dry THF (1.5 mL) at 0 °C under N_2. Upon warming slowly to 15 °C, disappearance of 1-silyl tetrahydropyran was monitored by TLC. Urea hydrogen peroxide (5 eq), potassium hydrogen carbonate (3 eq) and dry methanol (0.25 mL) were added. This was left to warm to RT for 1 hour, monitored by TLC using phosphomolybdic acid staining, with the lactol visualized as a green spot. On completion aqueous saturated sodium thiosulfate solution (2 mL) was added, the organic phases separated and aqueous layer extracted with CH_2Cl_2 (3 x 5 mL). The crude reaction mixture was concentrated *in vacuo* then taken up in dry CH_2Cl_2 (1.5 mL). The solution was then cooled to 0 °C and triethylamine (5 eq), acetic anhydride (3 eq) and a crystal of DMAP was added. The reaction was stirred for 1h and on completion water (6 mL) was added and the organic phase separated. The aqueous phase was washed with CH_2Cl_2 (3 x 6 mL), the combined organic phases dried ($MgSO_4$) and concentrated *in vacuo*. The residue was then purified by column chromatography (Pet:EtOAc) to yield 1-*O*-acetates as a mixture of α/β anomers, inseparable by column chromatography. Selected data is reported below.

(2*R*,3*S*,5*R*)-1,3-*O*-Acetyl-2-methyl-5-(2'-phenylethyl)-tetrahydropyran 17 (from 18)

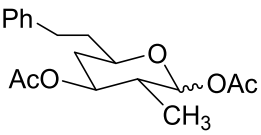

Yellow oil (80 %, α:β 51:49); δ_H (400 MHz; $CDCl_3$) 0.89 (3H, d, *J* 6.8, β-2-CH_3), 0.92 (3H, d, *J* 6.6, α-2-CH_3), 1.30-1.45 (2H, app. q., *J* 11.0, 2 x 4-H_{ax}), 2.05 (β C(O)CH_3), 2.07 (α C(O)CH_3), 2.08 (β C(O)CH_3), 2.18 (α C(O)CH_3), 5.36 (1H, d, *J* 9.0, β 1-H_{ax}), 6.13 (1H, d, *J* 3.5, α 1-H_{eq}); δ_C (100 MHz; $CDCl_3$) 94.7 (C-1), 96.0 (C-1); *m/z* (ESI) 343.1530 (MNa^+, 100%, $C_{18}H_{24}O_5Na$ requires 343.1515).

(3*S*,5*R*)-1,3-*O*-Acetyl-5-(2'-phenylethyl)-tetrahydropyran 22 (from 19)

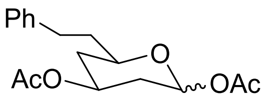

Colorless oil (64 %, α:β 29:71); δ_H (400 MHz; $CDCl_3$) 2.05 (3H, s, 4-C(O)CH_3), 2.15 (3H, s, α-C(O)CH_3), 5.67 (1H, dd, *J* 10.3, 2.5, β 1-H_{ax}), 6.31 (1H, br. d, *J* 2.7, α 1-H_{eq}) ; δ_C (100 MHz; $CDCl_3$) 92.0 (C-1), 92.1 (C-1); *m/z* (ESI) 329.1345 (MNa^+, 100%, $C_{17}H_{22}O_5Na$ requires 329.1359).

(2*R*,3*S*,5*R*)-1-*O*-Acetyl-3-*O*-Benzyl-2-methyl-5-(2'-phenylethyl)-tetrahydropyran 23 (from 20)

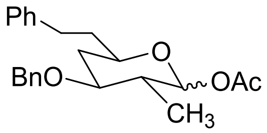

Clear oil (73%, α:β 32:68); δ_H (400 MHz; $CDCl_3$) 2.06 (3H, s, α C(O)CH_3), 2.17 (3H, s, β C(O)CH_3), 5.32 (1H, d, *J* 9.2, α 1-H_{ax}), 6.13 (1H, d, *J* 3.5, β 1-H_{eq}); δ_C (100 MHz; $CDCl_3$) 95.2 (C-1), 96.4 (C-1); *m/z* (ESI) 391.1889 (MNa^+, 100%, $C_{23}H_{31}ONaClSi$ requires 391.1879).

(3*S*,5*R*)-1-*O*-Acetyl-3-*O*-Benzyl-5-(2'-phenylethyl)-tetrahydropyran 24 (from 21)

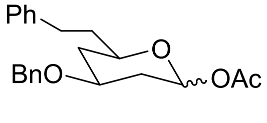

Colorless oil (71 % α:β 37:43); 2.04 (3H, s, α-C(O)CH_3), 2.16 (3H, s, β-C(O)CH_3), 5.61 (1H, dd, *J* 10.0, 2.2, 1-H_{ax}), 6.33 (1H, br. d, *J* 2.5, 1-H_{eq}); δ_C (100 MHz; $CDCl_3$) 92.6 (C-1), 92.7 (C-1); *m/z* (ESI) 377.17171 (MNa^+, 100%, $C_{22}H_{26}O_4Na$ requires 377.1723).

(2*R*)-1,2-Epoxy-3-*O*-(*tert*-butyldiphenylsilane)-propane 25[2]

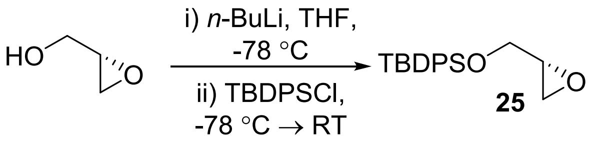

n-BuLi (1.48 M in hexanes, 4.56 mL, 6.78 mmol) was added dropwise to a solution of (*S*)-glycidol (0.45 mL, 6.75 mmol) in THF (14.0 mL) at -78 °C under an atmosphere of N_2. After 20 min, TBDPSCl (1.86 g, 6.75 mmol) was added dropwise. After 5 min, the reaction mixture was warmed to RT and stirred for 72 h. A saturated aqueous solution of NH_4Cl (20 mL) was added and the reaction mixture was extracted with EtOAc (3 x 10 mL). The combined organic extracts were washed with brine (20 mL), dried over $MgSO_4$ and concentrated *in vacuo*. The residue was purified by column chromatography (Pet:EtOAc, 90:10) gave silyl ether **25** as a colourless oil (2.11 g, 99%); $[\alpha]_D^{22}$ + 1.0 (*c* 1.00, $CHCl_3$), lit.[3] $[\alpha]_D^{25}$ + 2.3 (*c* 2.00, $CHCl_3$); δ_H (400 MHz; $CDCl_3$) 1.10 (9H, s, $(CH_3)_3$), 2.64 (1H, dd, *J* 5.1, 2.7, 1-*H*H), 2.77 (1H, dd, *J* 5.1, 4.2, 1-H*H*), 3.18 (1H, m, 2-H), 3.75 (1H, m, 3-*H*H), 3.89 (1H, m, 3-H*H*), 7.32-7.53 (6H, m, ArH), 7.62-7.84 (4H, m, ArH); δ_C (100 MHz; $CDCl_3$) 19.3 ($C(CH_3)_3$), 26.8 ($C(CH_3)_3$), 44.5 (C-1), 52.3 (C-2), 64.3 (C-3), 127.7 (2 x C-Ar), 129.8 (2 x C-Ar), 133.3 (2 x C-Ar), 134.8 (2 x C-Ar), 135.6 (2 x C-Ar). Spectroscopic data in agreement with literature.[2]

(2*R*)-1-*O*-(*tert*-Butyldiphenylsilyl)-4-penten-1,2-diol 26[2]

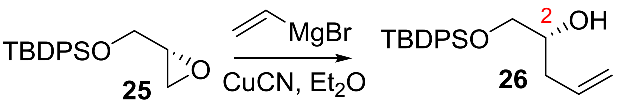

Vinylmagnesium bromide (1.0 M in THF, 7.36 mL, 7.36 mmol) was added dropwise to a solution of CuCN (0.33 g, 3.68 mmol) in Et_2O (5 mL) at -78 °C under an atmosphere of N_2. The reaction mixture was warmed to -60 °C until the CuCN had dissolved. The reaction mixture was cooled to -78 °C and a solution of epoxide **25** (500 mg, 1.60 mmol) in Et_2O (5 mL) added dropwise. The reaction mixture was slowly warmed to -60 °C and stirred for 3 h. The reaction mixture was quenched with a saturated aqueous solution of NH_4Cl (10 mL) and stirred for 25 mins. Et_2O (30

mL) and water (30 mL) were added and the organic phase was separated. The aqueous layer was extracted with Et_2O (2 x 20 mL). The combined organic extracts were washed with brine (40 mL), dried over $MgSO_4$ and concentrated in *vacuo*. Purification by column chromatography (Pet: EtOAc, 90:10) gave alcohol **26** as a colourless oil (482 mg, 89%); $[\alpha]^{25}_{D}$ + 2.5 (*c* 1.0, $CHCl_3$), lit.[2] $[\alpha]^{25}_{D}$ + 3.0 (*c* 0.99, $CHCl_3$); δ_H (400 MHz; $CDCl_3$) 1.10 (9H, s, $(CH_3)_3$), 2.21-2.29 (2H, m, 3-H_2), 3.56 (1H, m, 1-*H*H), 3.68 (1H, m, 1-H*H*), 3.80 (1H, tt, *J* 6.6, 3.7, 2-H), 5.03-5.13 (2H, m, 5-H_2), 5.74-5.87 (1H, m, 4-H), 7.36-7.50 (6H, m, ArH), 7.64-7.72 (4H, m, ArH); δ_C (100 MHz; $CDCl_3$); 18.9 ($C(CH_3)_3$), 26.5 ($C(CH_3)_3$), 37.2 (C-3), 67.0 (C-1), 70.9 (C-2), 117.1 (C-5), 127.4 (2 x C-Ar), 129.5 (2 x C-Ar), 134.0 (C-4), 134.5 (2 x C-Ar), 135.2 (2 x C-Ar). Spectroscopic data in agreement with the literature.[2]

(1*S*,3*S*,5*S*)-3-*O*-Acetyl-5-acetoxymethyl-1-(benzyldimethylsilyl)-tetrahydropyran 27

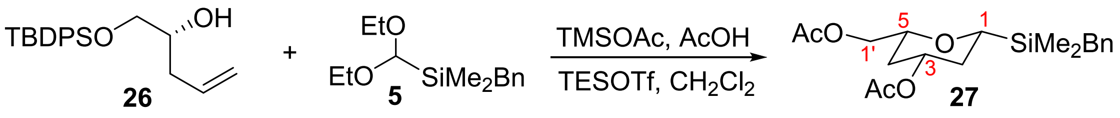

To a solution of homoallylic alcohol **26** (46 mg, 0.14 mmol), silyl acetal **5** (68 mg, 0.27 mmol) and trimethylsilyl acetate (20 µl, 0.14 mmol) in acetic acid (1 mL) was added triethylsilyl trifluoromethanesulfonate (TESOTf) (122 µl, 0.54 mmol). This was stirred for 5 minutes, then the reaction mixture was diluted with CH_2Cl_2 (3 mL) and saturated aqueous solution of $NaHCO_3$ (5 mL) added. The aqueous phase was extracted with CH_2Cl_2 (3 x 5 mL), combined, washed with saturated aqueous solution of $NaHCO_3$ (5 mL), dried ($MgSO_4$) and concentrated *in vacuo*. The crude residue was then purified by column chromatography (Pet: Et_2O, 85:15) to give diacetate **27** as a yellow oil (32 mg, 65%); $[\alpha]^{23}_{D}$ + 37 (c 1.00, $CHCl_3$); ν_{max} (neat)/cm^{-1} 3059 (CH), 2955 (CH), 1737 (C=O), 1600 (C=C), 1027 (C-O); δ_H (400 MHz; $CDCl_3$) 0.01 (3H, s, SiCH_3), 0.09 (3H, s, SiCH_3), 1.39 (1H, app. q., *J* 11.8, 2-H_{ax}), 1.51 (1H, app. q., *J* 12.5, 4-H_{ax}), 1.87 (1H, ddd, *J* 12.5, 4.7, 2.5, 4-H_{eq}), 2.03 (1H, ddt, *J* 11.8, 4.7, 2.5, 2-H_{eq}), 2.09 (3H, s, C(O)CH_3), 2.15 (3H, s, C(O)CH_3), 2.17 (1H, d, *J* 13.5, Si*H*H), 2.29 (1H, d, *J* 13.5, SiCH*H*), 3.14 (1H, dd, *J* 11.8, 2.5, 1-H), 3.60 (1H, m, 5-H), 4.07 (1H, dd, *J* 11.5, 3.9, 1'-*H*H), 4.19 (1H, dd, *J* 11.5, 6.9, 1'-H*H*), 4.90 (1H, app. tt, *J* 11.8, 4.7, 3-H), 7.02-7.09 (3H, m, ArH), 7.18-7.22 (2H, m, ArH); δ_C (100 MHz; $CDCl_3$) -6.3 (SiCH_3), -6.0 (SiCH_3), 20.8 (COCH_3), 21.2 (COCH_3), 22.3 (SiCH_2), 32.3 (C-4), 34.0 (C-2), 66.8 (C-1'), 67.9 (C-1), 71.0 (C-3),

75.8 (C-5), 124.1 (C-Ar), 128.1 (2 x C-Ar), 128.2 (2 x C-Ar), 139.5 (C-Ar), 170.4 (*C*O), 170.8 (*C*O); *m/z* (ESI) 387.1601 (MNa^+, 100%, $C_{19}H_{28}NO_5NaSi$ requires 387.1604).

2,4-Dideoxy-gluc-3,5-diacetate hexopyranose 28

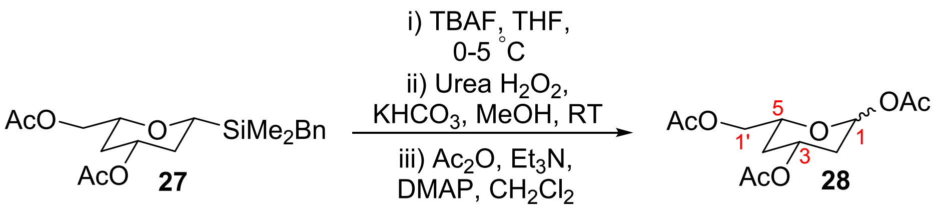

TBAF (1 mL, 0.50 mmol, 0.5 M solution in THF) was added dropwise over 15 minutes to a solution of 1-silyl tetrahydropyran **27** (60 mg, 0.16 mmol, 1 eq) in dry THF (4 mL) at 0 °C under N_2. The reaction was warmed slowly to RT until disappearance of 1-silyl tetrahydropyran **27** by TLC. Urea hydrogen peroxide (78 mg, 0.82 mmol), potassium hydrogen carbonate (50 mg, 0.50 mmol) and dry methanol (1 mL) was added. The reaction was warmed to 40 °C and left to stir for 12 h and upon completion aqueous saturated sodium thiosulphate solution (3 mL) was added, the organic phases separated and aqueous layer extracted with CH_2Cl_2 (3 x 10 mL). The crude reaction mixture was concentrated *in vacuo* then taken up in dry CH_2Cl_2 (5 mL). The solution was then cooled to 0 °C and triethylamine (230 µl, 1.64 mmol), a crystal of DMAP then acetic anhydride (78 µl, 0.82 mmol) was added. The reaction was warmed to RT and left to stir for 1 h and on completion water (12 mL) was added and the organic phase separated. The aqueous phase was washed with CH_2Cl_2 (3 x 15 mL), the combined organic phases dried ($MgSO_4$) and concentrated *in vacuo*. The residue was then purified by column chromatography (Pet:Et_2O, 50:50) to yield **28** as a yellow oil, as a mixture of α/β anomers (26 mg, 57%, α:β 31:69); δ_H (400 MHz; $CDCl_3$) 1.67 (1H, m, 1 x 2-*H*H or 4-*H*H), 1.73-1.83 (4H, m, 4 x 2-*H*H or 4-*H*H), 1.94-2.04 (3H, m, 3 x 2-*H*H or 4-*H*H), 2.08-2.11 (9H, br. m, 3 x C(O)CH_3), 2.12-2.18 (3H, m, C(O)CH_3), 4.09-4.15 (4H, 2 x br. s, 2 x 1'-H_2), 4.18 (1H, m, β 5-H_{ax}), 4.38 (1H, m, α 5-H_{ax}), 5.16 (1H, br. m, α-3-H_{ax}), 5.32 (1H, br. m, β 3-H_{ax}), 6.01 (1H, dd, *J* 9.8, 2.0, β 1-H_{ax}), 6.20 (1H, d, *J* 3.9, α 1-H_{eq}); δ_C (100 MHz; $CDCl_3$) 20.8 (br. C(O)CH_3), 21.2 (br. C(O)CH_3), 29.7, 30.6, 30.9, 31.1, 34.0, 64.2 (α C-5), 65.0 (α C-3), 66.0 (C-1'), 66.2 (C-1'), 67.2 (β C-3), 70.0 (β C-5), 91.0 (β C-1), 91.1 (α C-1), 169.2 (*C*O), 169.4 (*C*O), 170.0 (*C*O), 170.2 (*C*O), 170.8 (*C*O), 170.9 (*C*O); *m/z* (ESI) 297.0940 (MNa^+, 100%, $C_{12}H_{18}O_7Na$ requires 297.0945).

(1*S*,3*S*,5*S*)-1-(Benzyldimethylsilyl)-3-ethoxy-5-((*tert*-butyldiphenylsilyloxy)methyl) – tetrahydropyran 29

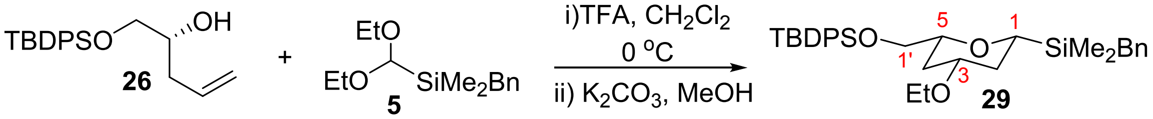

Trifluoroacetic acid (580 µl, 5.96 mmol) was added dropwise to a solution of alcohol **26** (101 mg, 0.30 mmol) and silyl acetal **5** (90 mg, 0.36 mmol) in dry CH_2Cl_2 (4 mL) at RT under N_2 and stirred for 5 mins. Aqueous saturated $NaHCO_3$ (10 mL) was added, the organic phase was separated and the aqueous phase extracted with CH_2Cl_2 (3 x 10 mL). The combined organic phases were dried ($MgSO_4$), then concentrated *in vacuo* to afford the crude residue. This was further purified by column chromatography (Pet: Et_2O, 96:4) gave ethyl ether **29** as a colourless oil (58 mg, 74%); $[\alpha]^{23}_{D}$ + 22 (*c* 0.45, $CHCl_3$); ν_{max} (neat)/cm^{-3} 2937 (C-H), 1596 (C=C), 1090 (C-O), 1070 (C-O), 1003 (C-O); δ_H (400 MHz; $CDCl_3$) -0.03 (3H, s, SiC*H₃*), 0.07 (3H, s, SiC*H₃*), 1.07 (9H, br. s., (C*H₃*)₃), 1.22 (3H, t, *J* 7.1, OCH₂C*H₃*), 1.27 (1H, app. q., *J* 11.3, 4-H_{ax}), 1.36 (1H, app. q., *J* 11.5, 2-H_{ax}), 1.83 (1H, ddd, *J* 11.5, 3.9, 2.0, 2-H_{eq}), 2.04 (1H, ddd, *J* 11.3, 4.4, 2.0, 4-H_{eq}), 2.13 (1H, d, *J* 13.5, SiC*H*H), 2.28 (1H, d, *J* 13.5, SiCH*H*), 3.06 (1H, dd, *J* 11.5, 2.0, 1-H), 3.42 (2H, m, 3-H and 5-H), 3.53 (2H, q, *J* 7.1, OC*H₂*CH₃), 3.63 (1H, dd, *J* 10.5, 4.2, 1'-*H*H), 3.72 (1H, dd, *J* 10.5, 5.6, 1'-H*H*), 7.04-7.22 (5H, m, ArH), 7.37-7.76 (10H, m, ArH); δ_C (100 MHz; $CDCl_3$) -6.2 (Si*C*H₃) -6.0 (Si*C*H₃) 14.1 (OCH₂*C*H₃), 26.8 ((*C*H₃)₃), 28.9 (*C*(CH₃)₃), 33.4 (C-2), 35.0 (C-4), 62.7 (O*C*H₂CH₃), 67.3 (C-1'), 68.0 (C-1), 76.2 (C-3), 79.2 (C-5), 123.9 (3 x C-Ar), 127.6 (2 x C-Ar), 128.1 (C-Ar), 128.3 (2 x C-Ar), 129.5 (2 x C-Ar), 129.6 (2 x C-Ar), 133.8 (C-Ar), 135.6 (2 x C-Ar), 135.7 (2 x C-Ar), 139.9 (C-Ar); *m/z* (ESI) 569.2891 (MNa^+, 100%, $C_{33}H_{46}NaSi_2O_3$ requires 569.2883).

(2*R*)-1,2-Epoxy-3-*O*-(benzyl)-propane Si-2

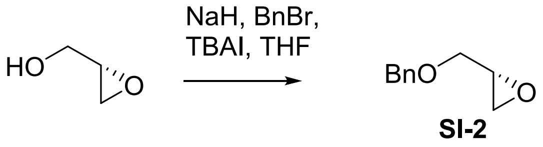

To a solution of (*S*)-glycidol (896 µl, 1.0 g, 13.5 mmol) in THF (130 mL) was added NaH (2.15 g, 60% dispersion in oil, 54.0 mmol) at 0 °C under N_2. The resulting mixture was stirred for 30 minutes, after which benzyl bromide (6.41 mL, 54.0 mmol) and *tert*-butylammonium iodide (498 mg, 1.35 mmol) were added. The reaction mixture was allowed to warm to RT and stirred overnight. Saturated aqueous solution of NH_4Cl (130 mL) was then added. The organic phases were extracted with EtOAc (3 x 100 mL), combined, dried ($MgSO_4$) and concentrated *in vacuo*. The crude residue was then purified by column chromatography (Pet: Et_2O, 86: 14) to give epoxide **SI-2** an colourless oil (2.09 g, 94%); $[\alpha]_D^{25}$ -5.0 (*c* 0.97, $CHCl_3$), lit.[4] $[\alpha]_D^{25}$ – 6.8 (*c* 1.0, $CHCl_3$); δ_H (400 MHz; $CDCl_3$) 2.54 (1H, dd, *J* 2.7, 5.4, 1-*H*H), 2.72 (1H, d, *J* 4.9, 1-H*H*), 3.11 (1H, m, 2-H), 3.37 (1H, dd, *J* 11.5, 5.9, 3-*H*H), 3.69 (1H, dd, *J* 11.5, 2.9, 3-H*H*), 4.48 (1H, d, *J* 12.0, C*H*HPh), 4.54 (1H, d, *J* 12.0, CH*H*Ph), 7.18-7.29 (5H, m, ArH); δ_C (100 MHz; $CDCl_3$) 44.2 (C-1), 50.8 (C-2), 70.8 (CH_2Ph), 73.3 (C-3), 127.7 (C-Ar), 128.4 (C-Ar), 137.8 (C-Ar). Data in accordance with literature.[5]

(2*R*)-1-*O*-(Benzyl)-4-pentene-1,2-diol SI-3

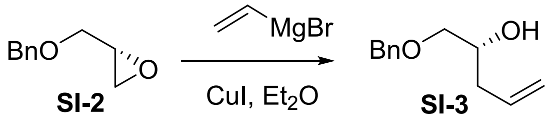

To a solution of epoxide **SI-2** (1.4 g, 9.74 mmol) in Et_2O (100 mL) and copper (I) iodide (185 mg, 0.97 mmol) at -78 °C under N_2 was added vinyl magnesium bromide (10.7 mL of 1M solution in THF, 10.7 mmol) dropwise over 5 minutes. The resulting mixture was allowed to stir for 4 h, after which saturated aqueous solution of NH_4Cl (50 mL) was added. The organics were separated and the aqueous layer extracted with Et_2O (3 x 50 mL), combined, then dried ($MgSO_4$) and concentrated *in vacuo*. The crude residue was then purified by column chromatography (85:15, Hexanes: Et_2O) to give alcohol **SI-3** an colourless oil (1.85 g, 99%); $[\alpha]_D^{25}$ - 6.0 (*c* 1.5, $CHCl_3$), lit.[6] $[\alpha]_D^{25}$ - 4.98 (*c* 1.0, $CHCl_3$); δ_H (400 MHz; $CDCl_3$) 2.19 (2H, m, 3-H_2), 3.31 (1H, dd, *J* 9.5, 7.4, 1-*H*H), 3.44 (1H, *J* 9.5 1.7, 1-*H*H), 3.81 (1H, m, O*H*), 3.94 (1H, m, 2-H). 4.49 (2H, s, C*H₂*Ph), 5.02 (1H, dd, *J* 10.1, 1.9, 5-*H*H), 5.05 (1H, dd, *J* 17.2, 1.9, 5-*H*H), 5.75 (1H, m, 4-H), 7.20-7.31 (5H, m, ArH); δ_C (100 MHz; $CDCl_3$) 37.9 (C-3), 69.7 (C-2), 73.3 (CH_2Ph), 73.8 (C-1), 117.7 (C-5), 127.6

(C-Ar), 127.7 (C-Ar), 128.4 (C-Ar), 134.2 (C-4), 137.9 (C-Ar). Data in accordance with the literature.[7]

(1*S*,3*S*,5*S*)-3-*O*-Acetyl-1-(benzyldimethylsilyl)-5-(1'-(benzyloxy)methyl)-tetrahydropyran 30

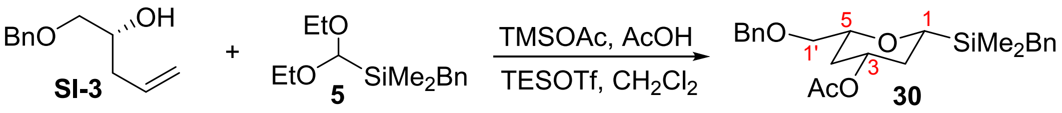

To a solution of alcohol **SI-3** (46 mg, 0.14 mmol) and silyl acetal **5** (68 mg, 0.27 mmol) and trimethylsilyl acetate (20 µl, 0.14 mmol) in acetic acid (1 mL) was added triethylsilyl trifluoromethanesulfonate (TESOTf) (122 µl, 0.54 mmol). This was stirred for 5 minutes, then the reaction mixture was diluted with CH_2Cl_2 (3 mL) and saturated aqueous solution of $NaHCO_3$ (5 mL) was carefully added. The aqueous phase was extracted with CH_2Cl_2 (3 x 5 mL), combined, washed with saturated aqueous solution of $NaHCO_3$ (5 mL), dried ($MgSO_4$) and concentrated *in vacuo*. The crude residue was then purified by column chromatography (91: 9, Hexanes: Et_2O) to give acetate **30** as yellow oil (42 mg, 72%); $[\alpha]_D^{23}$ + 28.3 (*c* 1.27, $CHCl_3$); ν_{max} (neat)/cm^{-1} 3061 (CH), 2856 (CH), 1738 (C=O), 1600 (C=C), 1026 (C-O); δ_H (500 MHz; $CDCl_3$) -0.03 (3H, s, SiC*H₃*), 0.04 (3H, s, SiC*H₃*), 1.38 (1H, app. q., *J* 12.5, 4-H_{ax}), 1.46 (1H, app. q., *J* 12.5, 2-H_{ax}), 1.83 (1H, ddt, *J* 12.5, 6.6, 2.0, 2-H_{eq}), 2.01 (1H, ddt, *J* 12.5, 6.6, 2.2, 4-H_{eq}), 2.04 (3H, s, C(O)CH_3), 2.14 (1H, d, *J* 13.8, SiC*H*H), 2.24 (1H, d, *J* 13.8, SiCH*H*), 3.14 (1H, dd, *J* 12.5, 2.2, 1-H), 3.47 (1H, dd, *J* 13.3, 6.9, 1'-*H*H), 3.53-3.60 (2H, m, 1'-H*H* and 5-H), 4.61 (2H, s, CH_2Ph), 4.81 (1H, app. tt, *J* 12.5, 6.6, 3-H), 7.03-7.11 (3H, m, ArH), 7.19-7.23 (2H, m, ArH), 7.28-7.38 (5H, m, ArH); δ_C (100 MHz; $CDCl_3$) -6.2 (Si*C*H_3), -5.9 (Si*C*H_3), 21.3 (C(O)*C*H_3), 22.9 (Si*C*H_2), 32.6 (C-2), 34.4 (C-4), 68.1 (C-1), 71.5 (C-1'), 73.3 (O*C*H_2Ph), 73.3 (C-5), 77.9 (C-3), 124.1 (2 x C-Ar), 127.5 (2 x C-Ar), 128.2 (2 x C-Ar), 128.3 (2 x C-Ar), 128.4 (2 x C-Ar), 138.5 (C-Ar), 139.5 (C-Ar), 170.6 (*C*O); *m/z* (ESI) 435.1842 (MNa^+, 100%, $C_{24}H_{32}O_4NaSi$ requires 435.1962).

(3*R*, 5*R*)-1,3-*O*-Acteyl-5-(1'-benzyloxymethyl)-tetrahydropyran 31

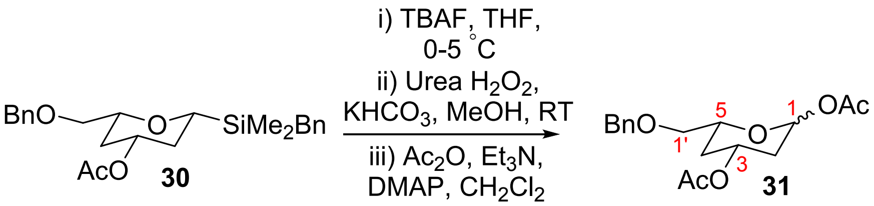

TBAF (2.1 mL, 1.04 mmol, 0.5 M solution in THF) was added dropwise over 15 minutes to a solution of 1-silyl tetrahydropyran **30** (143 mg, 0.35 mmol, 1 eq) in dry THF (2 mL) at 0 °C under N_2. The reaction was warmed slowly to room temperature until disappearance of 1-silyl tetrahydropyran **30** by TLC. Urea hydrogen peroxide (163 mg, 1.74 mmol), potassium hydrogen carbonate (104 mg, 1.04 mmol) and dry methanol (0.7 mL) was added. This was left to stir at RT for 12 h and upon completion aqueous saturated sodium thiosulphate solution (3 mL) was added, the organic phase separated and aqueous layer extracted with CH_2Cl_2 (3 x 5 mL). The crude reaction mixture was concentrated *in vacuo* then taken up in dry CH_2Cl_2 (3 mL). The solution was then cooled to 0 °C and triethylamine (420 µl, 3.00 mmol), a crystal of DMAP and acetic anhydride (190 µl, 2.00 mmol) was added. The reaction was warmed to RT and left to stir for 1 h and on completion water (6 mL) was added and the organic phase separated. The aqueous phase was washed with CH_2Cl_2 (3 x 10 mL), the combined organic phases dried ($MgSO_4$) and concentrated *in vacuo*. The residue was then purified by column chromatography (Pet:Et_2O, 75:25) to yield **31** as a colourless oil, as mixture of α/β anomers; (77 mg, 67% α: β 39:69); δ_H (400 MHz; $CDCl_3$) 1.67- 1.88 (6H, m, 2 x 2-*H*H and 2 x 4-H_2), 1.94-2.04 (2H, m, 2 x 2-H*H*), 2.06 (6H, br. s., 2 x C(O)C*H*$_3$), 2.10 (3H, s, α C(O)C*H*$_3$), 2.11 (3H, s, β C(O)C*H*$_3$), 3.48-3.62 (4H, m, 2 x 1'-H_2), 4.11 (1H, m, β 5-H_{ax}), 4.35 (1H, m, α 5-H_{ax}), 4.54-4.60 (4H, m, 2 x C*H*$_2$Ph), 5.15 (1H, dd, *J* 6.4, 4.8, α 3-H_{ax}) 5.21 (1H, dd, *J* 7.7, 4.4, β 3-H_{ax}), 5.71 (1H, dd, *J* 9.9, 2.1, β 1-H_{ax}), 6.33 (1H, d, *J* 2.8, α 1-H_{eq}), 7.17-7.35 (10H, m, ArH); δ_C (100 MHz; $CDCl_3$) {21.1, 21.1, 21.2, 21.3} (C(O)*C*H$_3$), {33.0, 33.0, 32.4, 35.8} (C-2 and C-4), 69.2 (C-1'), 71.9 (C-1'), 72.1 (C-5), 72.1 (C-5), 72.3 (C-3), 73.4 (C-3), 92.1 (α C-1), 92.4 (β C-1), 127.7 (C-Ar), 128.3 (C-Ar), 129.7 (C-Ar), 137.9 (C-Ar), 168.9 (2 x *C*O), 170.1 (2 x *C*O); *m/z* (ESI) 345.1305 (MNa^+, 100%, $C_{17}H_{22}O_6Na$ requires 345.1309).

(1R,3*S*,5*S*)-3-*O*-Acetyl-1-O-cyclohexyl-5-(1'-(benzyloxy)methyl)-tetrahydropyran 32

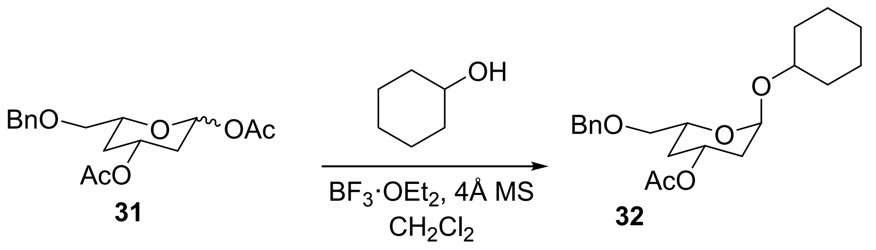

BF3·OEt$_2$ (37 µL, 0.30 mmol) was added dropwise to a solution of acetate **31** (65 mg, 0.20 mmol), cyclohexanol (32 µL, 0.30 mmol) and 4 Å molecular sieves (50 mg) in dry CH_2Cl_2 (5 mL) under N_2. The reaction was stirred for 4 h when a saturated aqueous solution of $NaHCO_3$ (15 mL) was added. The aqueous phase was extracted with CH_2Cl_2 (3 x 10 mL), combined, washed with saturated aqueous solution of $NaHCO_3$ (15 mL), dried ($MgSO_4$) and concentrated *in vacuo*. The crude residue was then purified by column chromatography (Pet: Et_2O, 90:10) to give acetate **31** as a colourless oil, as the α anomer (52 mg, 72%); ν_{max} (neat)/cm^{-1} 2930 (CH), 2856 (CH), 17401 (C=O), 1452 (C-C), 1025 (C-O); δ_H (400 MHz; $CDCl_3$) 1.15-1.31 (4H, m, 4 x CyHex CH) 1.35 (1H, m, CyHex CH), 1.46 (1H, app. q, *J* 12.0, 4-H$_{ax}$), 1.52 (1H, m, CyHex CH), 1.66 (1H, app. dt, *J* 11.5, 3.5, 2-H$_{ax}$), 1.67-1.77 (2H, m, 2 x CyHex CH), 1.81-1.91 (2H, m, 2 x CyHex CH), 1.97-2.06 (5H, m, 2-H$_{eq}$, 4-H$_{eq}$ and C(O)C*H*$_3$) 3.49 (1H, dd, *J* 10.5, 4.0, 1'-*H*H), 3.52 (1H, dd, *J* 10.5, 5.0, 1'-H*H*), 3.57 (1H, m, CyHex CH), 4.10 (1H, dtd, *J* 11.5, 4.5, 2.0, 5-H) 4.56 (1H, s, C*H*HPh), 4.57 (1H, s, C*H*HPh), 5.17 (1H, d, *J* 3.5, 1-H$_{eq}$) 5.22 (1H, app. tt, *J* 11.5, 5.0, 3-H), 7.25-7.30 (1H, m, ArH), 7.31-7.36 (4H, m, ArH); δ_C (100 MHz; $CDCl_3$) 21.5 (C(O)*C*H$_3$), 24.1 (CH_2-CyHex), 24.4 (CH_2-CyHex), 25.8 (CH_2-CyHex), 31.6 (CH_2-CyHex), 33.6 (CH_2-CyHex), 33.9 (C-4), 36.2 (C-2), 66.9 (C-5), 67.4 (C-3), 73.0 (C-1'), 73.4 (O*C*H$_2$Ph), 74.6 (CH-CyHex), 95.7 (C-1), 127.6 (2 x C-Ar), 127.7 (C-Ar), 128.4 (2 x C-Ar), 138.4 (C-Ar), 170.5 (*C*O); *m/z* (ESI) 385.1986 (MNa$^+$, 100%, $C_{21}H_{30}O_5Na$ requires 385.1985).

(1*R*, 3*R*, 5*R*)-1-(Benzyldimethylsilyl)-5-(1'-benzyloxymethyl)-3-hydroxy-tetrahydropyran SI-4

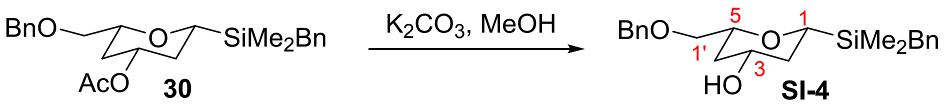

Potassium carbonate (149 mg, 1.08 mmol) was added to a solution of acetate **30** (148 mg, 0.36 mmol) in methanol (4 mL) at RT. After stirring for 10 minutes, the methanol was removed under reduced pressure, water (5mL) added and extracted with CH_2Cl_2 (3 x 5 mL). The combined organic phases were dried ($MgSO_4$) and concentrated *in vacuo* to give alcohol **SI-4** as a colourless oil (130 mg, 98%); $[\alpha]_D^{23}$ + 29 (*c* 1.00, $CHCl_3$); δ_H (400 MHz; $CDCl_3$) -0.02 (3H, s, $SiCH_3$), 0.05 (3H, s, Si*CH₃*), 1.25 (1H, app. q., *J* 12.2, 4-H_{ax}), 1.35 (1H, app. q., *J* 12.5, 2-H_{ax}), 1.79 (1H, ddd, *J* 12.5, 4.5, 2.0, 2-H_{eq}), 2.00 (1H, ddd, *J* 12.2, 4.5, 2.0, 4-H_{eq}), 2.14 (1H, d, *J* 13.7, SiC*H*H), 2.25 (1H, d, *J* 13.7, SiCH*H*), 3.08 (1H, dd, *J* 12.5, 2.0, 1-H), 3.45-3.51 (2H, m, 1'-H_2), 3.58 (1H, app. br. dtd, *J* 12.5, 5.7, 2.0, 5-H), 3.75 (1H, app. tt, *J* 12.5, 4.5, 3-H), 4.62 (2H, s, CH_2Ph), 7.04-7.09 (3H, m, ArH), 7.19-7.23 (2H, m, ArH), 7.28-7.39 (5H, m, ArH); δ_C (100 MHz; $CDCl_3$) -6.1 ($SiCH_3$), -5.9 ($SiCH_3$), 23.0 ($SiCH_2$), 36.3 (C-2), 38.4 (C-4), 68.3 (C-3), 69.1 (C-1), 73.4 (C-1'), 73.5 (OCH_2Ph), 78.0 (C-5), 124.0 (2 x C-Ar), 127.5 (2 x C-Ar), 127.5 (2 x C-Ar), 128.3 (2 x C-Ar), 128.4 (C-Ar), 128.5 (C-Ar), 138.7 (C-Ar), 139.8 (C-Ar); *m/z* (ESI) 393.1842 (MNa^+, 100%, $C_{22}H_{30}O_3NaSi$ requires 493.1862).

(1*R*, 3*S*, 5*R*)-1-(Benzyldimethylsilyl)-5-(1'-benzyloxymethyl)-3-hydroxy-tetrahydropyran 33

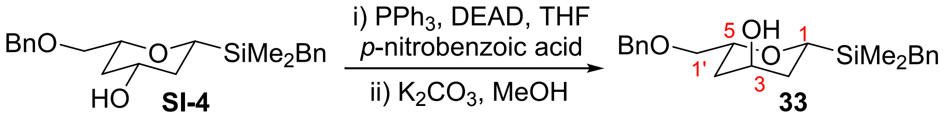

Diethyl azodicarboxylate (94 µl, 0.60 mmol) was added dropwise to a solution of triphenylphosphine (157 mg, 0.60 mmol) and *para*-nitrobenzoic acid (100 mg, 0.60 mmol) in THF (2 mL) at 0 °C.Alcohol **SI-4** (75 mg, 0.20 mmol) was added as a solution in THF (1 mL) dropwise and allowed to warm to RT over 1 h. The solvent was removed *in vacuo* and the resulting crude residue was filtered through a SiO_2 plug (Et_2O:Pet. 15:85). The solvent was removed *in vacuo* to afford a crude residue, which was taken up in methanol (3 mL) and potassium carbonate (55 mg,

0.4 mmol) added. After stirring for 10 minutes at RT, the methanol was removed under reduced pressure, water (5mL) added and extracted with CH_2Cl_2 (3 x 5 mL). The combined organic phases were dried ($MgSO_4$) and concentrated *in vacuo* to afford alcohol **33** as a colourless oil (64 mg, 87%); $[\alpha]_D^{23}$ +22 (*c* 1.00, $CHCl_3$); δ_H (400 MHz; $CDCl_3$) -0.03 (3H, s, SiCH_3), 0.03 (3H, s, SiCH_3), 1.46 (1H, ddd, *J* 13.2, 4.9, 2.2, 4-H_{ax}), 1.57-1.68 (2H, m, 2-H_2), 1.74 (1H, app. dd, *J* 13.2, 2.7, 4-H_{eq}), 2.14 (1H, d, *J* 13.5, SiC*H*H), 2.25 (1H, d, *J* 13.5, SiCH*H*), 3.45 (1H, dd, *J* 10.7, 4.4, 1'-*H*H), 3.52 (1H, *J* 10.7, 5.7, 1'-H*H*), 3.60 (1H, dd, *J* 13.2, 2.1, 1-H), 3.91 (1H, app. br. dtd, *J* 13.2, 5.7, 2.7, 5-H), 4.23 (1H, app. t, *J* 2.7, 3-H_{eq}), 4.62 (2H, s, CH_2Ph), 7.03-7.10 (3H, m, ArH), 7.17-7.24 (2H, m, ArH), 7.28-7.39 (5H, m, ArH); δ_C (100 MHz; $CDCl_3$) -6.2 (SiCH_3), -5.7 (SiCH_3), 23.0 (SiCH_2), 33.6 (C-2), 35.6 (C-4), 63.6 (C-1), 63.7 (C-3), 72.5 (C-5), 73.3 (C-1'), 73.9 (OCH_2Ph), 124.0 (2 x C-Ar), 127.4 (2 x C-Ar), 127.5 (2 x C-Ar), 128.1 (2 x C-Ar), 128.3 (2 x C-Ar), 138.7 (C-Ar), 139.9 (C-Ar); *m/z* (ESI) 393.1842 (MNa^+, 100%, $C_{22}H_{30}O_3NaSi$ requires 493.1862).

(3*S*,5*R*)-1,3-*O*-Acetyl-5-(1'benzyloxymethyl)-tetrahydropyran 34

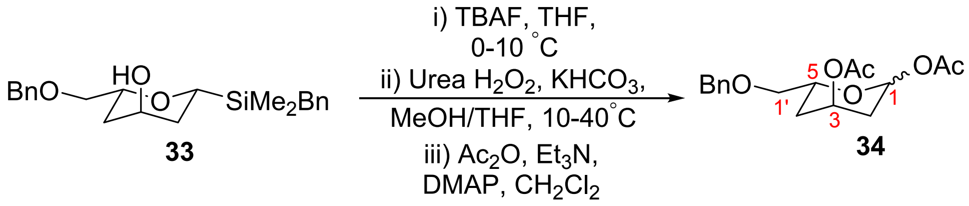

TBAF (800 µl, 0.4 mmol, 0.5 M solution in THF) was added dropwise over 15 minutes to a solution of 1-silyl tetrahydropyran **33** (50 mg, 0.14 mmol, 1 eq) in dry THF (3 mL) at 0 °C under N_2. The reaction was warmed slowly to RT until disappearance of 1-silyl tetrahydropyran **33** by TLC. Urea hydrogen peroxide (64 mg, 0.68 mmol), potassium hydrogen carbonate (56 mg, 0.41 mmol) and dry methanol (1 mL) was added. This was left to stir at 40 °C for 12 h and upon completion aqueous saturated sodium thiosulphate solution (3 mL) was added, the organic phase separated and aqueous layer extracted with CH_2Cl_2 (3 x 5 mL). The crude reaction mixture was concentrated *in vacuo* then taken up in dry CH_2Cl_2 (5 mL). The solution was then cooled to 0 °C and triethylamine (189 µl, 1.4 mmol), a crystal of DMAP then acetic anhydride (65 µl, 0.68 mmol) was added. The reaction was warmed to RT and left to stir for 1 h and on completion water (5 mL) was added and the organic phase separated. The aqueous phase was washed with CH_2Cl_2 (3 x 10 mL), the combined organic phases dried ($MgSO_4$) and concentrated *in vacuo*. The residue was then purified by column chromatography (Pet:Et_2O, 75:25) to yield diacetate **34** as a

colourless oil and mixture of α/β anomers (33 mg, 75%, α:β 33:67); δ_H (400 MHz; $CDCl_3$) 1.68-1.87 (6H, m, 2 x 2-*H*H and 2 x 4-H_2), 1.97-2.03 (2H, m, 2 x 2-H*H*), 2.04 (3H, s, α C(O)CH_3), 2.08 (6H, br. s., 2 x C(O)CH_3), 2.10 (3H, s, β C(O)CH_3), 3.50-3.58 (4H, m, 2 x 1'-H_2), 4.13 (1H, dqd, *J* 11.0, 4.9, 3.4, β 5-H_{ax}), 4.35 (1H, dqd, *J* 15.2, 4.7, 1.0, α 5-H_{ax}), 4.54-4.60 (4H, m, 2 x CH_2Ph), 5.15 (1H, q, *J* 3.2, α 3-H_{eq}) 5.32 (1H, q, *J* 3.2, β 3-H_{eq}), 6.01 (1H, dd, *J* 9.8, 2.5, β 1-H_{ax}), 6.20 (1H, d, *J* 3.9, α 1-H_{eq}), 7.28-7.37 (10H, m, ArH); δ_C (100 MHz; $CDCl_3$) {21.1, 21.2, 21.2, 21.3} (C(O)CH_3), {31.0, 31.3, 31.4, 34.2} (C-4 and C-2), {65.3, 65.6, 67.6, 71.3, 72.1, 72.4, 73.4, 73.5} (C-1, C-1', C-5 and C-3), 91.2 (β-C-1), 91.4 (α-C-1), 127.6 (2 x C-Ar), 127.7 (2 x C-Ar), 127.8 (2 x C-Ar), 128.4 (2 x C-Ar), 137.0 (2 x C-Ar), 169.2 (*C*O), 19.5 (*C*O), 170.1 (*C*O), 170.3 (*C*O); *m/z* (ESI) 345.1308 (MNa^+, 100%, $C_{17}H_{22}O_6Na$ requires 345.1309).

(3*R*,4*R*)-3-Hydroxy-4-ethoxy-1-phenyl-hex-5-en 35

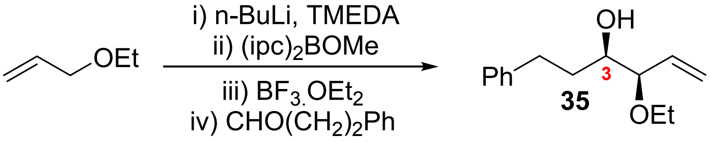

To a solution of allyl ethyl ether (395 µl, 3.49 mmol) in THF (5 mL) and tetramethylethylenediamine (420 µl, 2.79 mmol) was added *n*-butyllithium (1.84 mL, 1.52 M solution in hexanes, 2.79 mmol) at -78 °C under N_2. After stirring at -78 °C for 30 minutes, (+)-*B*-methoxydiisopinocampheylborane (881 mg, 2.79 mmol) in THF (1 mL) was added dropwise and the solution cleared. The reaction was stirred at -78 °C for 1 h, then $BF_3.OEt_2$ (570 µl, 4.64 mmol) was added and immediately followed by dihydrocinnamaldehyde (370 µl, 2.29 mmol). This was left to react for 4h at -78 °C, then saturated aqueous $NaHCO_3$ (10 mL) was added and the organics were extracted with Et_2O (3 x 10 mL). The combined organic phases were washed with 1M HCl (10 mL), dried ($MgSO_4$) and concentrated *in vacuo* to afford the crude residue. Purification by column chromatography (Pet: Et_2O, 93:7) gave alcohol **35** as a colourless oil (190 mg, 32%); $[\alpha]_D^{23}$ + 8.0 (*c* 0.56, $CHCl_3$); δ_H (400 MHz; $CDCl_3$) 1.21 (3H, t, *J* 6.9, CH_3), 1.67-1.82 (2H, m, 2-H_2), 2.67 (1H, dt, *J* 13.5, 8.3, 1-*H*H), 2.80-2.93 (2H, m, 3-H and 1-H*H*), 3.35 (1H, app p, *J* 8.9, 6.9, C*H*HCH_3), 3.48 (1H, m, 4-H), 3.62 (1H, app p., *J* 6.9, CH*H*CH_3), 5.26 (1H, dd, *J* 11.5, 1.8, 6-*H*H), 5.31 (1H, dd, *J* 8.8, 1.8, 6-H*H*), 5.62 (1H, ddd, *J* 11.5, 8.8, 1.8, 5-H); δ_C (100 MHz; $CDCl_3$) 15.2

(CH_3), 31.8 (C-2), 34.3 (C-1), 64.1 (CH_2CH_3), 72.5 (C-3), 85.1 (C-4), 119.7 (C-6), 125.7 (C-5), 128.3 (C-Ar), 128.5 (C-Ar), 135.5 (C-Ar), 142.5 (C-Ar). Spectral data in accordance with the literature.[8]

(1*S*,3*R*,4*R*)-1-(Benzyldimethylsilyl)-4-(2'-phenylethyl)-tetrahydrofuran-3-al 36

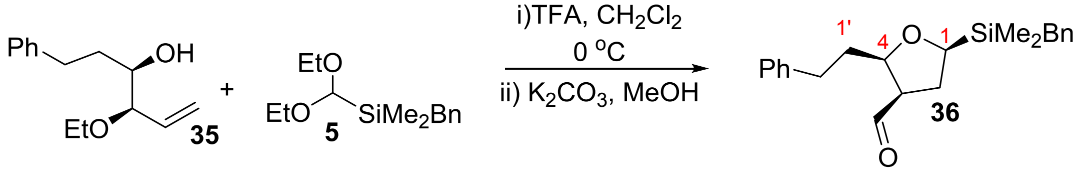

Trifluoroacetic acid (121 mg, 0.55 mmol) was added carefully dropwise to a solution of alcohol **35** (80 mg, 0.36 mmol) and silyl acetal **5** (110 mg, 0.44 mmol) in dry CH_2Cl_2 (3 mL) at 0 °C under N_2. This was stirred for 1 h whilst slowly warming to RT. Saturated aqueous $NaHCO_3$ (10 mL) was added and the organics were extracted with CH_2Cl_2 (3 x 10 mL). The combined organic phases were then dried ($MgSO_4$) and concentrated *in vacuo* to afford a brown residue. Purification by column chromatography (Pet: Et_2O, 93:7) gave aldehyde **36** as a colourless oil (59 mg, 46%); $[\alpha]_D^{23}$ + 14 (c 1.00, $CHCl_3$); ν_{max} (neat)/cm^{-1} 3082 (CH), 1600 (C=C), 1719 (C=O), 1029 (C-O); δ_H (400 MHz; $CDCl_3$) 0.04 (3H, s, SiCH_3), 0.11 (3H, s, SiCH_3), 1.85 – 1.92 (2H, m, 1'-H_2), 2.00-2.07 (2H, m, 2-H_2), 2.21 (1H, d, *J* 12.7, SiC*H*H), 2.26 (1H, d, *J* 12.7, SiCH*H*), 2.70 (1H, m, 2'-*H*H), 2.85 (1H, m, 2'-H*H*), 2.95 (1H, ddd, *J* 14.7, 7.1, 4.4, 3-H), 3.35 (1H, dd, *J* 10.8, 7.4, 1-H), 3.89 (1H, td, *J* 8.2, 7.4, 4-H), 7.05-7.31 (10H, m, ArH), 9.6 (1H, d, *J* 4.4, C*H*O); δ_C (100 MHz; $CDCl_3$) -5.8 (SiCH_3), -5.8 (SiCH_3), 23.5 (SiCH_2Ph), 28.8 (C-2), 32. 8 (C-2'), 33.1 (C-1'), 54.8 (C-3), 70.8 (C-1), 82.3 (C-4), 124.2 (C-Ar), 125.9 (C-Ar), 128.2 (2 x C-Ar), 128.3 (2 x C-Ar), 128.4 (2 x C-Ar), 128.5 (2 x C-Ar), 136.7 (C-Ar), 139.4 (C-Ar), 202.7 (*C*HO); *m/z* (ESI) 375.1747 (MNa^+, 100%, $C_{22}H_{28}O_2NaSi$ requires 375.1751).

(2*S*,3*R*)-1,2-Epoxy-3-hydroxy-pent-4-ene 43[9]

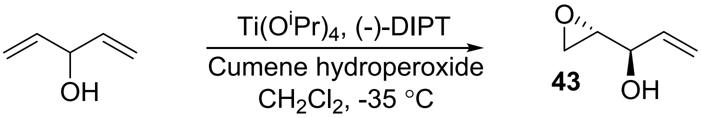

Titanium tetraisopropoxide (1.40 mL, 4.73 mmol) followed by (*R*,*R*)-(-)-diisopropyl D-tartrate (1.32 mL, 6.31 mmol) was added to a solution of 4 Å molecular sieves (800 mg) in dry CH_2Cl_2 (48 mL) at -35 °C. This was stirred for 30 minutes then 1,4-pentadiene-3-ol (4.64 mL, 47.71 mmol) was added, followed by cumene hydroperoxide (18.09 mL, 122.39 mmol). The reaction was stirred for 36 h at – 35 °C then filtered through a SiO_2 plug, washing with CH_2Cl_2 (3 x 30 mL). Aqueous saturated sodium thiosulphate (20 mL) was added, the organic layer separated and the aqueous layer extracted with CH_2Cl_2 (3 x 10 mL). The organic layers were combined, dried ($MgSO_4$) and concentrated *in vacuo* to give yellow oil. This was purified by column chromatography (Pentane: Et_2O, 60:40) to remove excess cumene alcohol and cumene hydroperoxide. Kugelrohr distillation (120 °C, 30 mm/Hg) gave epoxide **43** as a colourless oil (2.62 g, 55%); $[\alpha]_D^{23}$ -55 (*c* 1.00, $CHCl_3$), lit. $[\alpha]_D^{25}$ -53 (*c* 0.73, $CHCl_3$); δ_H (400 MHz; $CDCl_3$) 2.17 (1H, br. s., O*H*), 2.77 (1H, dd, *J* 5.0, 4.2, 1-*H*H), 2.81 (1H, dd, *J* 5.0, 2.8, 1-H*H*), 3.10 (1H, ddd, *J* 6.1, 4.2, 2.8, 2-H), 4.32 (1H, br. m, 3-H), 5.28 (1H, dd, *J* 10.5, 1.2, 5-*H*H), 5.39 (1H, dd, *J* 17.4, 2.7, 5-H*H*), 5.85 (1H, ddd, *J* 17.4, 10.5, 6.4, 4-H); δ_C (100 MHz; $CDCl_3$) 43.4 (C-1), 53.7 (C-2), 70.2 (C-3), 117.7 (C-5), 135.5 (C-4). All data in accordance with the literature.[9]

(2*S*,3*S*)-1,2-Epoxy-3-hydroxy-pent-4-en 37[10]

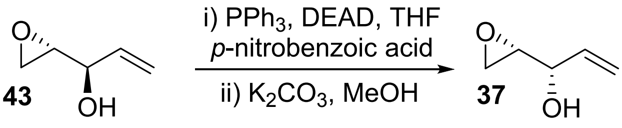

A round-bottomed flask was charged with triphenylphosphine (2.71 g, 10.35 mmol) and *para*-nitrobenzoic acid (1.72 g, 10.35 mmol) in THF (40 mL) and cooled to 0 °C. To the resulting solution, diethyl azodicarboxylate (1.63 mL, 10.35 mmol) was added dropwise and allowed to stir for 5 minutes at 0 °C. Epoxide **43** (986 mg, 9.86 mmol) in THF (10 mL) was added dropwise at 0 °C and then the reaction mixture was allowed to warm to RT with stirring over 1 h. Upon consumption of the starting material by TLC, the reaction mixture was concentrated and filtered through a plug of SiO_2 (Pet:Et_2O, 90:10 to 80:20) to obtain the *para*-nitrobenzoic acid adduct and other non-polar by-products. The filtrate was then concentrated *in vacuo* and taken up in methanol (10 mL). Potassium carbonate (1.43 g, 10.35 mmol) was added and the reaction mixture left to stir for 15 minutes, until disappearance of the *para*-nitrobenzoic acid adduct by TLC. Methanol was removed *in vacuo* and water (10 mL) and CH_2Cl_2 (10 mL) was added to the crude residue. The organic layer was separated and the aqueous layer extracted with CH_2Cl_2 (3 x 10 mL). The organic layers were combined, dried ($MgSO_4$) and concentrated *in vacuo* to give white oily residue. This was purified by column chromatography (Pentane: Et_2O, 60:40) to give alcohol **37** as a yellow oil (388 mg, 39%); $[\alpha]_D^{23}$ -12 (c 1.0, $CHCl_3$), lit.[10] *ent*-**37** $[\alpha]_D^{25}$ +20.7 (c 1.8, $CHCl_3$); δ_H (400 MHz; $CDCl_3$) 2.17 (1H, br. s., O*H*), 2.67 (1H, dd, *J* 5.0, 4.1, 1-*H*H), 2.76 (1H, dd, *J* 5.0, 2.8, 1-H*H*), 3.00 (1H, ddd, *J* 5.0, 4.1, 2.8, 2-H), 3.90 (1H, br. m, 3-H), 5.16 (1H, dd, *J* 10.4, 1.2, 5-*H*H), 5.31 (1H, dd, *J* 17.3, 2.8, 5-H*H*), 5.86 (1H, ddd, *J* 17.3, 10.4, 6.4, 4-H); δ_C (100 MHz; $CDCl_3$) 44.7 (C-1), 54.8 (C-2), 72.6 (C-3), 116.7 (C-5), 136.1 (C-4). All data in accordance with the literature.[10]

(2*S*)-1,2-Epoxy-3-*O*-(*N*,*N*-diisopropylcarbamate)-pent-4-en 39

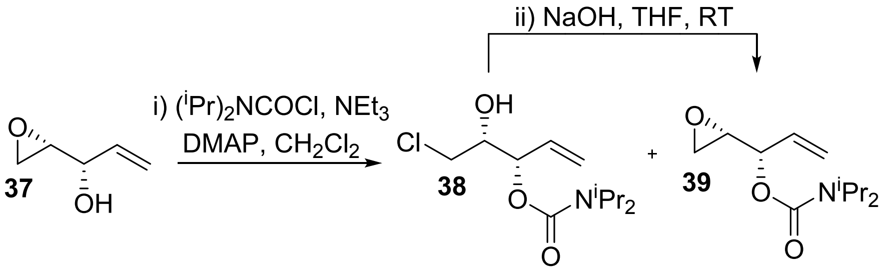

Syn-epoxide **37** (180 mg, 1.8 mmol) was added to a solution of triethylamine (275 µl, 1.98 mmol) and *N*,*N*-diisopropylcarbamoyl chloride (443 mg, 2.7 mmol) in dry CH_2Cl_2 (3 mL) and heated to reflux for 5 h under N_2. Upon disappearance of the starting epoxide by TLC, the

solvent was removed under reduced pressure to give the crude residue which was redissolved in Et_2O (10 mL) filtered through a plug of silica, washing with Et_2O (2 x 10 mL). The solvent was then removed under reduced pressure, giving a crude mixture of epoxide **39** and chlorohydrin **38**. The crude residue was redissolved in THF (10 mL) and NaOH (72 mg, 1.8 mmol) was added. The reaction was left to stir for 10 minutes at RT, until chlorohydrin **38** was converted to epoxide **39**, as monitored by TLC. The THF was removed under reduced pressure, water (10 mL) added and extracted with CH_2Cl_2 (3 x 5 mL). The combined organic phases were dried ($MgSO_4$) and concentrated *in vacuo* to afford the crude residue, which was further purified by column chromatography (Pet: Et_2O, 80:20) to give epoxide **39** as a colourless oil (282 mg, 69%); $[\alpha]_D^{23}$ -20 (*c* 1.00, $CHCl_3$); δ_H (400 MHz; $CDCl_3$) 1.21 (6H, br. s., 2 x CH_3), 1.23 (6H, br. s., 2 x CH_3), 2.67 (1H, dd, *J* 4.5, 2.7, 1-*H*H), 2.82 (1H, app. td, *J* 4.5, 1.8, 1-H*H*), 3.16 (1H, ddd, *J* 9.8, 2.9, 1.8, 2-H), 3.83 (1H, br. s., NC*H*), 4.01 (1H, br. s., NC*H*), 5.13 (1H, app. tt., *J* 5.8, 1.5, 3-H), 5.27 (1H, dt, *J* 10.7, 2.5, 5-*H*H), 5.36 (1H, dt, *J* 17.1, 1.5, 5-H*H*), 5.88 (1H, ddd, *J* 17.1, 10.7, 1.5, 4-H); δ_C (100 MHz; $CDCl_3$) 20.4 (2 x CH_3), 21.4 (2 x CH_3), 44.4 (C-1), 45.9 (2 x br. N*C*H), 52.8 (C-2), 74.6 (C-3), 117.9 (C-5), 133.1 (C-4), 154.5 (*C*O); *m/z* (ESI) 250.1416 (MNa^+, 100%, $C_{12}H_{21}O_3NNa$ requires 250.1414).

(2*S*,3*S*)-2-Hydroxy-3-*O*-(*N*,*N*-diisopropylcarbamate)-pent-4-ene 40

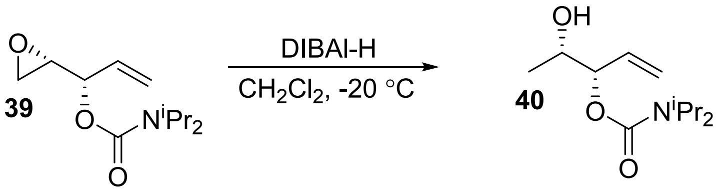

Diisobutylaluminum hydride (1.84 mL, 1 M in hexanes, 1.84 mmol) was added dropwise to a stirring solution of epoxide **39** (139 mg, 0.61 mmol) in dry CH_2Cl_2 (6 mL) at -20 °C under N_2. The reaction was left to stir for 20 minutes at -20 °C, then 1 M HCl aqueous solution was added (5 mL). This was left to stir for a further 10 minutes, until the two phases separated. The organics were separated and the aqueous layer extracted with CH_2Cl_2 (3 x 5 mL). The combined organic phases dried ($MgSO_4$) and concentrated *in vacuo* to give alcohol **40** as a yellow oil (92 mg, 66%); $[\alpha]_D^{23}$ -23 (*c* 1.00, $CHCl_3$); δ_H (400 MHz; $CDCl_3$) 1.18 (3H, d, *J* 6.4, 5-H_3), 1.22 (6H, br. s, (C*H_3*)$_2$), 1.23 (6H, br. d, (C*H_3*)$_2$), 2.30 (1H, br. s, O*H*), 3.80 (1H, br. s., NC*H*), 3.88 (1H, app. q., *J* 6.4, 4-H), 4.02 (1H, br. s., NC*H*), 5.09 (1H, t, *J* 6.1, 3-H), 5.27 (1H, d, *J* 10.0, 1.2, 1-*H*H), 5.33 (1H, dt, *J* 17.4,

1.5, 1-H*H*), 5.86 (1H, ddd, *J* 17.4, 10.0, 6.4, 2-H); δ_C (100 MHz; $CDCl_3$) 19.0 (C-5), 20.6 (2 x br. *C*H_3), 21.5 (2 x br. *C*H_3), 46.1 (2 x br. N*C*H), 69.4 (C-4), 79.5 (C-3), 118.3 (C-2), 134.0 (C-1), 155.2 (*C*O); *m/z* (ESI) 252.1578 (MNa^+, 100%, $C_{12}H_{23}NO_3NaSi$ requires 252.1570).

(1*R*,3*S*,4*S*,5*S*)-1-(Benzyldimethylsilyl)-4-*O*-(*N*,*N*-diisopropylcarbamate)-3-hydroxy-5-methyl-tetrahydropyran 41

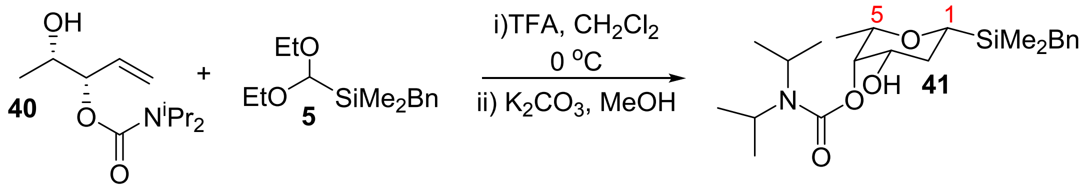

Trifluoroacetic acid (900 µl, 7.20 mmol) was added dropwise to a solution of alcohol **40** (61 mg, 0.36 mmol) and silyl acetal **5** (118 mg, 0.47 mmol) in dry CH_2Cl_2 (4 mL) at 0 °C. This was stirred for 1 h at RT, then aqueous saturated $NaHCO_3$ (15 mL) and triethylamine was added until pH >7. The organic phase was separated and the aqueous phase extracted with CH_2Cl_2 (3 x 15 mL). The combined organic phases were then concentrated *in vacuo* and the resulting crude residue was redissolved in methanol (15 mL), to which K_2CO_3 (298 mg, 2.16 mmol) was added and left to stir for 30 minutes. The methanol was removed under reduced pressure, water (20 mL) added and extracted with CH_2Cl_2 (3 x 20 mL). The combined organic phases were washed with brine (10 mL), dried ($MgSO_4$) and concentrated *in vacuo*. Which was purified by column chromatography (Pet:Et_2O, 75:25) to yield **41** as a yellow oil (105 mg, 72%); $[\alpha]_D^{23}$ +14 (*c* 1.00, $CHCl_3$); δ_H (400 MHz; $CDCl_3$) -0.04 (3H, s, SiC*H₃*), 0.03 (3H, s, SiC*H₃*), 1.22 (3H, d, *J* 6.4, 5-H_3), 1.24 (6H, br. s., (C*H₃*)₂), 1.32 (6H, br. s., (C*H₃*)₂), 1.66 (1H, td, *J* 12.2, 2.2, 2-H_{eq}), 1.74 (1H, app. q., *J* 12.2, 2-H_{ax}), 2.12 (1H, d, *J* 13.7, SiC*H*H), 2.24 (1H, d, *J* 13.7, SiCH*H*), 3.10 (1H, dd, *J* 12.2, 2.2, 1-H_{ax}), 3.46 (1H, qd, *J* 6.4, 1.2, 5-H_{ax}), 3.85 (2H, m, 3-H and NC*H*), 4.12 (1H, br. s., NC*H*), 4.96 (1H, dd, *J* 2.9, 1.2, 4-H), 7.00-7.10 (3H, m, ArH), 7.20-7.23 (2H, m, ArH); δ_C (100 MHz; $CDCl_3$) -6.2 (Si*C*H_3), -6.1 (Si*C*H_3), 17.9 (C-5), 20.4 (2 x *C*H_3), 21.6 (2 x *C*H_3), 22.9 (Si*C*H_2), 30.4 (C-2), 45.7 (N*C*H), 46.8 (N*C*H), 68.3 (C-

1), 71.0 (C-3), 74.4 (C-4), 75.8 (C-5), 124.1 (C-Ar), 128.1 (2 x C-Ar), 128.2 (2 x C-Ar), 139.7 (C-Ar), 157.2 (*C*O); *m/z* (ESI) 430.2392 (MNa^+, 100%, $C_{22}H_{37}O_4NNaSi$ requires 430.2384).

(3*S*,4*S*,5*S*)-1,3-*O*-Acetyl-4-*O*-(*N*,*N*-diisopropylcarbamate)5-methyl-tetrahydropyran 42

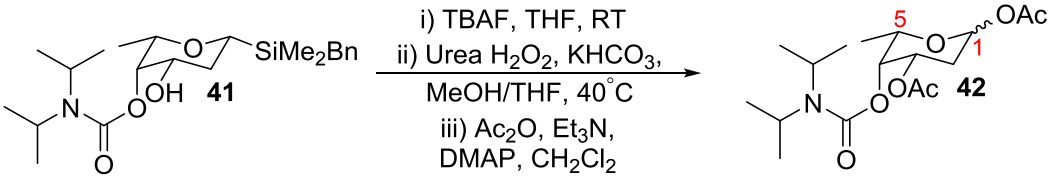

TBAF (1.2 mL, 0.60 mmol, 0.5 M solution in THF) was added dropwise over 15 minutes to a solution of 1-silyl tetrahydropyran **41** (80 mg, 0.20 mmol, 1 eq) in dry THF (2 mL) at 0 °C under N_2. The reaction was warmed slowly to 30 °C and allowed to stir for 1 h until disappearance of 1-silyl tetrahydropyran **41** by TLC. Urea hydrogen peroxide (92 mg, 0.98 mmol), potassium hydrogen carbonate (60 mg, 0.6 mmol) and dry methanol (0.6 mL) was added. This was left to stir at RT for 12 h and upon completion aqueous saturated sodium thiosulphate solution (2 mL) was added, the organic phases separated and aqueous layer extracted with CH_2Cl_2 (3 x 5 mL). The crude reaction mixture was concentrated *in vacuo* then taken up in dry CH_2Cl_2 (3 mL). The solution was then cooled to 0 °C and triethylamine (420 µl, 3.00 mmol), a crystal of DMAP then acetic anhydride (190 µl, 2.00 mmol) was added. The reaction was warmed to RT and left to stir for 1 h and on completion water (6 mL) was added and the organic phase separated. The aqueous phase was washed with CH_2Cl_2 (3 x 10 mL), the combined organic phases dried ($MgSO_4$) and concentrated *in vacuo*. The residue was then purified by column chromatography (Pet:Et_2O, 60:40) to give **42** as a yellow oil, as a mixture of α/β anomers (54 mg, 77%, α:β 50:50); ν_{max} (neat)/cm^{-1} 2969 (CH), 1748 (C=O *acetate*), 1690 (C=O *carbamate*), 1038 (C-O); δ_H (400 MHz; $CDCl_3$) 1.15 (1H, d, *J* 6.4, 6-H_3), 1.21 (1H, d, *J* 6.4, 6-H_3), 1.22-1.31 (24H, br. s., 4 x (CH_3)$_2$), 1.90

(4H, m, 4-H_2), 1.95 (2H, m, 2-*H*H), 2.00-2.01 (6H, br. s., 2 x 3-C(O)C*H_3*), 2.10 (3H, s, β 1-C(O)C*H_3*), 2.13 (3H, s, α 1-C(O)C*H_3*), 2.16 (1H, m, 2 x 2-H*H*), 3.82 (1H, qd, *J* 6.4, 1.4, 5-H_{ax}), 3.86-4.07 (4H, br. s., 4 x NC*H*), 4.17 (1H, qd, *J* 6.4, 1.2, 5-H_{ax}), 5.03 (1H, ddd, *J* 11.7, 5.5, 3.4, 3-H_{ax}), 5.13 (1H, dd, *J* 3.4, 1.4, 4-H_{eq}), 5.23 (1H, dd, *J* 2.7, 1.2, 4-H_{eq}), 5.29 (1H, ddd, *J* 12.4, 5.3, 3.2, 3-H_{ax}), 5.75 (1H, dd, *J* 9.4, 3.2, β 1-H), 6.30 (1H, dd, *J* 3.4, 0.4, α 1-H); δ_C (100 MHz; $CDCl_3$) 16.5 (C-6), 16.7 (C-6), 20.5 (4 x br. CH_3), 20.9 (3-C(O)*C*H_3), 21.1 (3-C(O)O*C*H_3), 21.4 (4 x br. *C*H_3), 29.4 (2-C), 31.1 (2-C), 45.8 (2 x br. N*C*H), 46.4 (2 x br. N*C*H), 66.4 (C-3), 67.9 (C-4), 68.2 (C-5), 68.8 (C-3), 69.2 (C-4), 71.0 (C-5), 91.9 (α C-1), 92.0 (β C-1), 154.7 (*CO carbamate*), 158.8 (*CO carbamate*), 169.0 (*CO acetate*), 169.5 (*CO acetate*), 169.9 (*CO acetate*), 170.2 (*CO acetate*); *m/z* (ESI) 382.1847 (MNa^+, 100%, $C_{17}H_{29}O_7NNa$ requires 382.1836).

(2*S*,3*R*)-1,2-Epoxy-3-*O*-(*N*,*N*-diisopropylcarbamate)-pent-4-ene SI-6

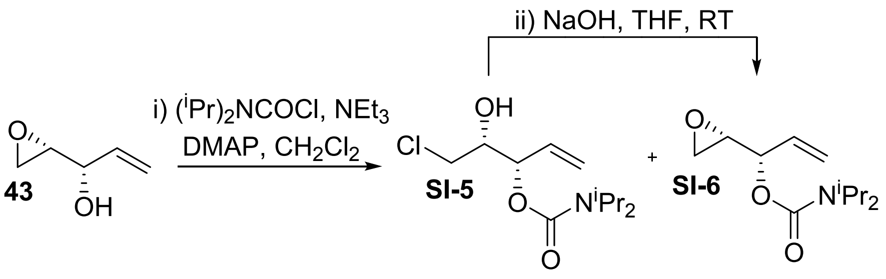

Anti-epoxide **44** (120 mg, 1.20 mmol) was added to a solution of triethylamine (183 µl, 1.32 mmol) and *N*,*N*-diisopropylcarbamoyl chloride (590 mg, 3.60 mmol) in dry CH_2Cl_2 (6 ml) and heated to reflux for 5 h. Upon disappearance of the epoxide solvent was removed under reduced pressure to yield the crude residue which was redissolved in Et_2O (10 ml) then passed through a plug of Celite®, washing with Et_2O (2 x 10 ml). The solvent was then removed under reduced pressure, giving a crude mixture of the protected epoxide compound **SI-6** and chlorohydrin **SI-5**. The reaction was left to stir for 10 minutes at room temperature, until chlorohydrin **SI-5** was converted to the title compound **SI-6**, as monitored by TLC. The THF was removed under reduced pressure, water (10 ml) added and extracted with CH_2Cl_2 (3 x 5 ml). The combined organic phases were dried ($MgSO_4$) and concentrated *in vacuo* to afford the crude residue, which was further purified by column chromatography (Pet: Et_2O, 80:20) to afford epoxide **SI-6** as a colourless oil (161 mg, 59%); $[\alpha]_D^{23}$ -32 (*c* 1.00, $CHCl_3$); δ_H (400 MHz; $CDCl_3$) 1.22 (6H, br. s, 2 x CH_3), 1.24 (6H, br. s, 2 x CH_3), 2.70 (1H, dd, *J* 5.1, 2.7, 1-*H*H), 2.80 (1H, dd, *J* 5.1, 4.2, 1-H*H*), 3.16 (1H, ddd, *J* 6.8, 4.2, 2.7, 2-H), 3.81 (1H, br. s, NC*H*), 4.02 (1H, br. s, NC*H*),

5.25 (1H, dd, *J* 6.8, 4.4, 3-H), 5.30 (1H, dd, *J* 10.6, 2.4, 5-*H*H), 5.39 (1H, dd, *J* 17.2, 2.4, 5-H*H*), 5.84 (1H, ddd, *J* 17.2, 10.6, 4.4, 4-H); δ_C (100 MHz; $CDCl_3$) 20.6 (2 x CH_3), 21.3 (2 x CH_3), 44.8 (C-1), 45.4 (br. N*C*H), 46.1 (br. N*C*H), 52.4 (C-2), 74.2 (C-3), 119.1 (C-5), 132.5 (C-4), 154.5 (CO); *m/z* (ESI) 250.1408 (MNa^+, 100%, $C_{12}H_{21}O_3NNa$ requires 250.1414).; Data for chlorohydrin **SI-5**; $[\alpha]_D^{23}$ -23.5 (*c* 1.00, $CHCl_3$); δ_H (400 MHz; $CDCl_3$) 1.19-1.23 (6H, br. s, 2 x CH_3), 1.24-1.27 (6H, br. s, 2 x CH_3), 3.18 (1H, br. s, OH), 3.53 (1H, dd, *J* 11.3, 7.6, 1-*H*H), 2.80 (1H, dd., *J* 11.3, 3.9, 1-H*H*), 3.83 (1H, br. s., NC*H*), 4.01 (1H, br. s., 2-H), 4.07 (1H, br. s., NC*H*), 5.34 (1H, dd, *J* 10.7, 2.3, 5-*H*H), 5.35 (1H, m, 3-H), 5.39 (1H, d, *J* 17.4, 2.3, 5-H*H*), 5.95 (1H, ddd, *J* 17.4, 10.7, 6.9, 4-H); δ_C (100 MHz; $CDCl_3$) 20.2 (2 x br. *C*H_3), 21.5 (2 x br. *C*H_3), 45.8 (C-1), 45.4 (N*C*H), 46.1 (N*C*H), 73.6 (C-2), 76.8 (C-3), 119.0 (C-5), 132.9 (C-4), 154.9 (*C*O); *m/z* (ESI) 286.1194 (MNa^+, 100%, $C_{12}H_{22}O_3NClNa$ requires 286.1180).

(2*S*,3*R*)-4-hydroxy-3-*O*-(*N*,*N*-diisopropylcarbamate)-pent-1-ene 44

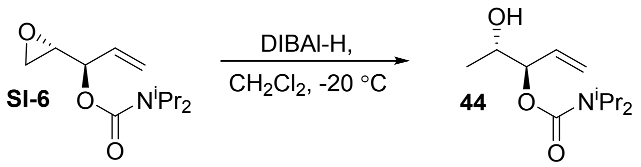

Diisobutylaluminum hydride (2.25 ml, 1 M in hexanes, 2.25 mmol) was added dropwise to a stirring solution of epoxide **SI-6** (170 mg, 0.75 mmol) in dry CH_2Cl_2 (8 ml) at -20 °C. The reaction was left to stir for 20 minutes at -20 °C, then 1 M HCl aqueous solution was added (5 ml). This was left to stir for 10 minutes, until the two phases separated. The organics were separated and the aqueous layer extracted with CH_2Cl_2 (3 x 5 ml). The combined organic phases dried ($MgSO_4$) and concentrated *in vacuo* to afford alcohol **44** as a yellow oil (148 mg, 87%); $[\alpha]_D^{23}$ -40 (*c* 1.00, $CHCl_3$); δ_H (400 MHz; $CDCl_3$) 1.17 (3H, d, *J* 6.4, 1-H_3), 1.23 (12H, br. d, 2 x $(CH_3)_2$), 3.84 (1H, br. s, NC*H*), 3.95 (1H, ddt, *J* 6.4, 3.4, 1.2, 2-H), 4.08 (1H, br. s, NC*H*), 5.21 (1H, dd, *J* 6.6, 1.2, 3-H), 5.29 (1H, dt, *J* 10.6, 1.4, 5-*H*H), 5.34 (1H, dt, *J* 17.4, 1.4, 5-H*H*), 5.88 (1H, ddd, *J* 17.4, 10.6, 6.6, 4-H); δ_C (100 MHz; $CDCl_3$) 17.8 (C-1), 20.4 (2 x br. CH_3), 21.4 (2 x br. CH_3), 46.2 (2 x br. N*C*H), 69.7 (C-2), 79.6 (C-3), 118.4 (C-4), 133.3 (C-5), 155.4 (*C*O); *m/z* (ESI) 250.1416 (MNa^+, 100%, $C_{12}H_{21}O_3NNa$ requires 250.1414).

(1*R*,3*S*,4*R*,5*S*)-1-(Benzyl(dimethyl)silane)-3,4-dihydroxy-5-methyl-tetrahydropyran 45

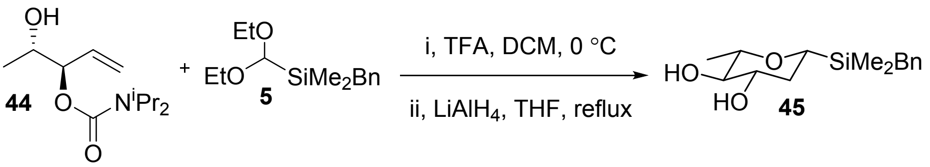

Trifluoroacetic acid (662 µl, 6.80 mmol) was added dropwise to a solution of alcohol **44** (77 mg, 0.34 mmol) and silyl acetal **5** (127 mg, 0.50 mmol) in dry CH_2Cl_2 (4 mL) at 0 °C. This was stirred for 1 h at RT, then aqueous saturated $NaHCO_3$ (15 mL) and triethylamine was added until pH >7. The organic phase was separated and the aqueous phase extracted with CH_2Cl_2 (3 x 10 mL). The combined organic phases were then concentrated *in vacuo* and the resulting crude residue was redissolved in methanol (10 ml), to which K_2CO_3 (139 mg, 1.01 mmol) was added and left to stir for 30 minutes. The methanol was removed under reduced pressure, water (20 mL) added and extracted with CH_2Cl_2 (3 x 20 mL). The combined organic phases were washed with brine (10 mL), dried ($MgSO_4$) and concentrated *in vacuo* to afford the crude residue. This residue was taken up in dry THF (6 mL) and cooled to 0 °C under a N_2 atmosphere. Lithium aluminium hydride (51 mg, 1.34 mmol) was added portion-wise and the mixture stirred for 5 minutes. The reaction mixture was then heated to reflux for 1 h, cooled to 0 °C and water (5 ml) carefully added dropwise. Ethyl acetate (5 ml) was added, the organics were separated and the aqueous layer further extracted using ethyl acetate (3 x 5 ml). The combined organics were combined, dried ($MgSO_4$) and concentrated *in vacuo* to afford the crude residue. This residue was further purified by column chromatography (Pet: Et_2O, 50:50) to afford the diol **45** as a yellow oil (54 mg, 57%); $[\alpha]_D^{23}$ -15 (*c* 0.9, $CHCl_3$); δ_H (400 MHz; $CDCl_3$) -0.03 (3H, s, SiCH_3), 0.03 (3H, s, SiCH_3), 1.29 (3H, d, *J* 6.0, 1'-H_3), 1.53 (1H, app. q, *J* 12.8, 2-H_{ax}), 1.85 (1H, ddd, *J* 12.8, 5.1, 1.8, 2-H_{eq}), 2.12 (1H, d, *J* 13.7, SiC*H*H), 2.22 (1H, d, *J* 13.7, SiCH*H*), 2.26 (1H, br. s, O*H*), 3.04 (1H, m, 4-H or 5-H), 3.07 (1H, dd, *J* 12.8, 1.8, 1-H), 3.13 (1H, m, 4-H or 5-H), 3.54 (1H, ddd, *J* 12.8, 12.2, 5.1, 3-H), 6.99-7.24 (5H, m, ArH); δ_C (100 MHz; $CDCl_3$) -6.1 (Si*C*H_3), -5.8 (Si*C*H_3), 18.2 (C-1'), 23.0 (Si*C*H_2), 34.8 (C-2), 68.1 (C-1), 74.3 (C-3), 78.3 (C-4 or C-5), 78.7 (C-4 or C-5), 124.1 (C-Ar), 128.1 (C-Ar), 128.2 (C-Ar), 139.6 (C-Ar); *m/z* (ESI) 303.1377 (MNa^+, 100%, $C_{15}H_{24}O_3SiNa$ requires 303.1387).

Chiral SFC data

(Chiralpak IA, 125 bar, 40 C, 2 mL/min, MeOH); t_R 6.08min (minor enantiomer), 6.66 min (major enantiomer); *er* = 97.5:2.5

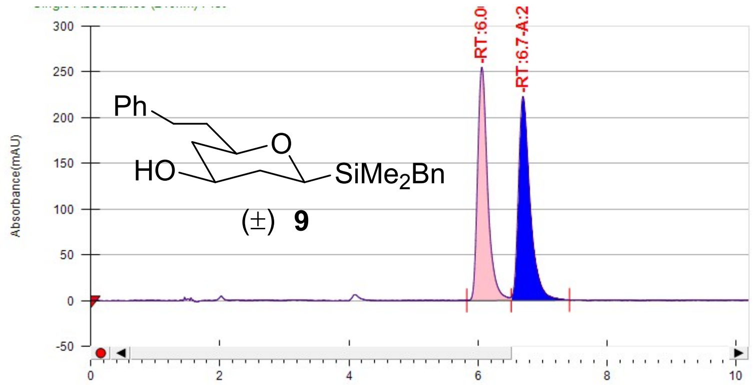

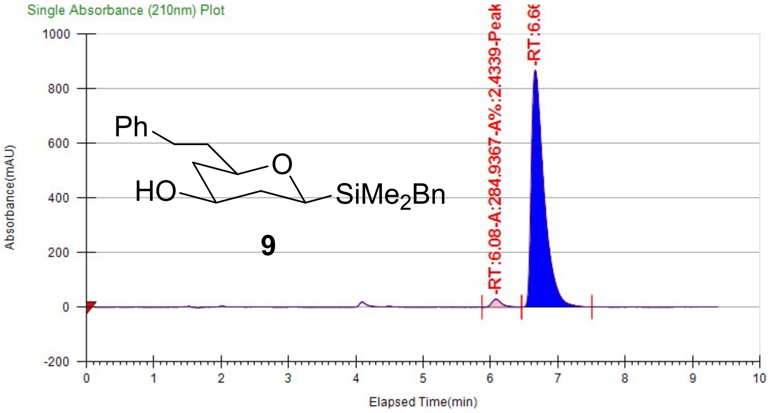

Peak Information

Peak No	% Area	Area	Ret. Time	Height	Cap. Factor
1	2.4339	284.9367	6.08 min	29.0525	6078.8333
2	97.5661	11422.072<br>1	6.66 min	867.3455	6662.1333

NMR Spectra

Benzyl(1,3-dithiane)dimethylsilane 4

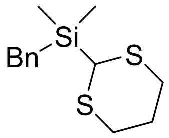

^1H NMR ($CDCl_3$, 400 MHz)

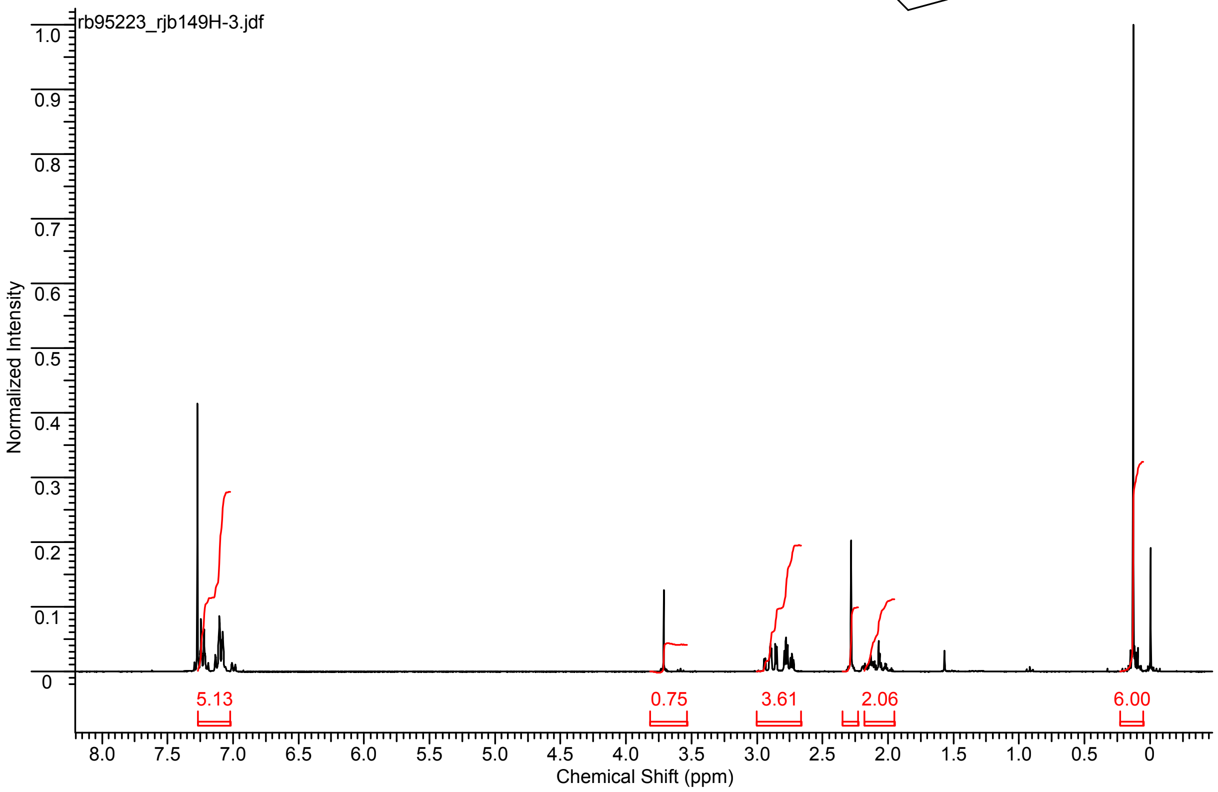

^{13}C NMR ($CDCl_3$, 100 MHz)

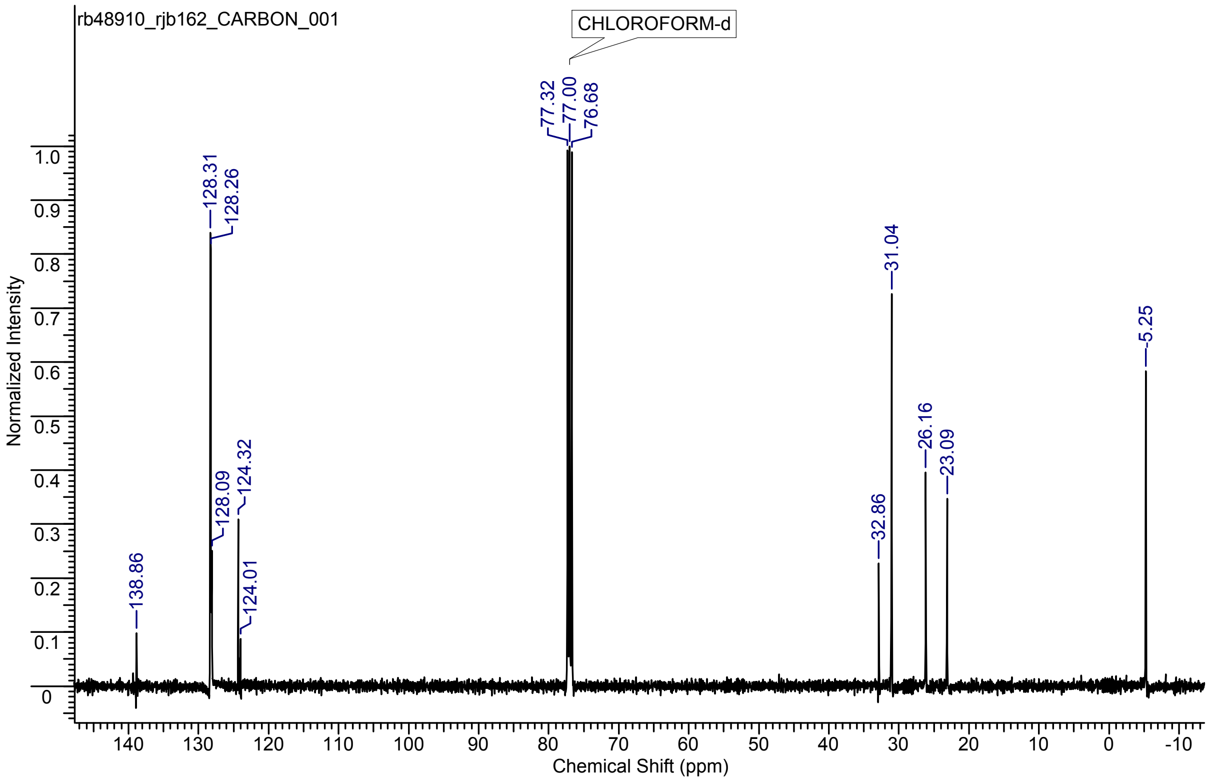

Benzyl(diethoxymethyl)dimethylsilane 5

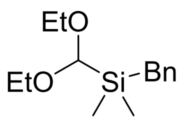

^{1}H NMR ($CDCl_3$, 400 MHz)

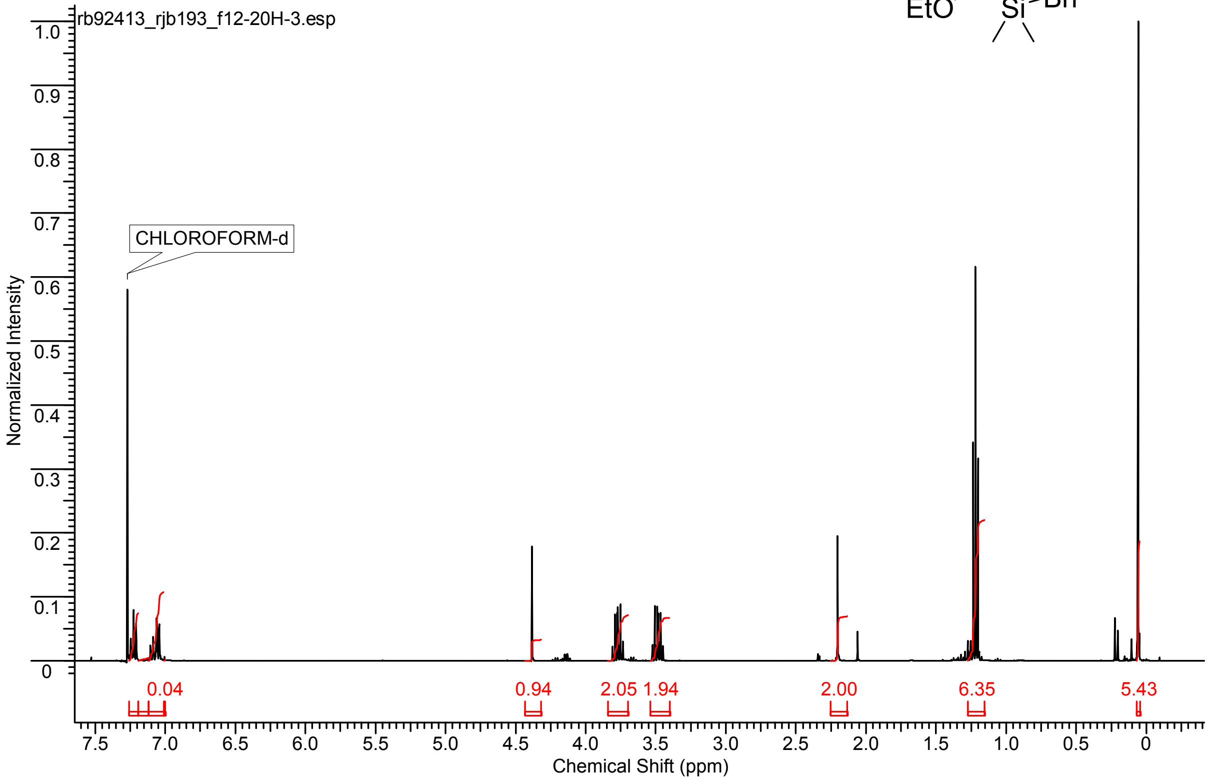

^{13}C NMR ($CDCl_3$, 100 MHz)

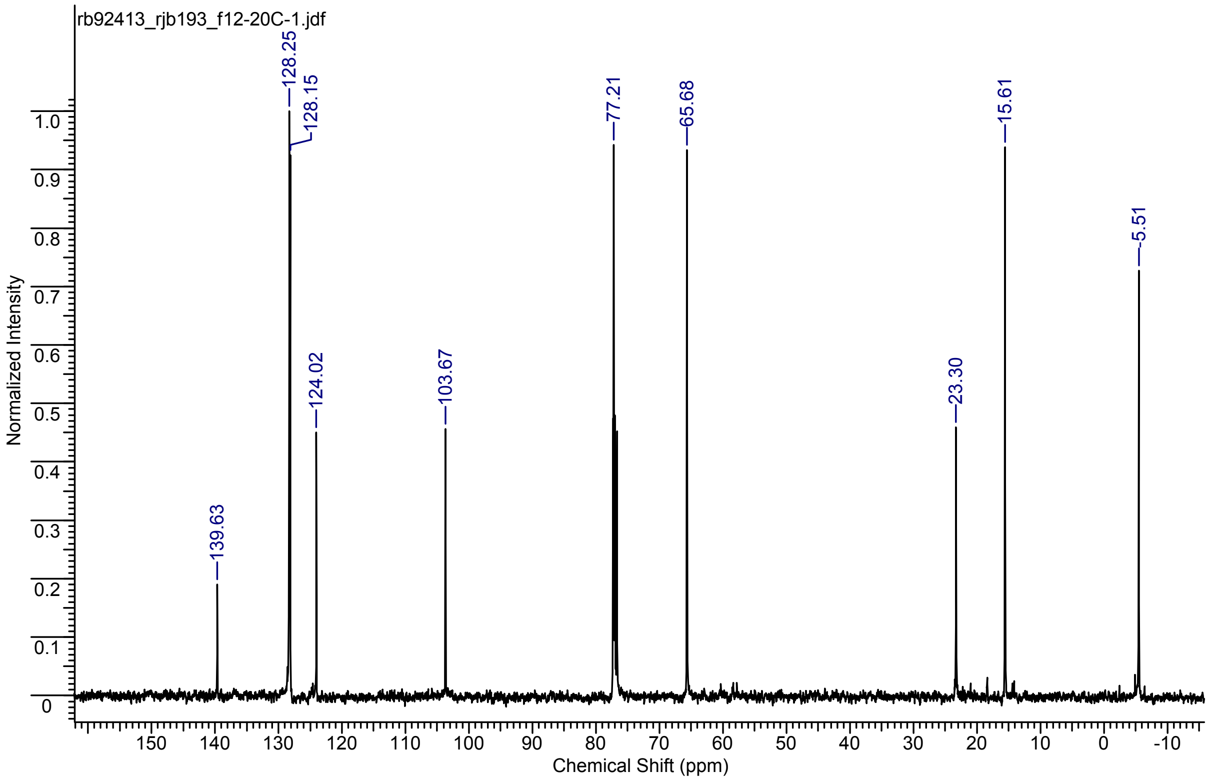

(1*S*,2*R*,3*S*,5*R*)-1-(Dimethyl(benzyl)silyl)-2-methyl-3-hydroxy-5-(2'-phenylethyl)-tetrahydropyran 7

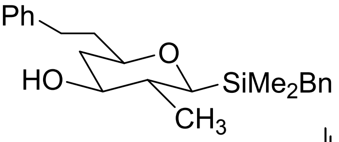

^{1}H NMR ($CDCl_3$, 400 MHz)

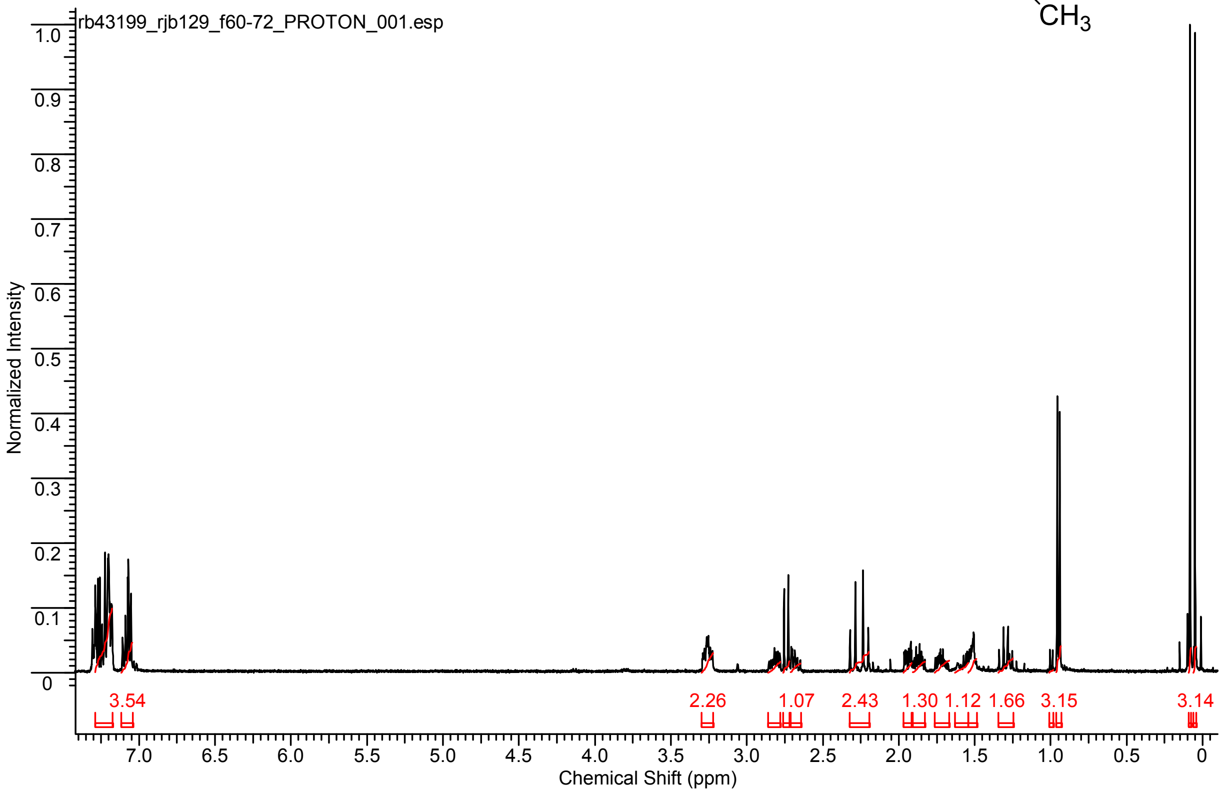

^{13}C NMR ($CDCl_3$, 100 MHz)

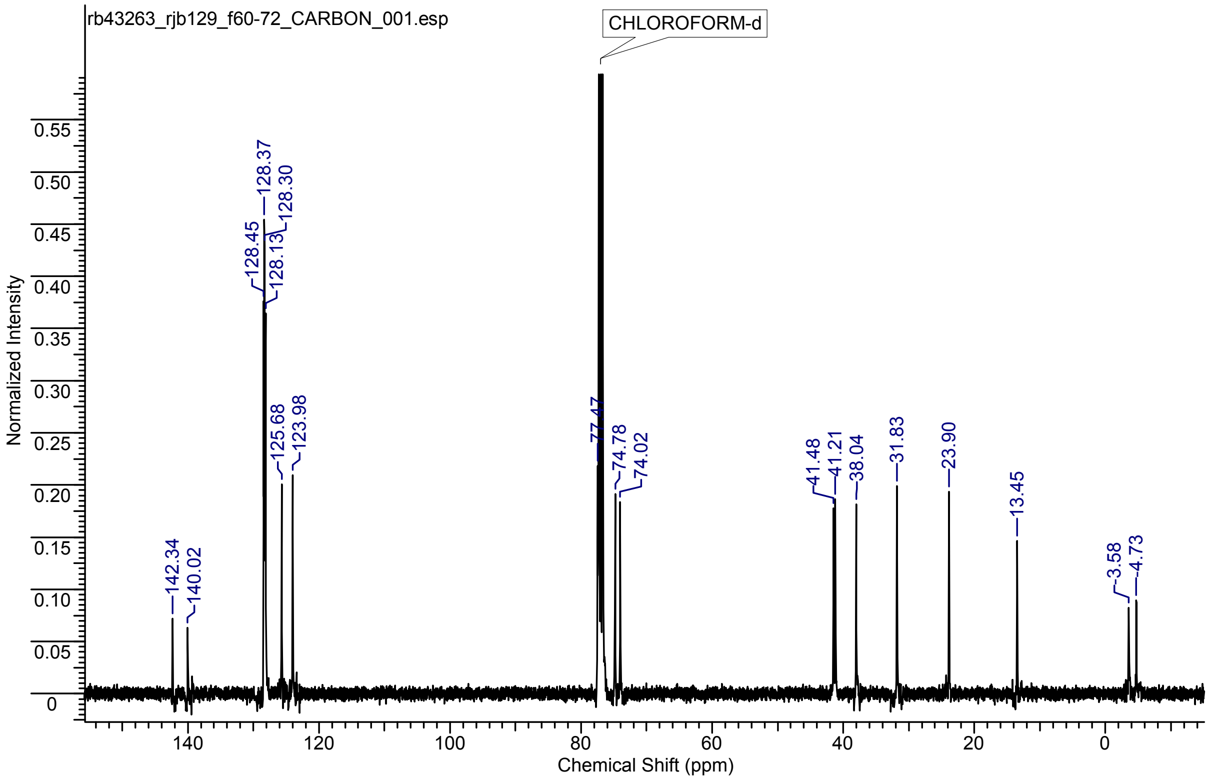

(1*S*,3*S*,5*R*)-1-(Dimethyl(benzyl)silyl)-3-hydroxy-5-(2'-phenylethyl)-tetrahydropyran 9

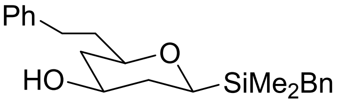

^{1}H NMR ($CDCl_3$, 400 MHz)

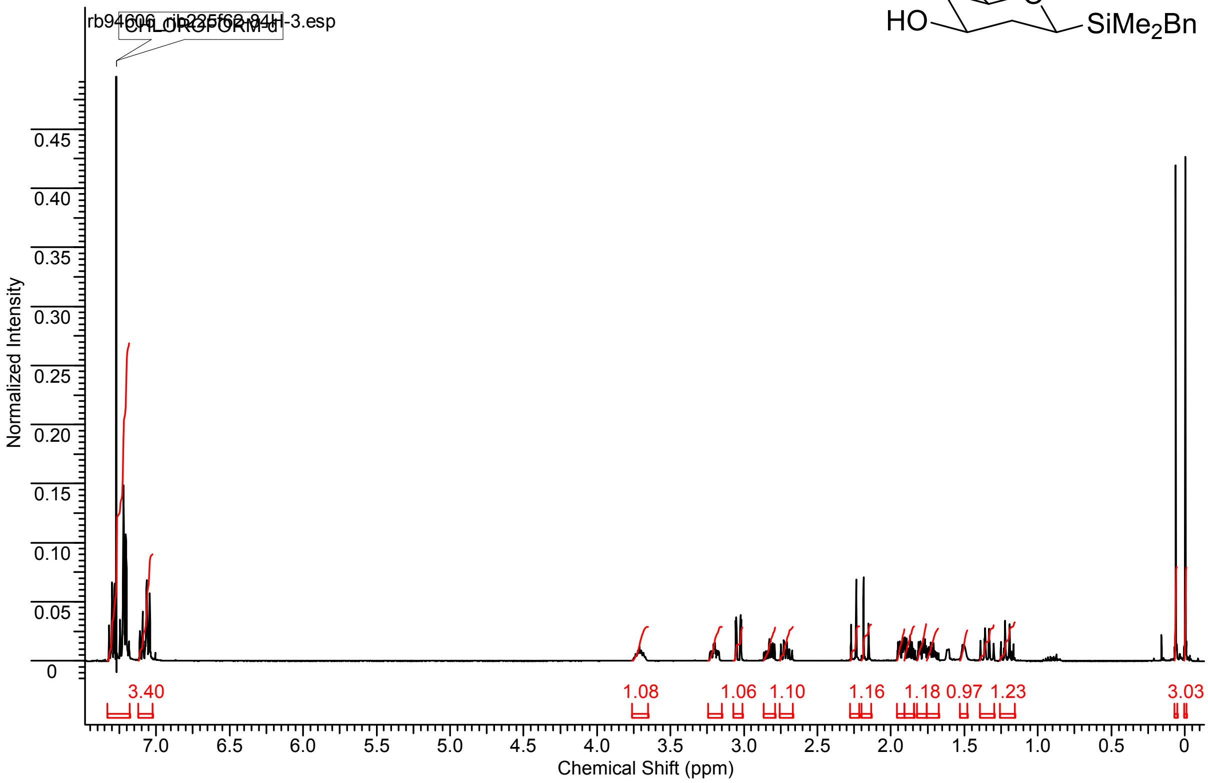

^{13}C NMR ($CDCl_3$, 100 MHz)

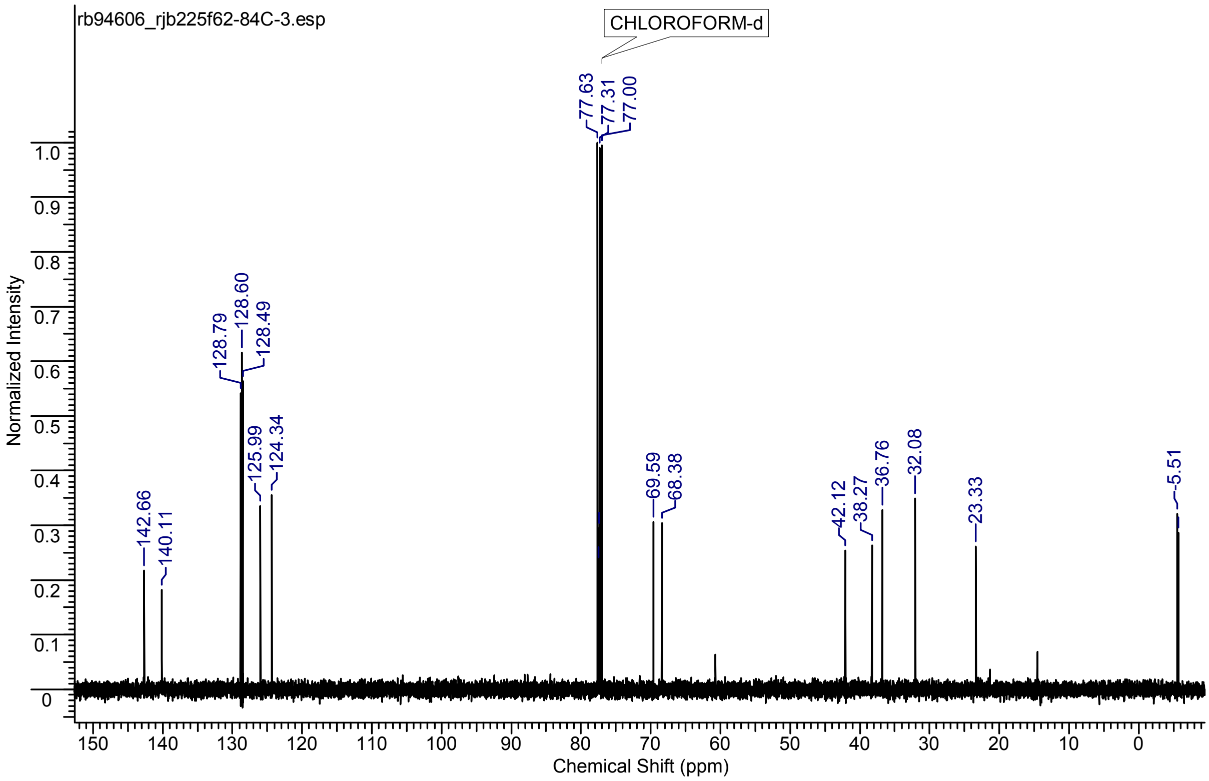

1-(Benzyl(dimethyl)silyl)-but-3-en-1-ol 10

^{1}H NMR ($CDCl_3$, 400 MHz)

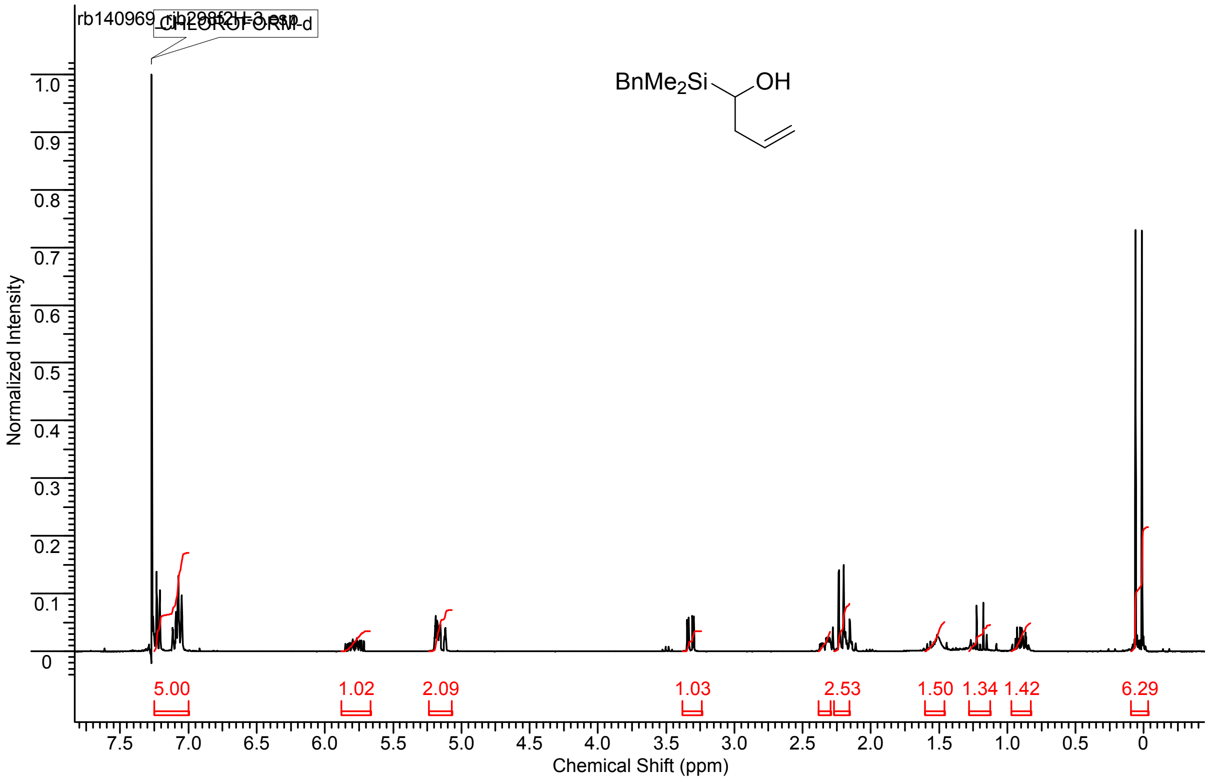

^{13}C NMR ($CDCl_3$, 100 MHz)

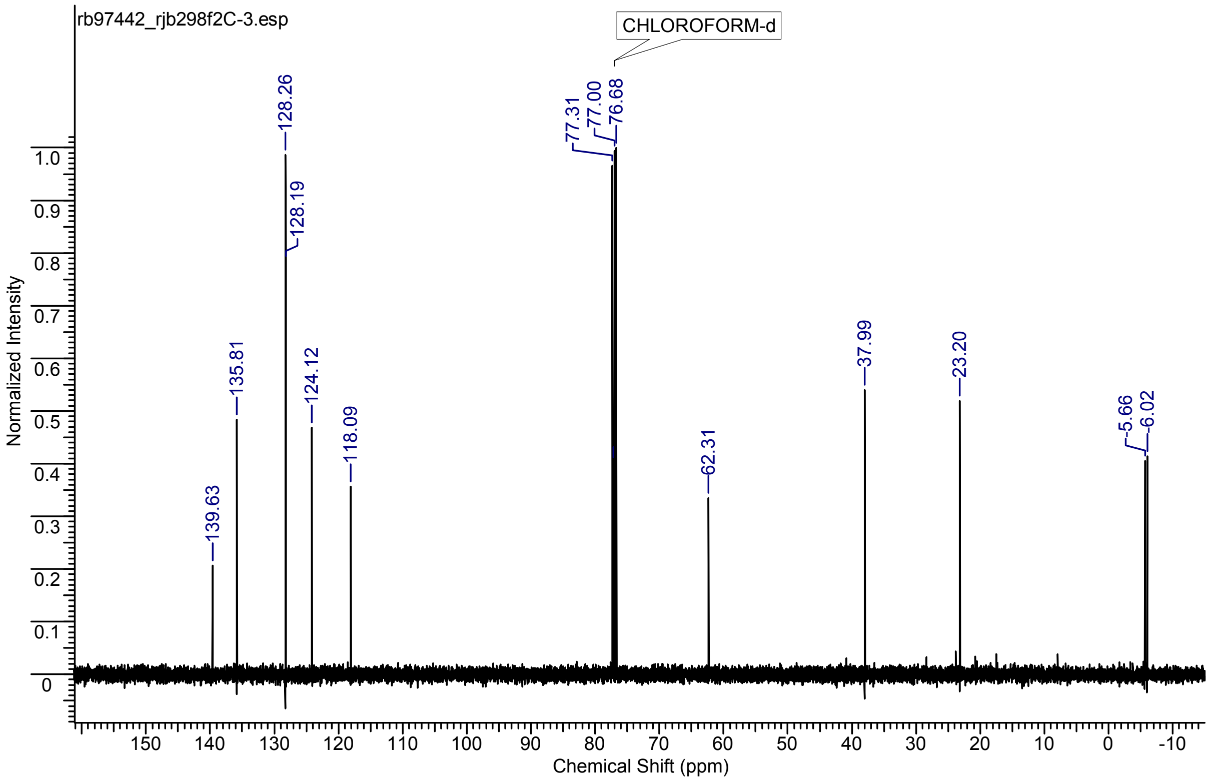

(1R*,3R*,5S*)-1-(Benzyl(dimethyl)silyl)-3-hydroxy-5-methyl-tetrahydropyran 11

^{1}H NMR ($CDCl_3$, 400 MHz)

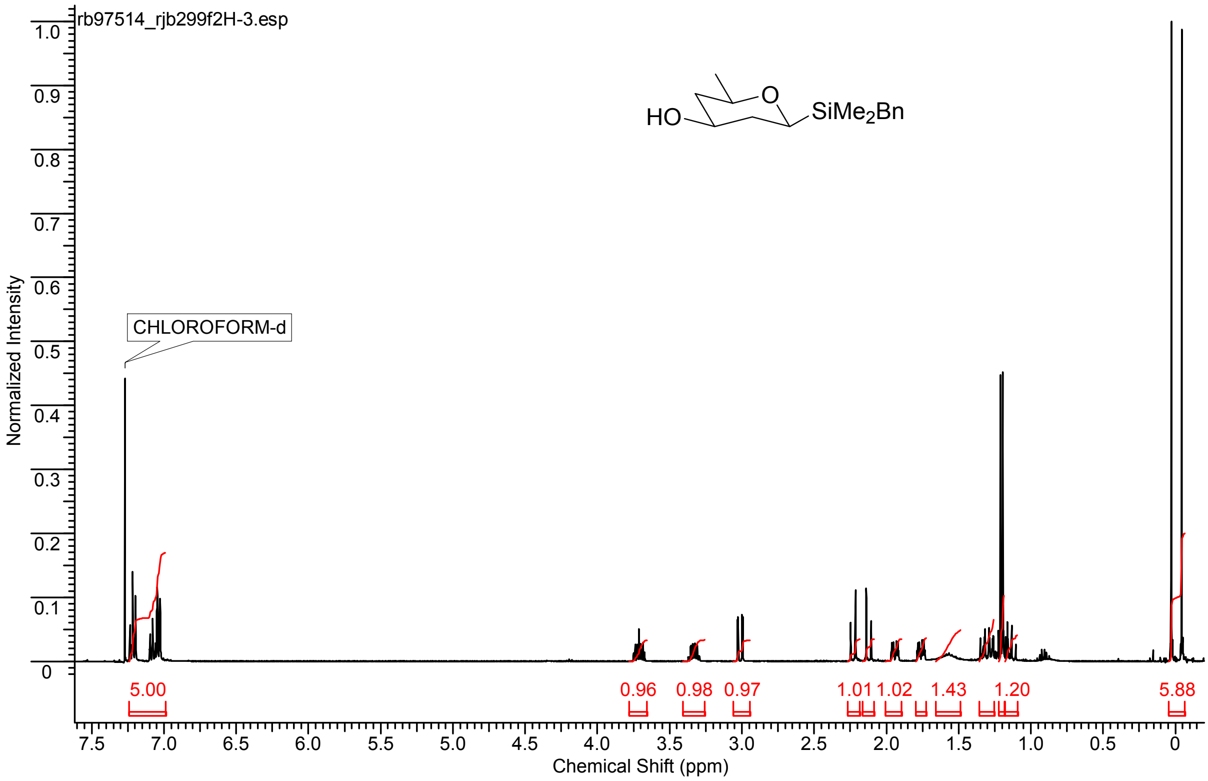

^{13}C NMR ($CDCl_3$, 100 MHz)

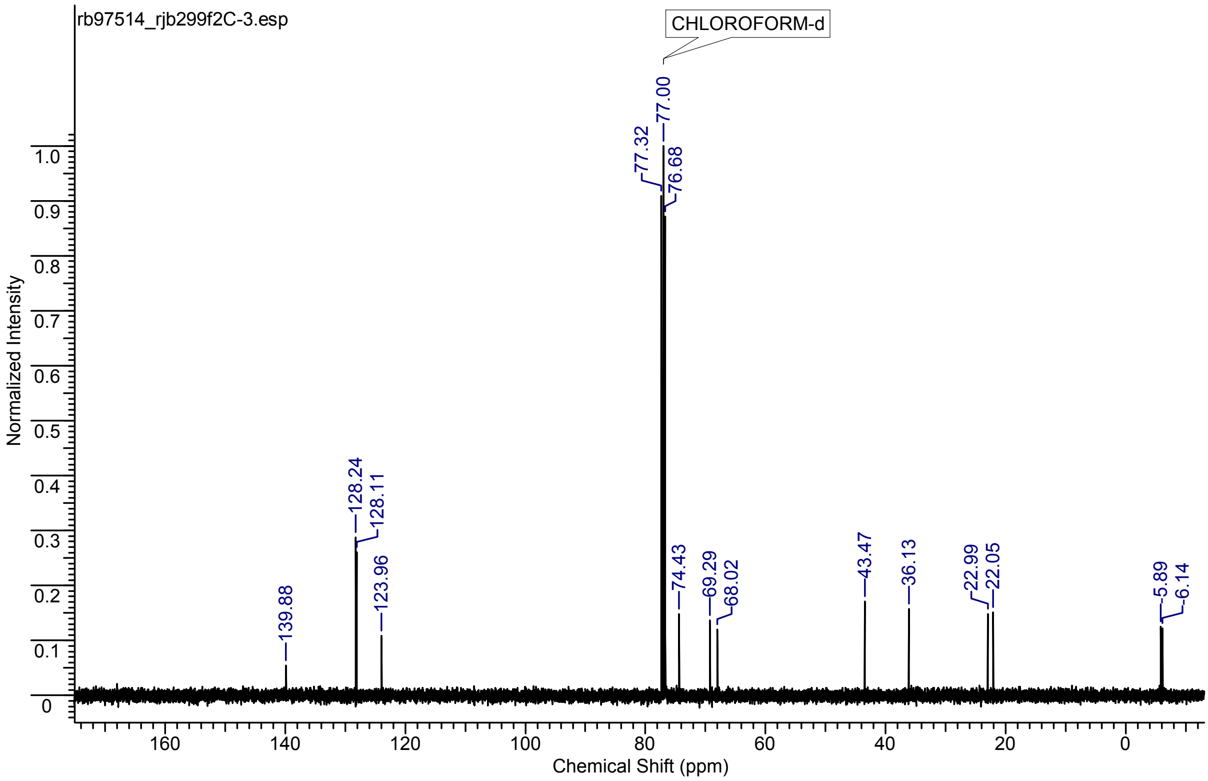

(1S*,3S*,5R*)-1-(Benzyl(dimethyl)silyl)-3-hydroxy-5-(2'-(benzyloxy)ethyl)-tetrahydropyran 12

^{1}H NMR ($CDCl_3$, 400 MHz)

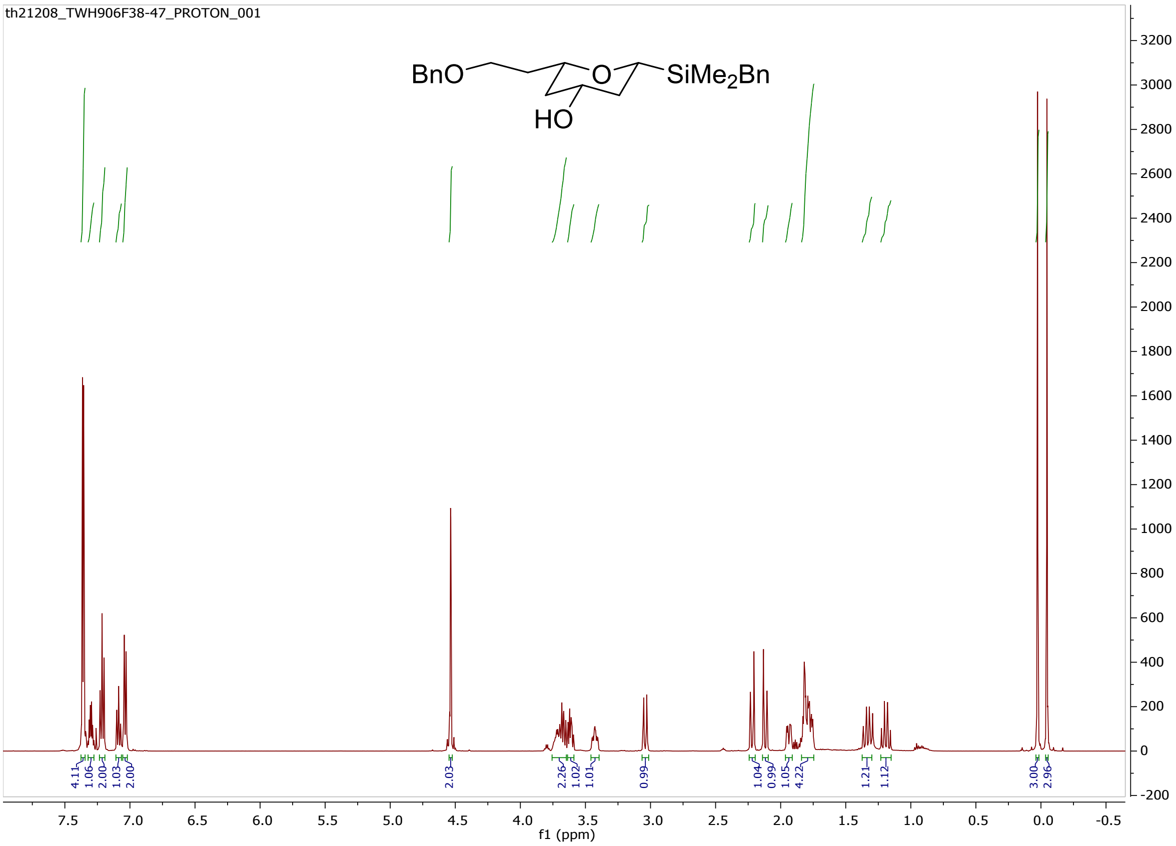

^{13}C NMR ($CDCl_3$, 100 MHz)

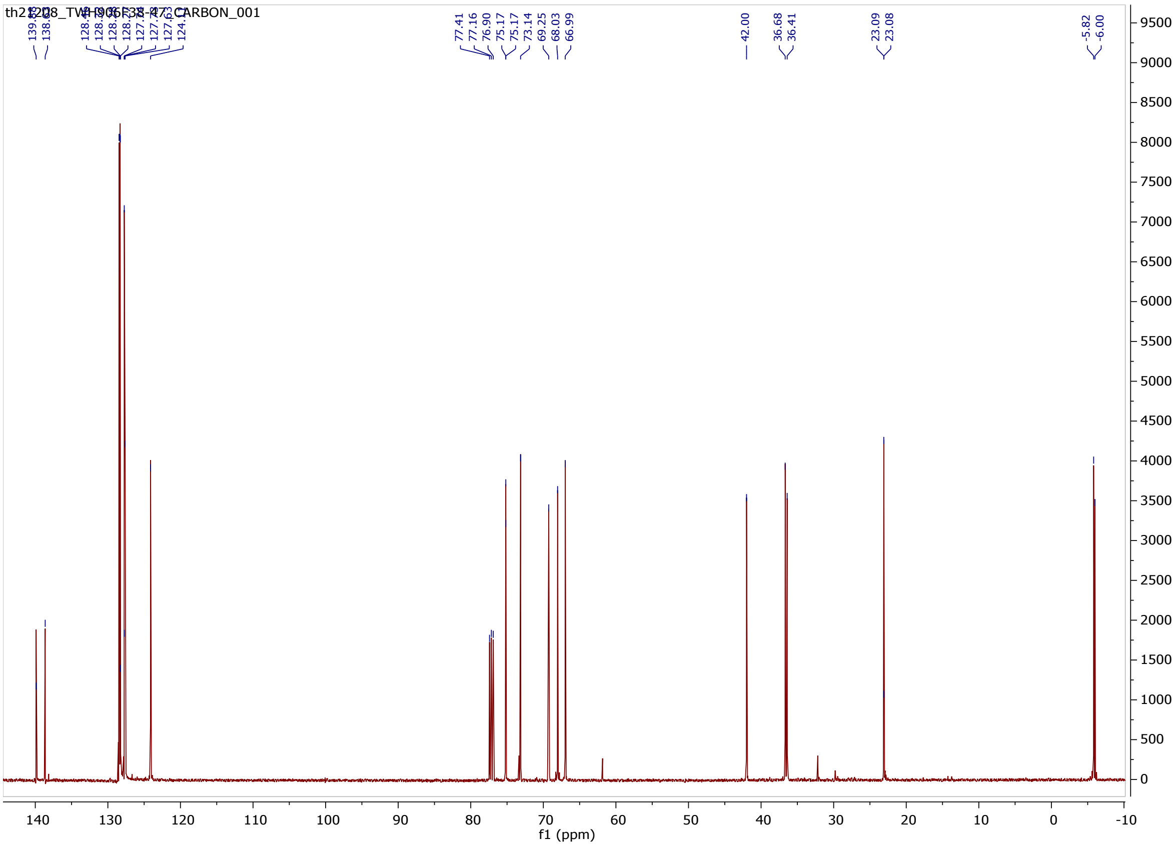

(1S*,3S*,5R*)-3-*O*-Acetyl-1-(benzyldimethylsilyl)-5-methyl-tetrahydropyran 13

^{1}H NMR ($CDCl_3$, 400 MHz)

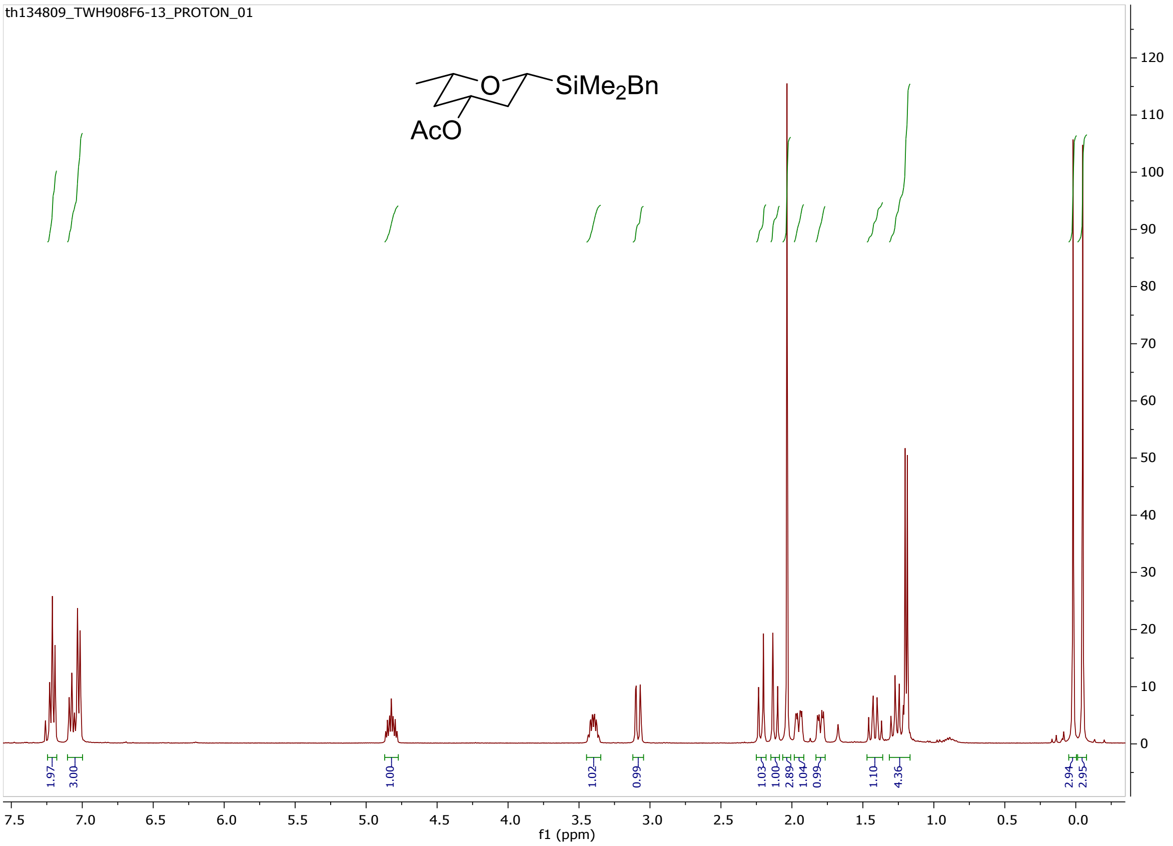

^{13}C NMR ($CDCl_3$, 100 MHz)

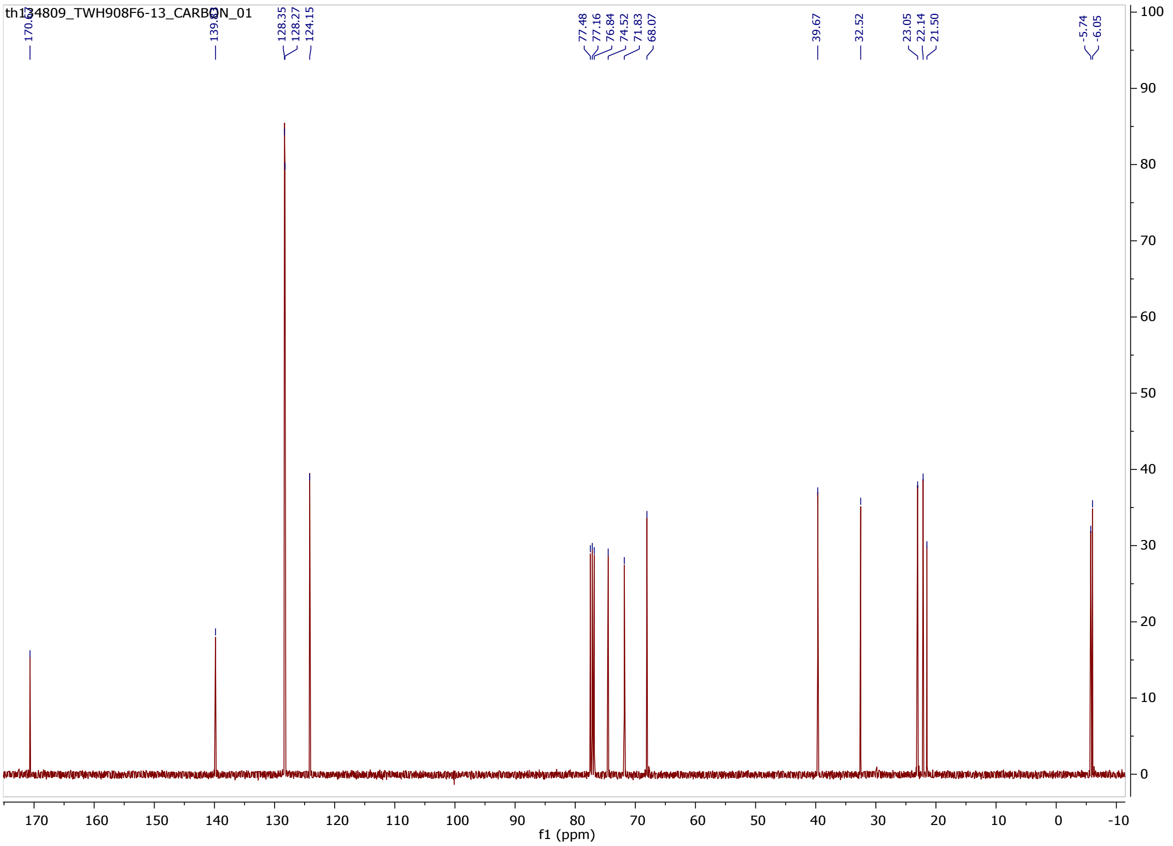

(1*S*,3*S*,5*S*)-3-*O*-Acetyl-1-(benzyldimethylsilyl)-5-(2'-(benzyloxy)ethyl)-tetrahydropyran 14

^{1}H NMR ($CDCl_3$, 400 MHz)

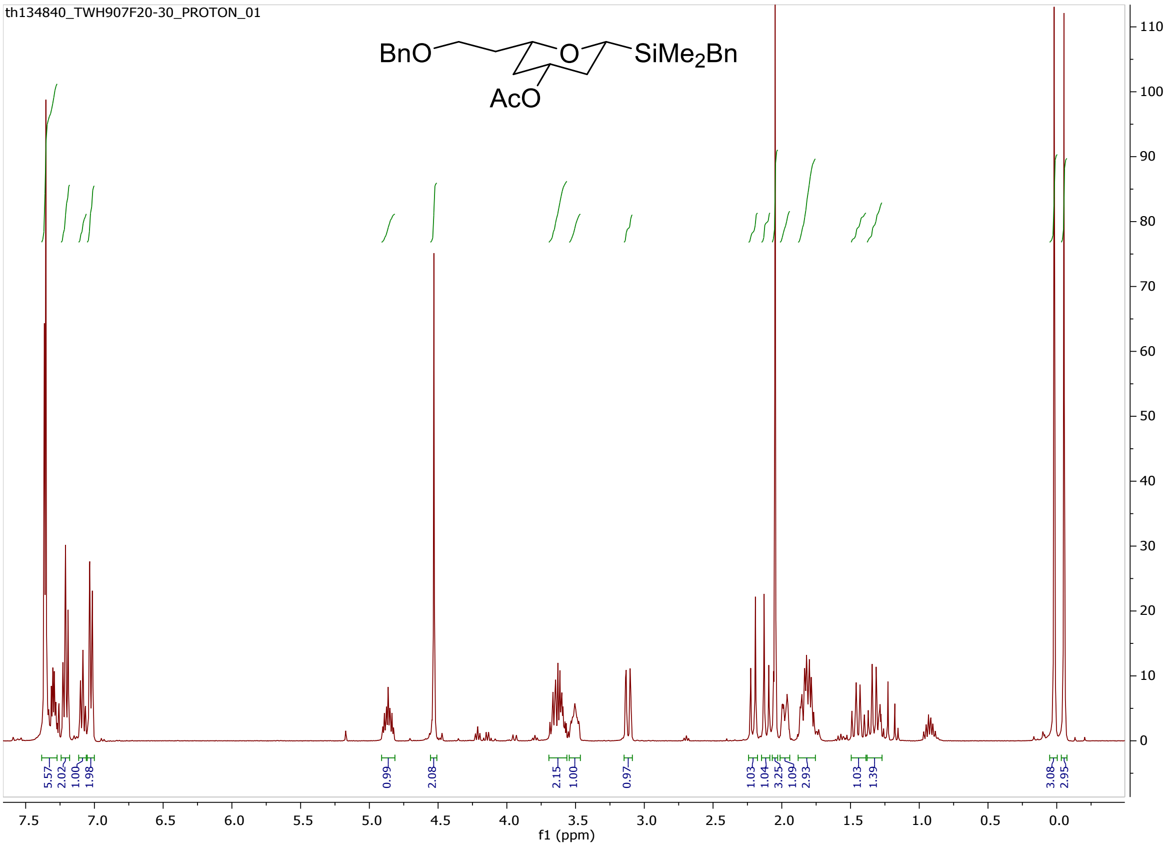

^{13}C NMR ($CDCl_3$, 100 MHz)

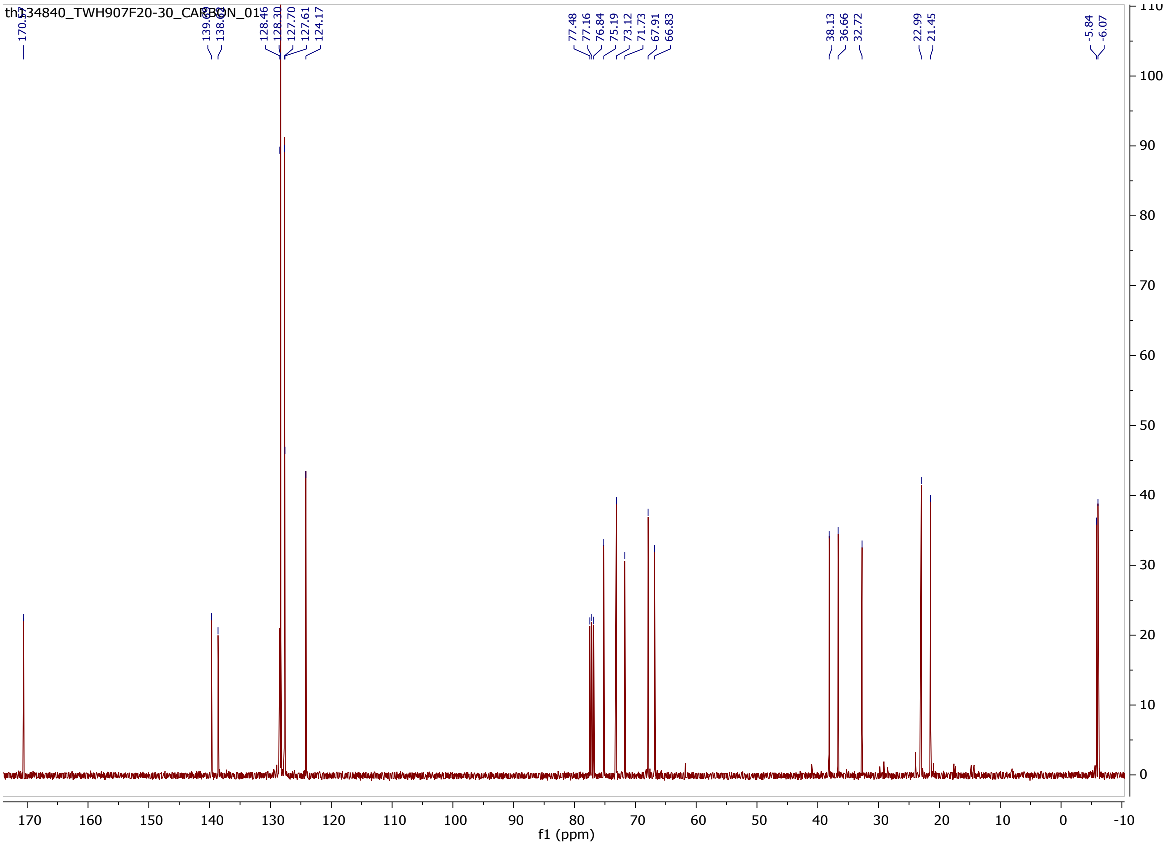

(1*S*,2*R*,3*S*,5*R*)-3-*O*-Acetyl-1-(dimethyl(benzyl)silyl)-2-methyl-5-(2’-phenylethyl)-tetrahydropyran 18

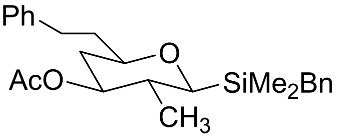

^{1}H NMR ($CDCl_3$, 400 MHz)

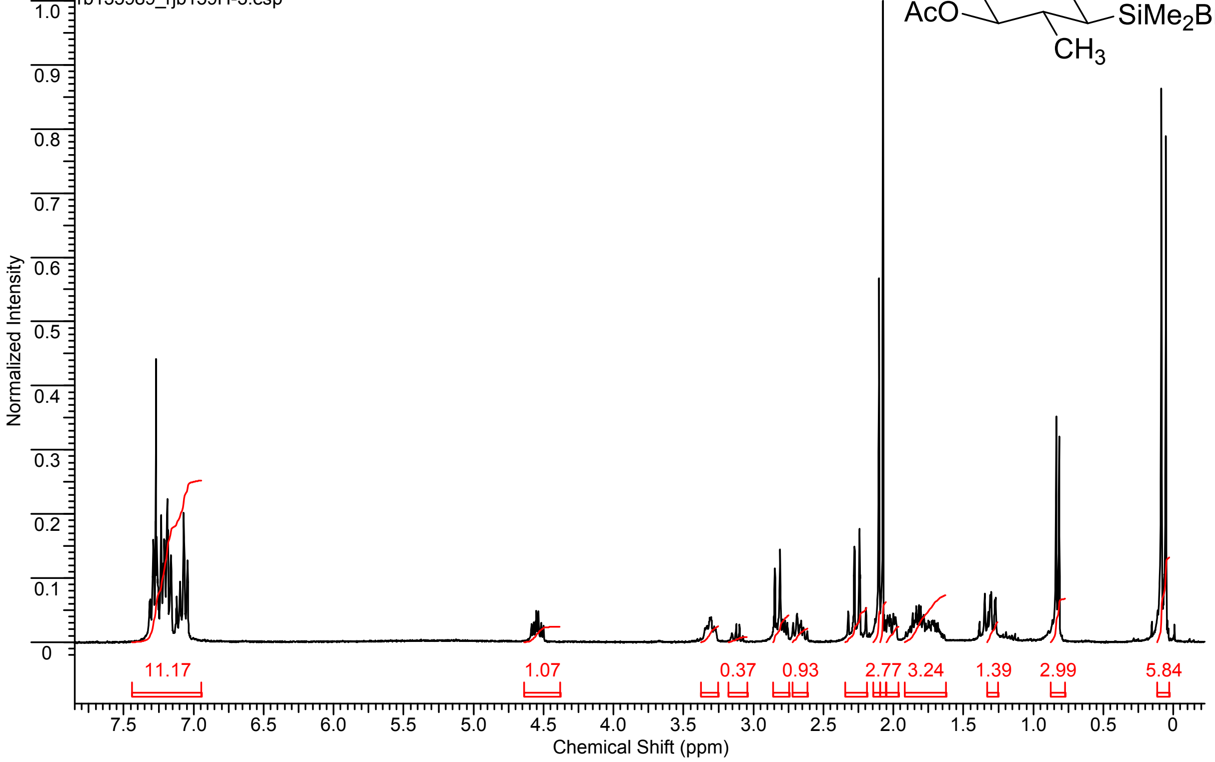

^{13}C NMR ($CDCl_3$, 100 MHz)

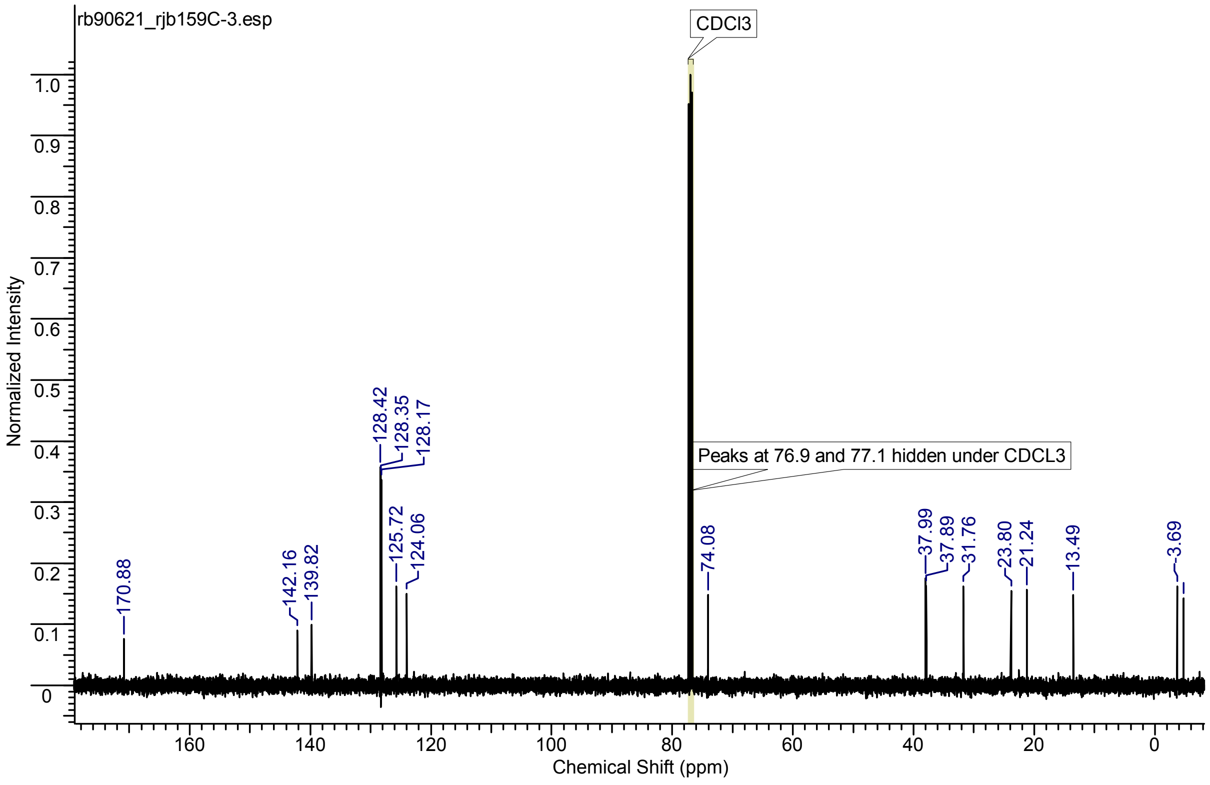

(1*S*,3*S*,5*R*)-3-*O*-Acetyl-1-(dimethyl(benzyl)silyl)-5-(2'-phenylethyl)-tetrahydropyran **19**

^{1}H NMR ($CDCl_3$, 400 MHz)

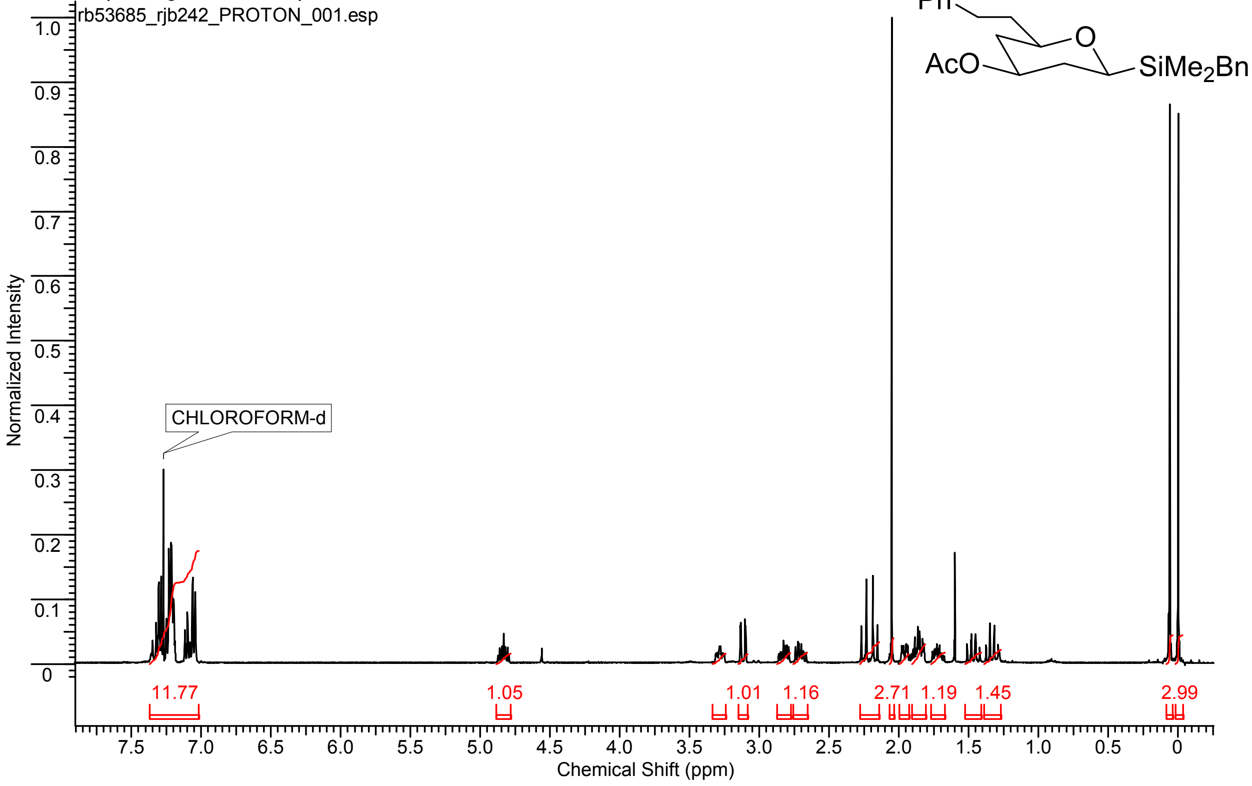

^{13}C NMR ($CDCl_3$, 100 MHz)

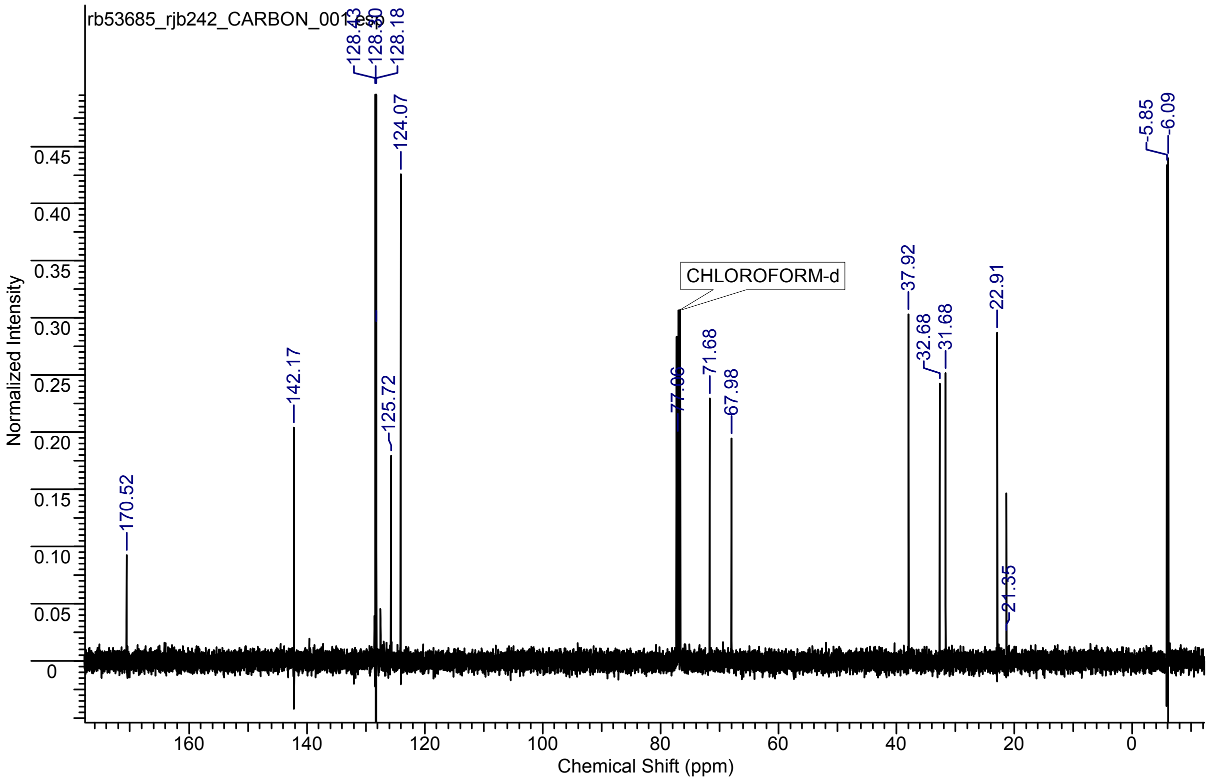

(1*S*,2*R*,3*S*,5*R*)-3-*O*-Benzyl-1-(dimethyl(benzyl)silyl)-2-methyl-5-(2'-phenylethyl)-tetrahydropyran 20

^{1}H NMR ($CDCl_3$, 400 MHz)

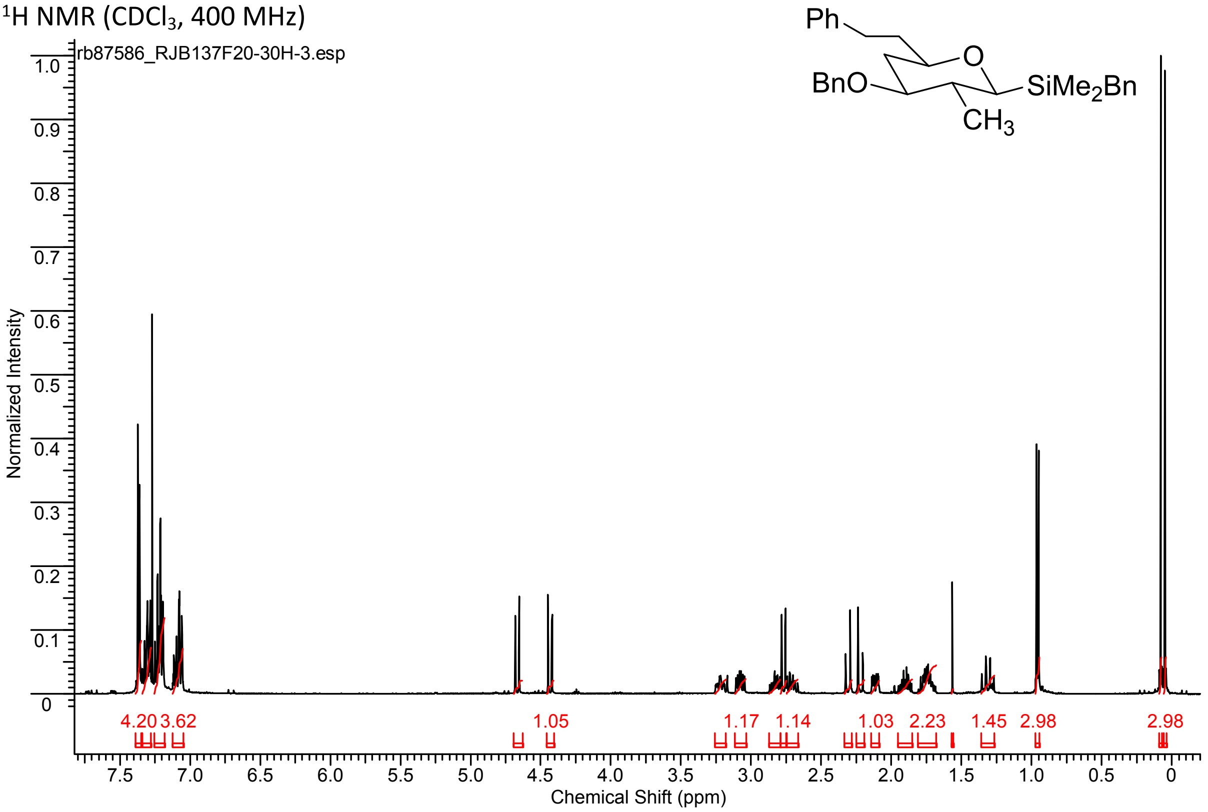

^{13}C NMR ($CDCl_3$, 100 MHz)

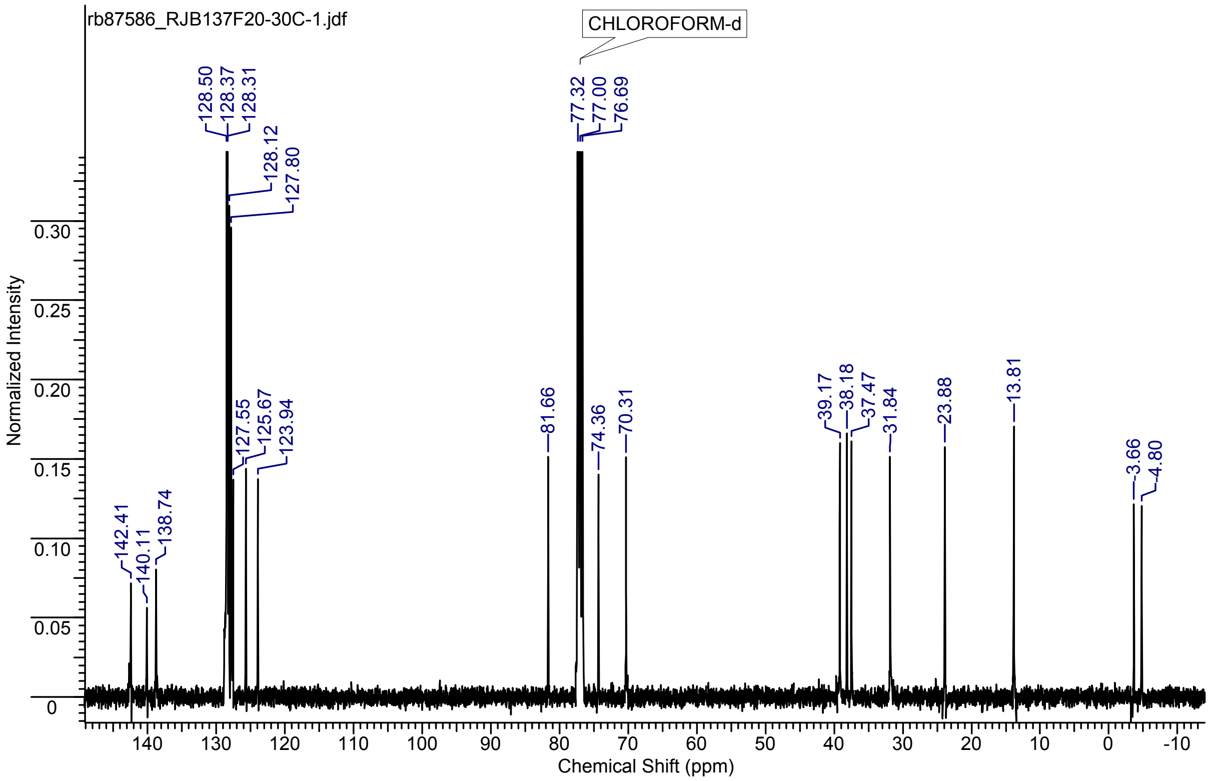

(1*S*,3*S*,5*R*)-3-*O*-Benzyl-1-(dimethyl(benzyl)silyl)-5-ethyl-(2'-phenylethyl)-tetrahydropyran 21

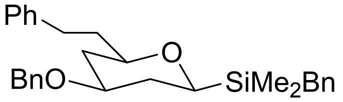

^{1}H NMR ($CDCl_3$, 400 MHz)

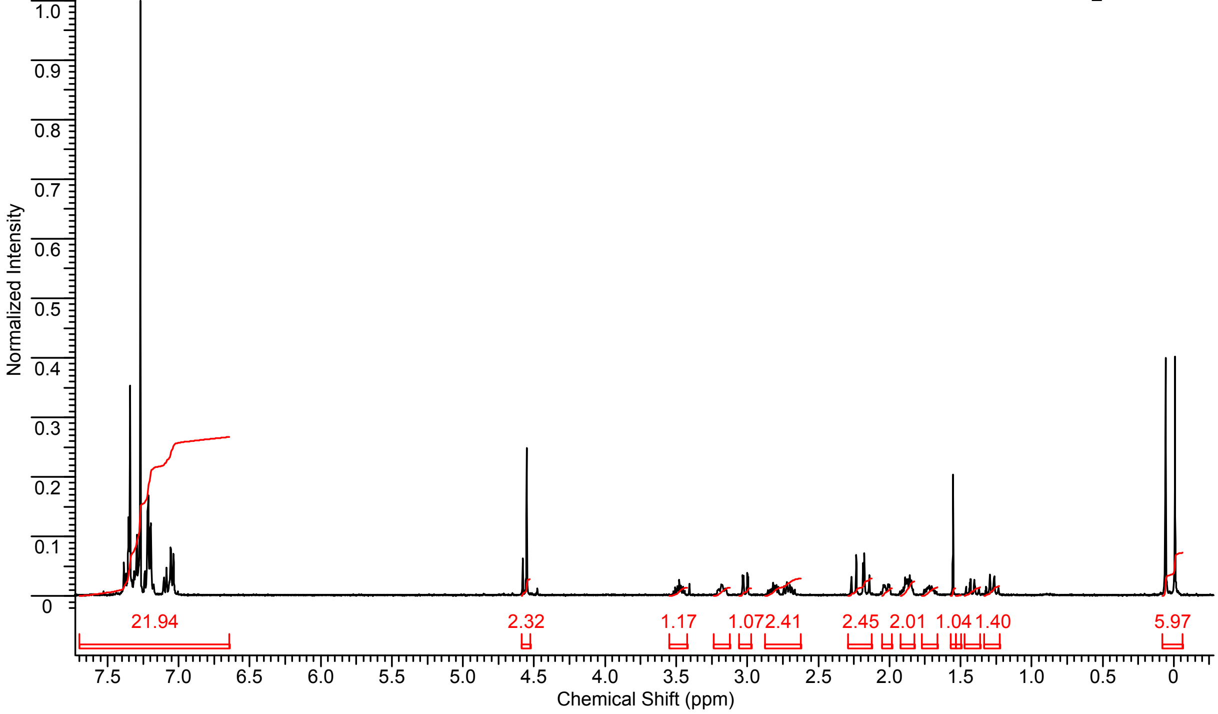

^{13}C NMR ($CDCl_3$, 100 MHz)

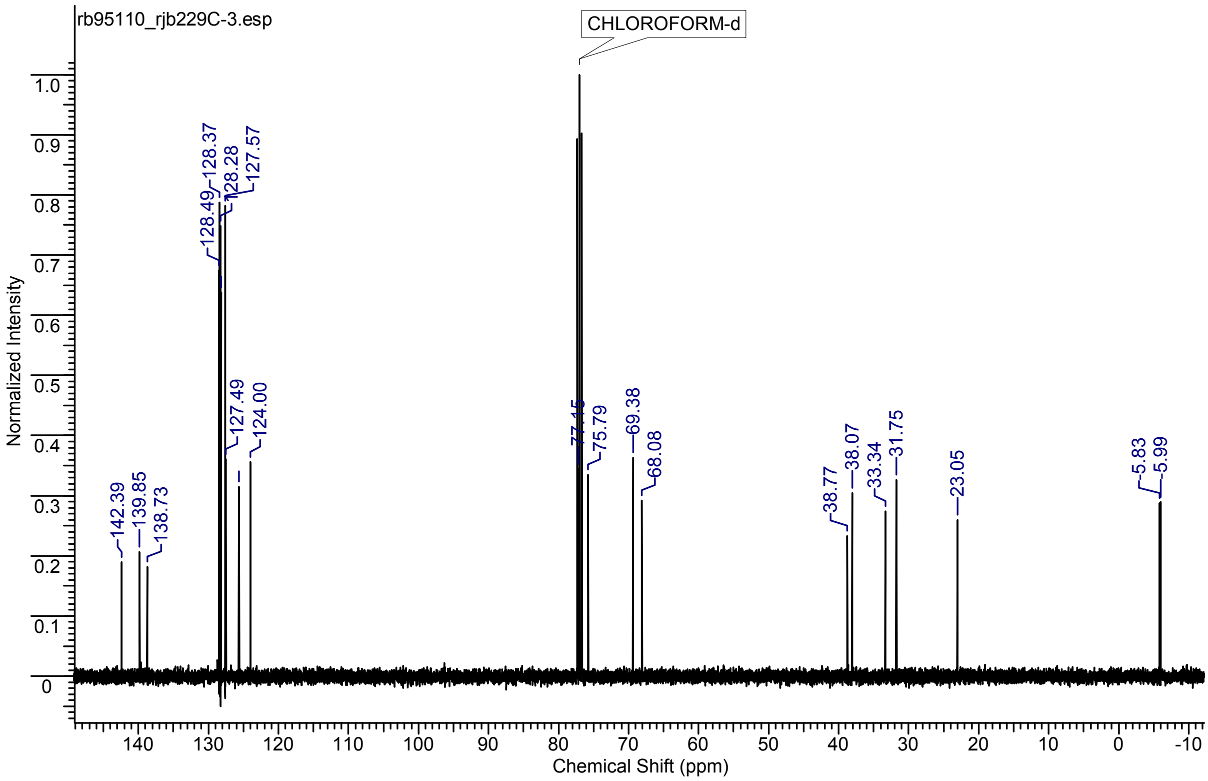

(3*S*,5*R*)-1,3-*O*-Acetyl-2-methyl-5-(2'-phenylethyl)-tetrahydropyran 17

^{1}H NMR ($CDCl_3$, 400 MHz)

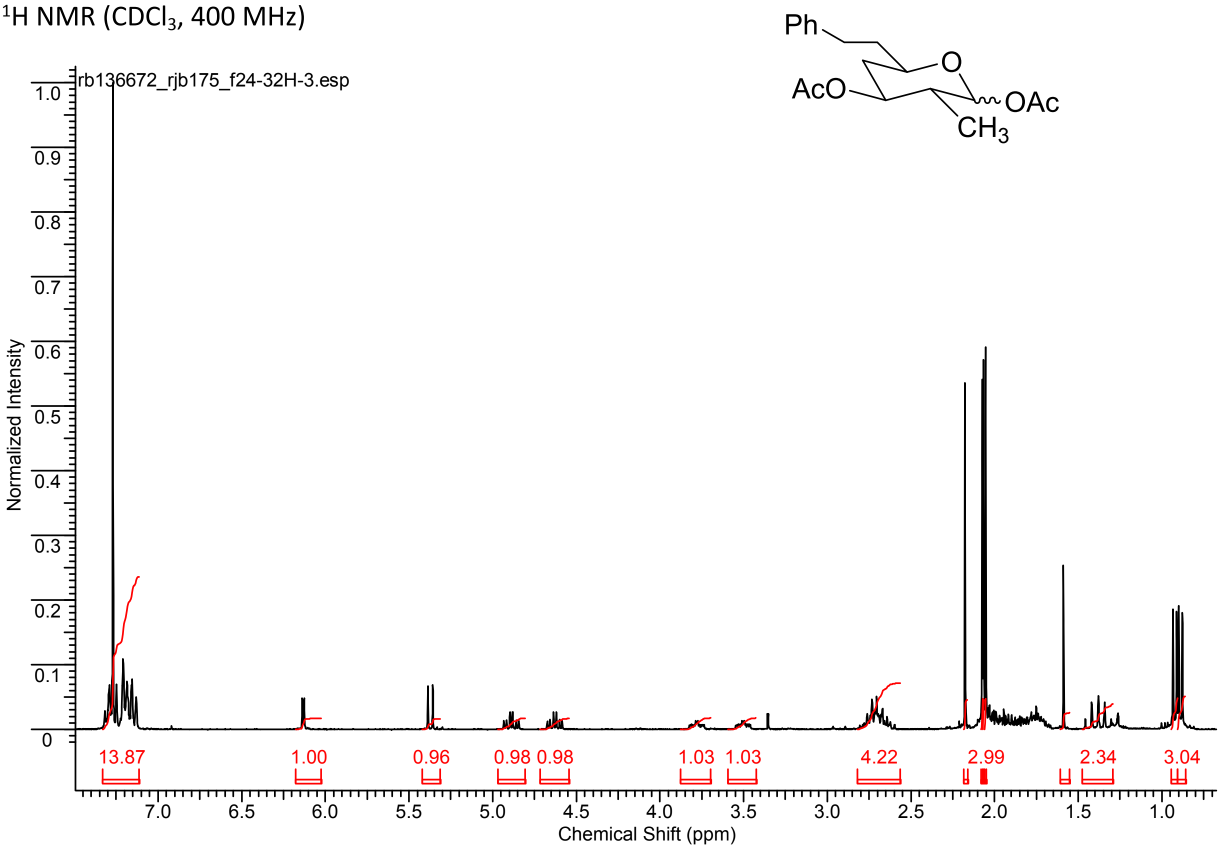

^{13}C NMR ($CDCl_3$, 100 MHz)

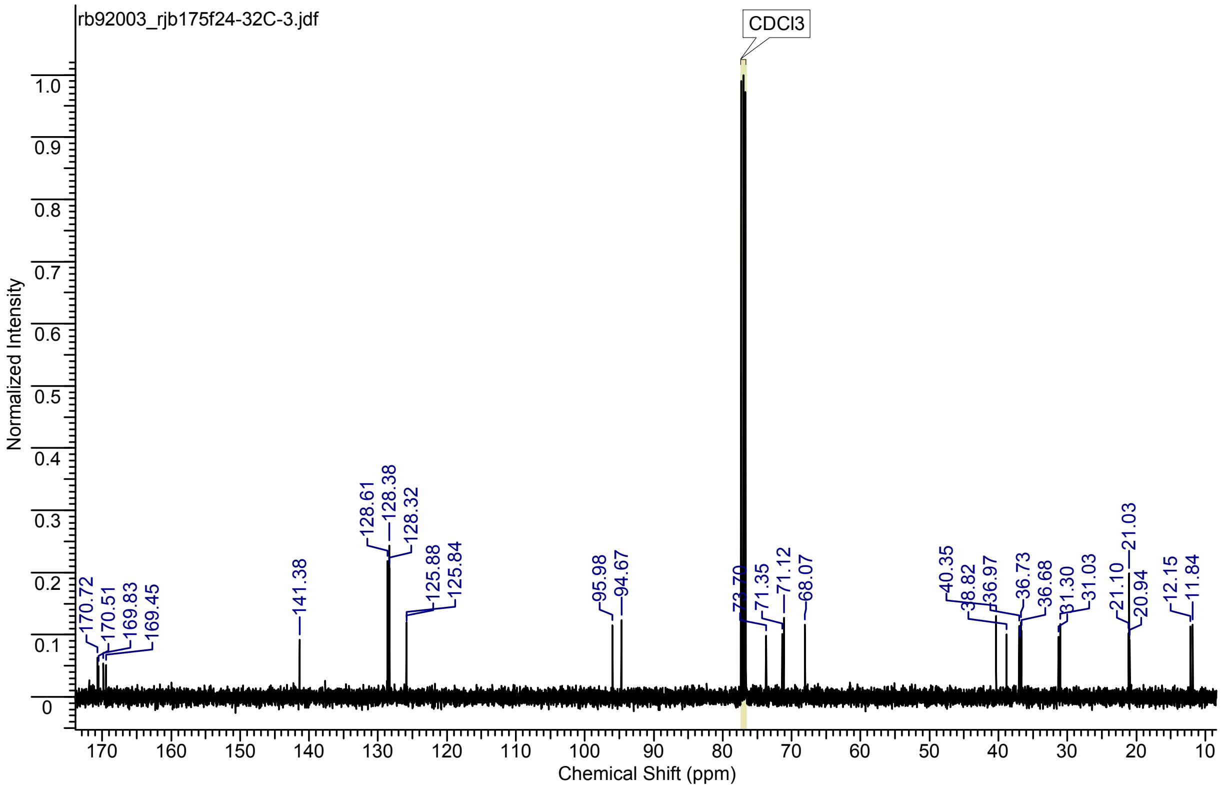

(3*S*,5*R*)-1,3-*O*-Acetyl-5-(2'-phenylethyl)-tetrahydropyran 22

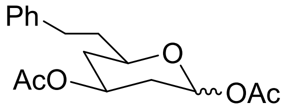

^{1}H NMR ($CDCl_3$, 400 MHz)

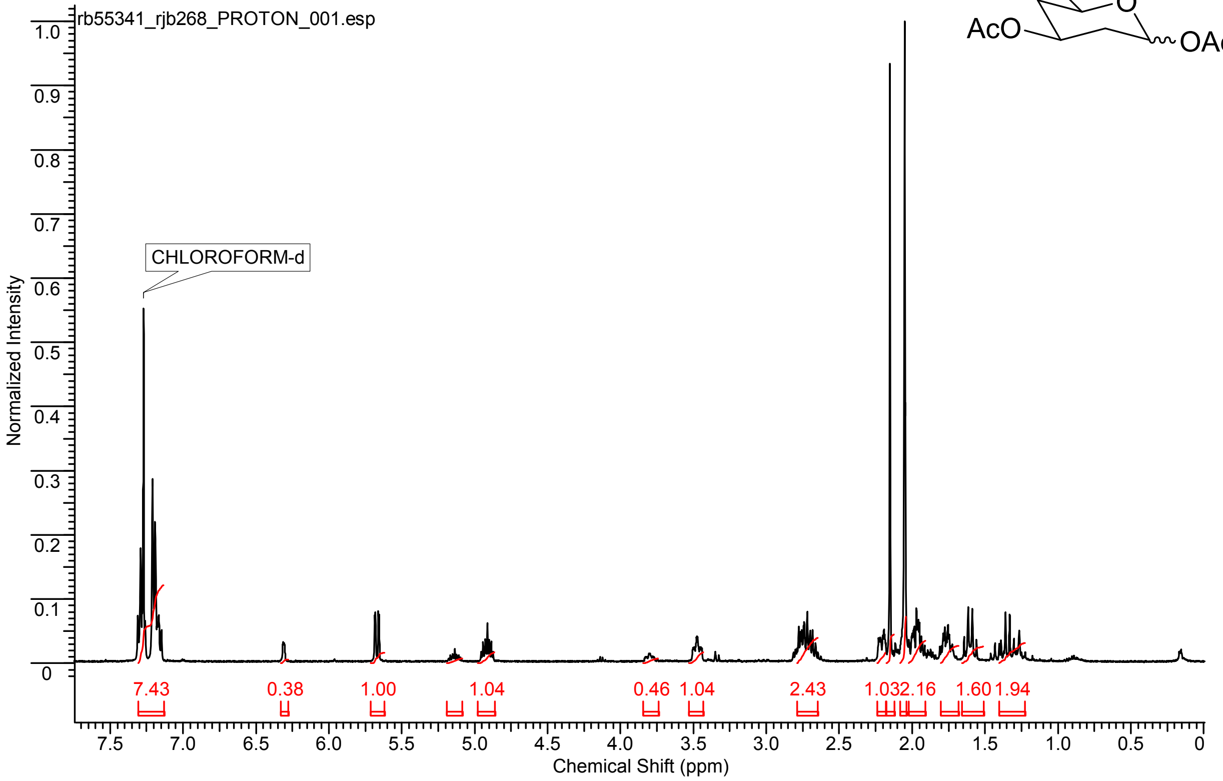

^{13}C NMR ($CDCl_3$, 100 MHz)

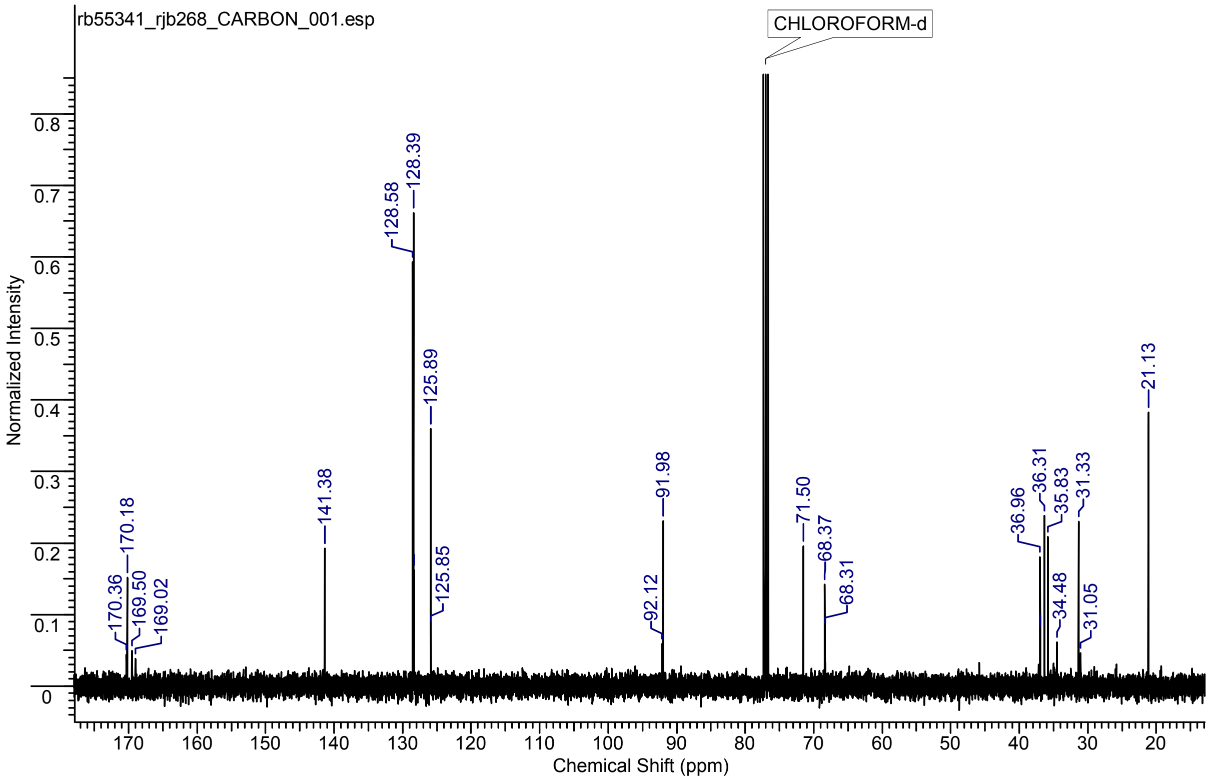

(2*R*,3*S*,5*R*)-1-*O*-Acetyl-3-*O*-Benzyl-2-methyl-5-(2'-phenylethyl)-tetrahydropyran 23

^{1}H NMR ($CDCl_3$, 400 MHz)

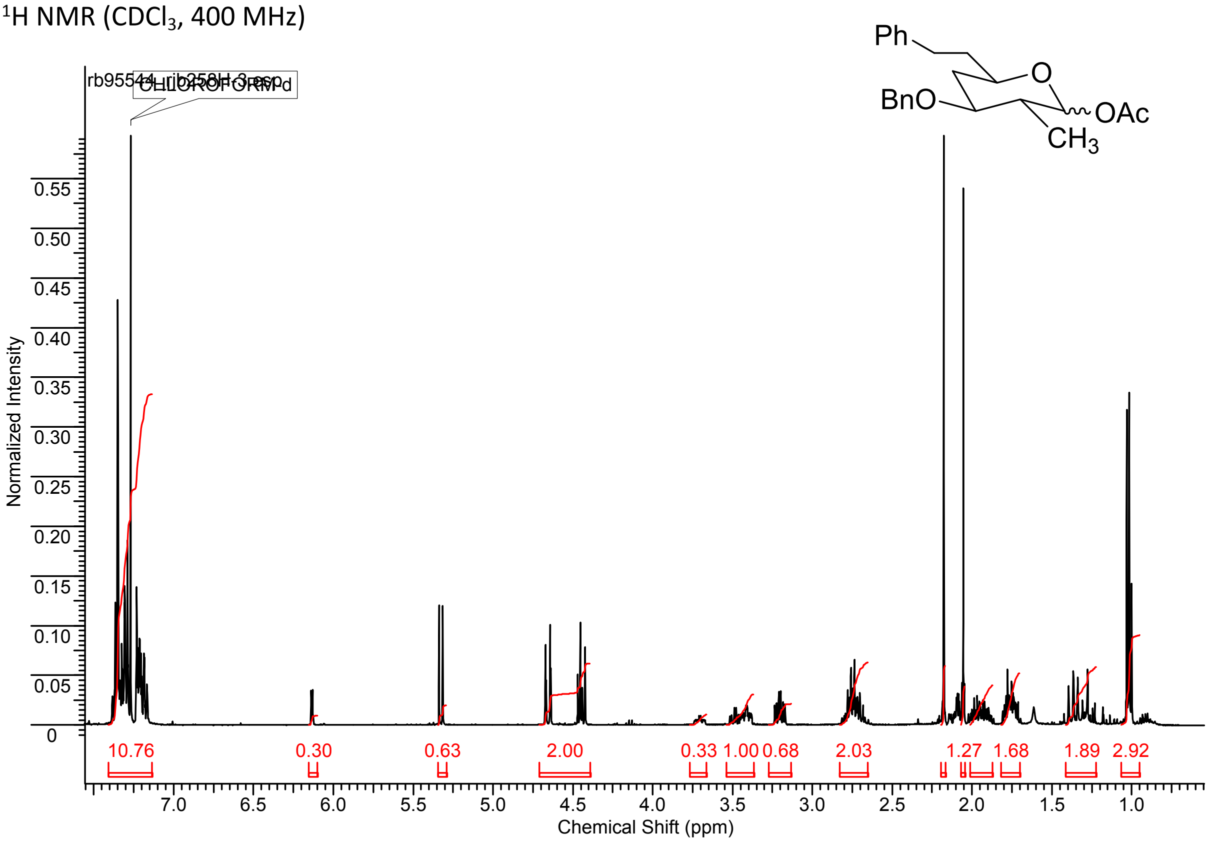

^{13}C NMR ($CDCl_3$, 100 MHz)

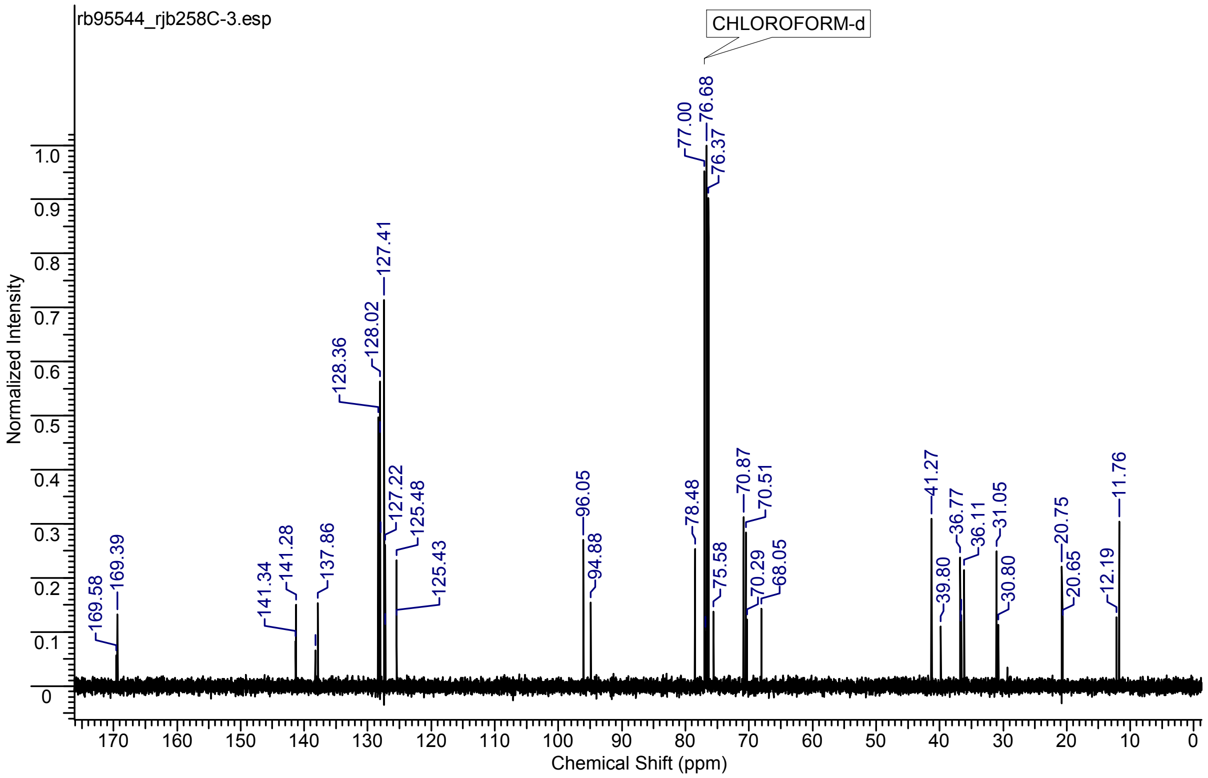

((3*S*,5*R*)-1-*O*-Acetyl-3-*O*-Benzyl-5-(2'-phenylethyl)-tetrahydropyran 24

^{1}H NMR ($CDCl_3$, 400 MHz)

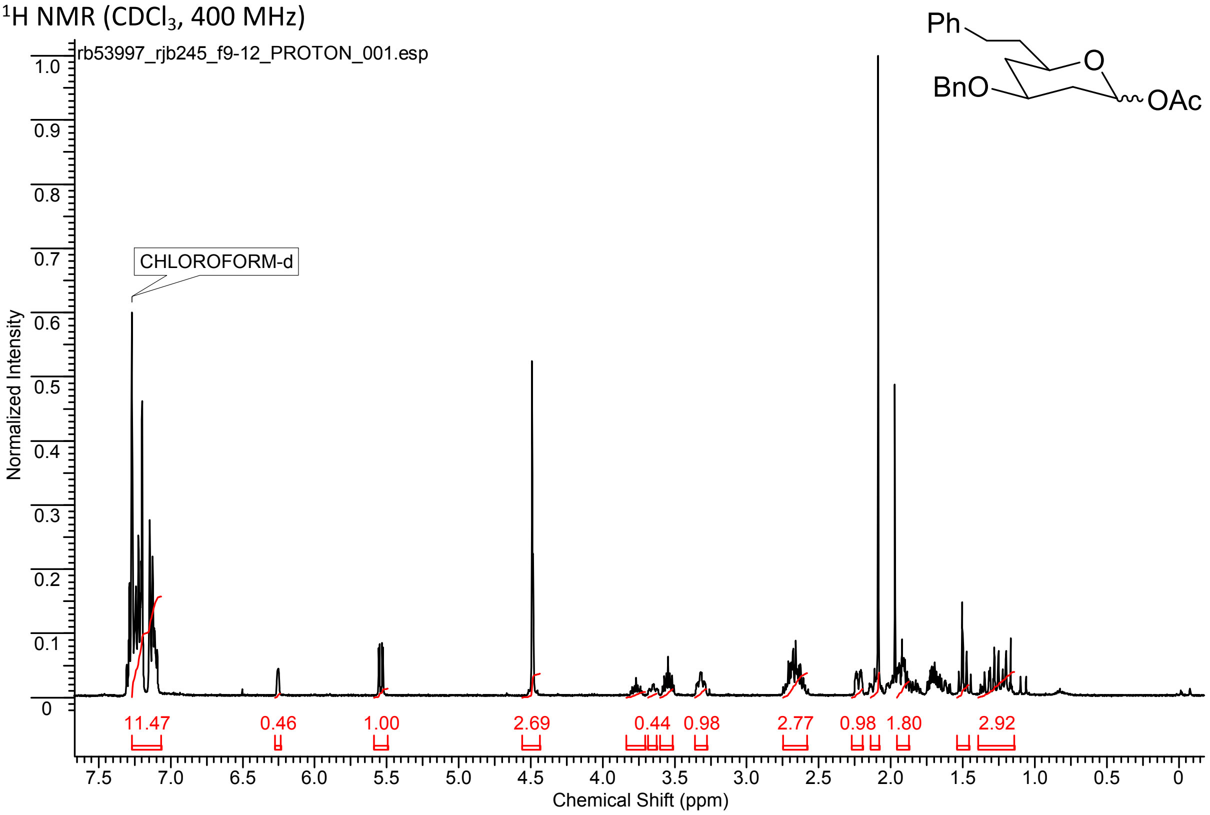

^{13}C NMR ($CDCl_3$, 100 MHz)

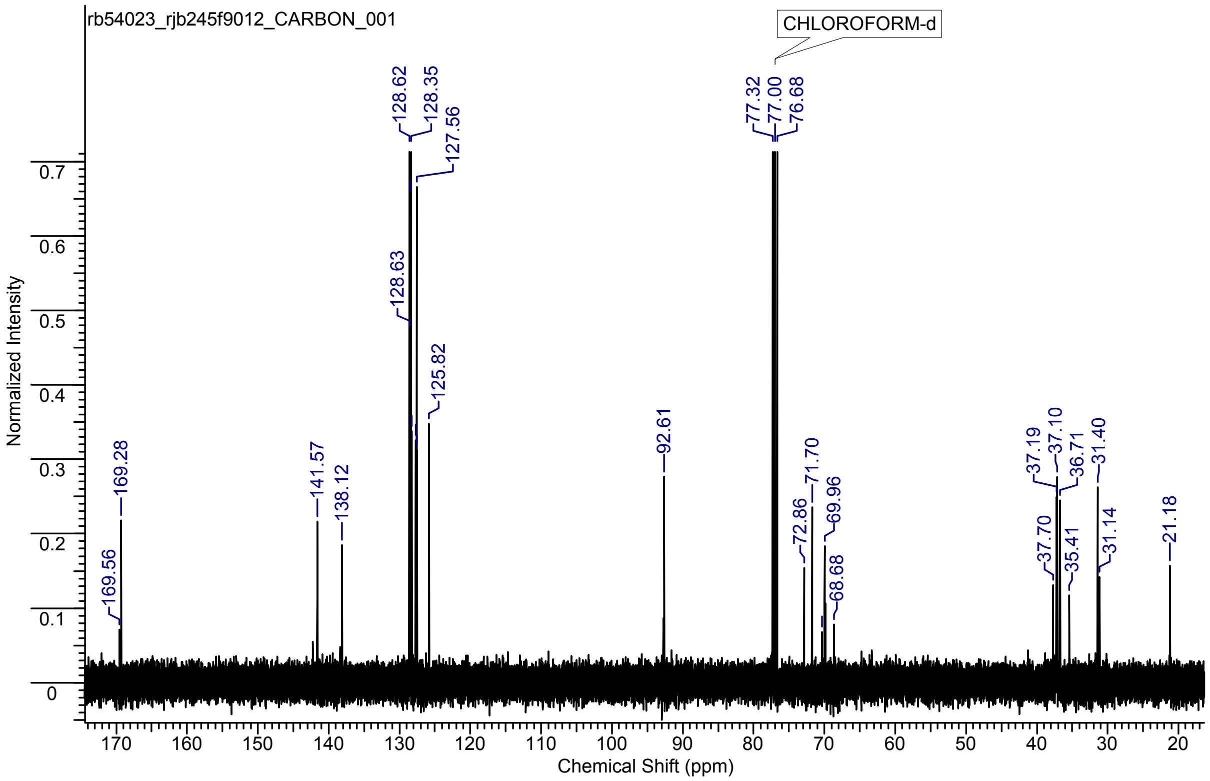

(1*S*, 3*S*, 5*S*)-3-*O*-Acetyl-5-acetoxymethyl-1-(benzyldimethylsilyl)-tetrahydropyran 27

^{1}H NMR ($CDCl_3$, 400 MHz)

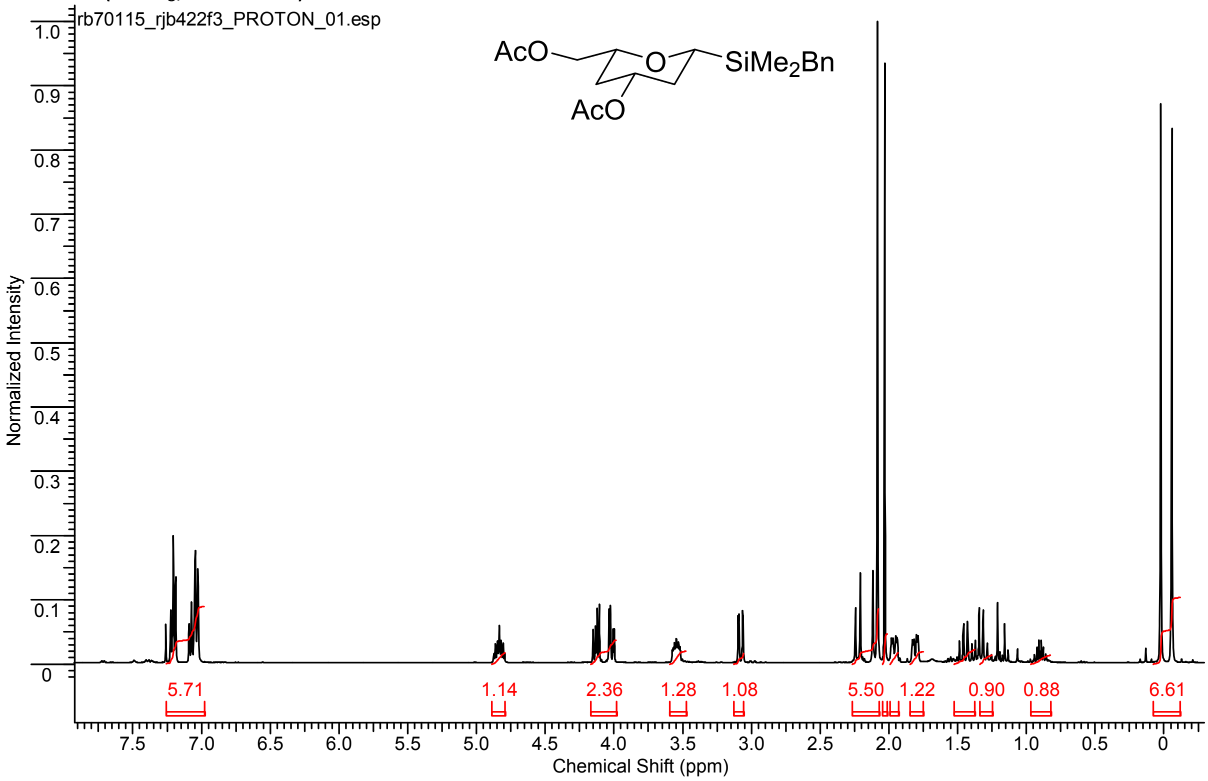

^{13}C NMR ($CDCl_3$, 100 MHz)

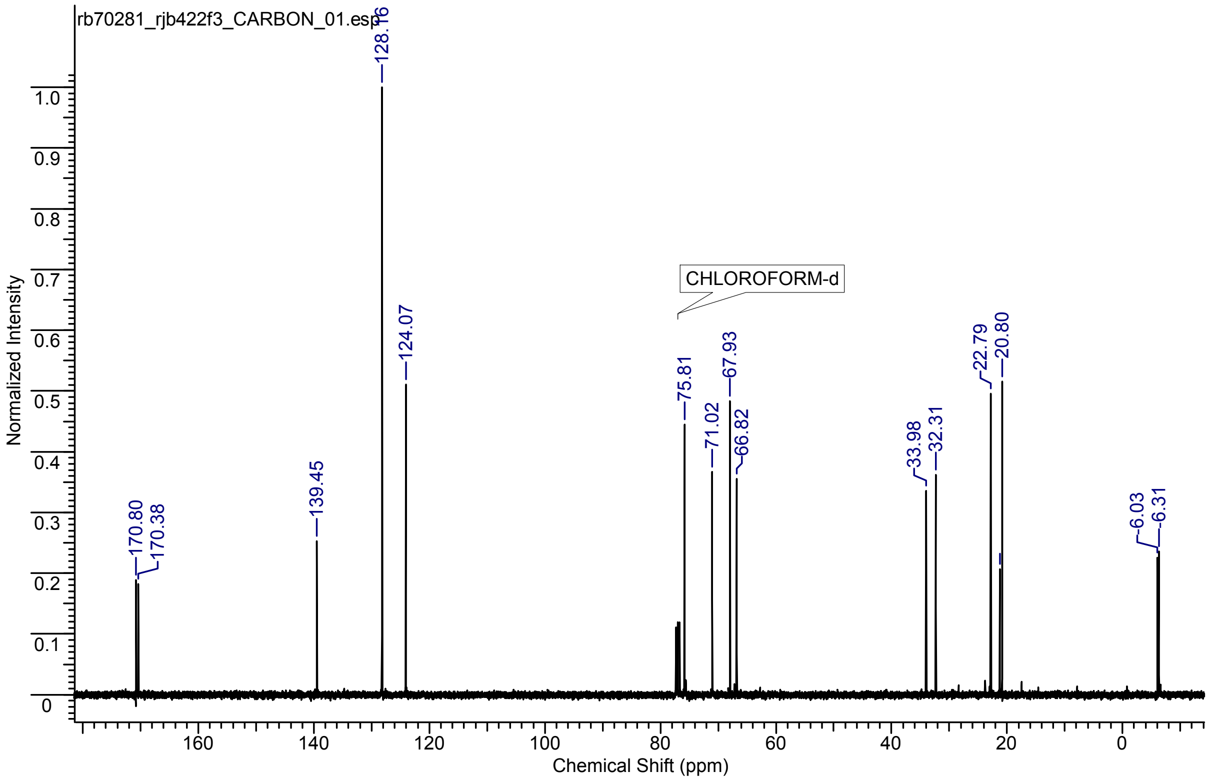

2,4-Dideoxy-gluc-3,5-diacetate hexopyranose 28

^{1}H NMR ($CDCl_3$, 400 MHz)

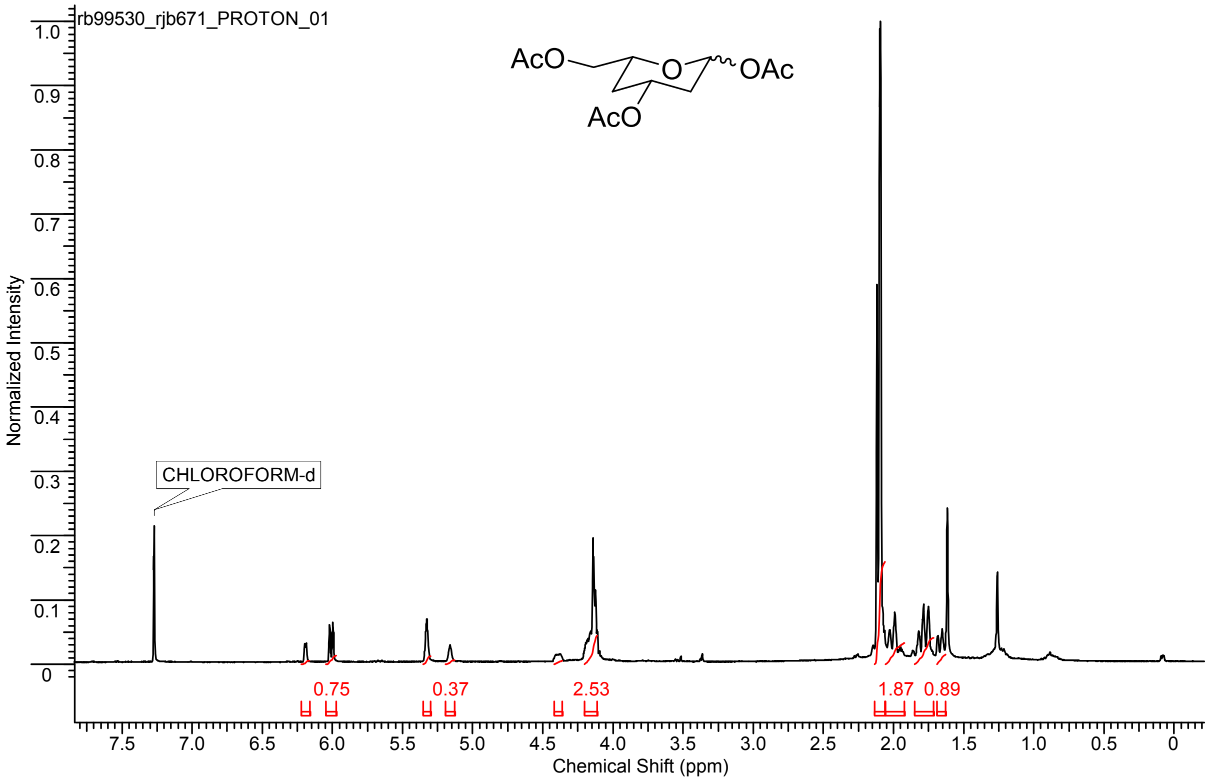

^{13}C NMR (100MHz, $CDCl_3$)

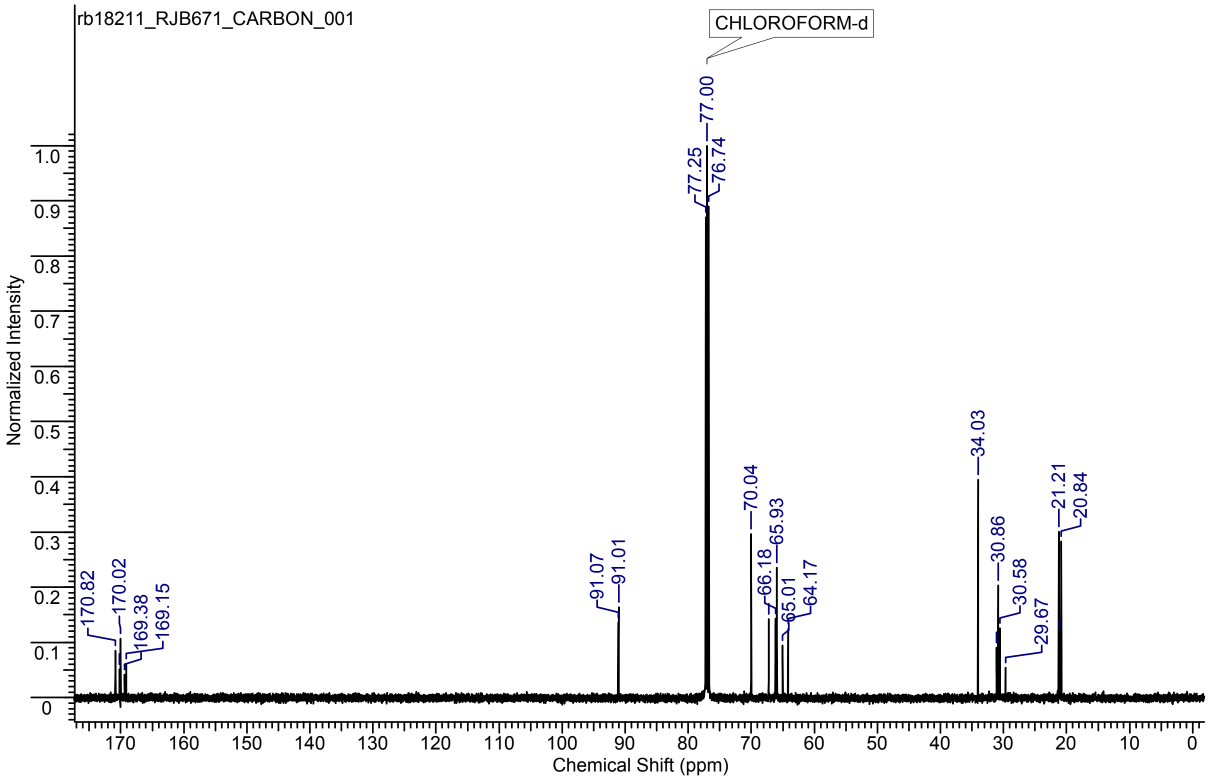

(1*S*, 3*S*, 5*S*)-1-(Benzyldimethylsilyl)-3-ethoxy-5-((*tert*-butyldiphenylsilyloxy)methyl) – tetrahydropyran 29

^{1}H NMR ($CDCl_3$, 400 MHz)

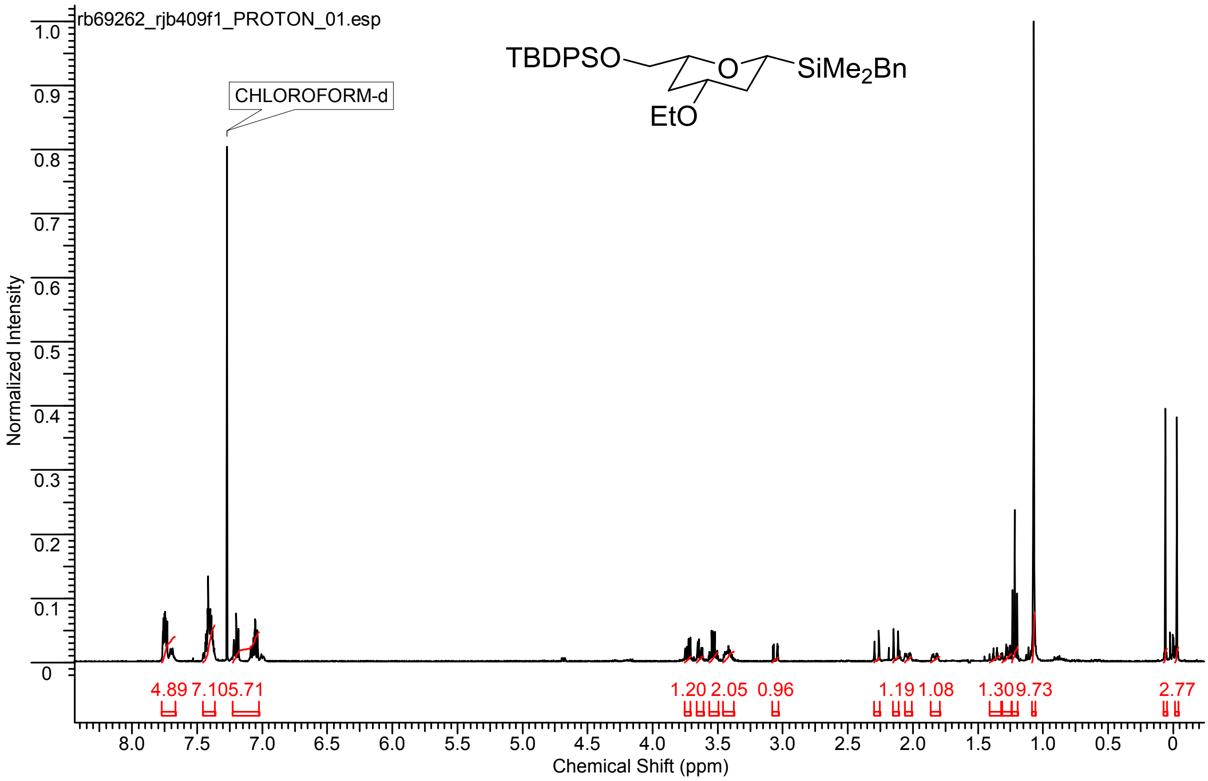

^{13}C NMR ($CDCl_3$, 100 MHz)

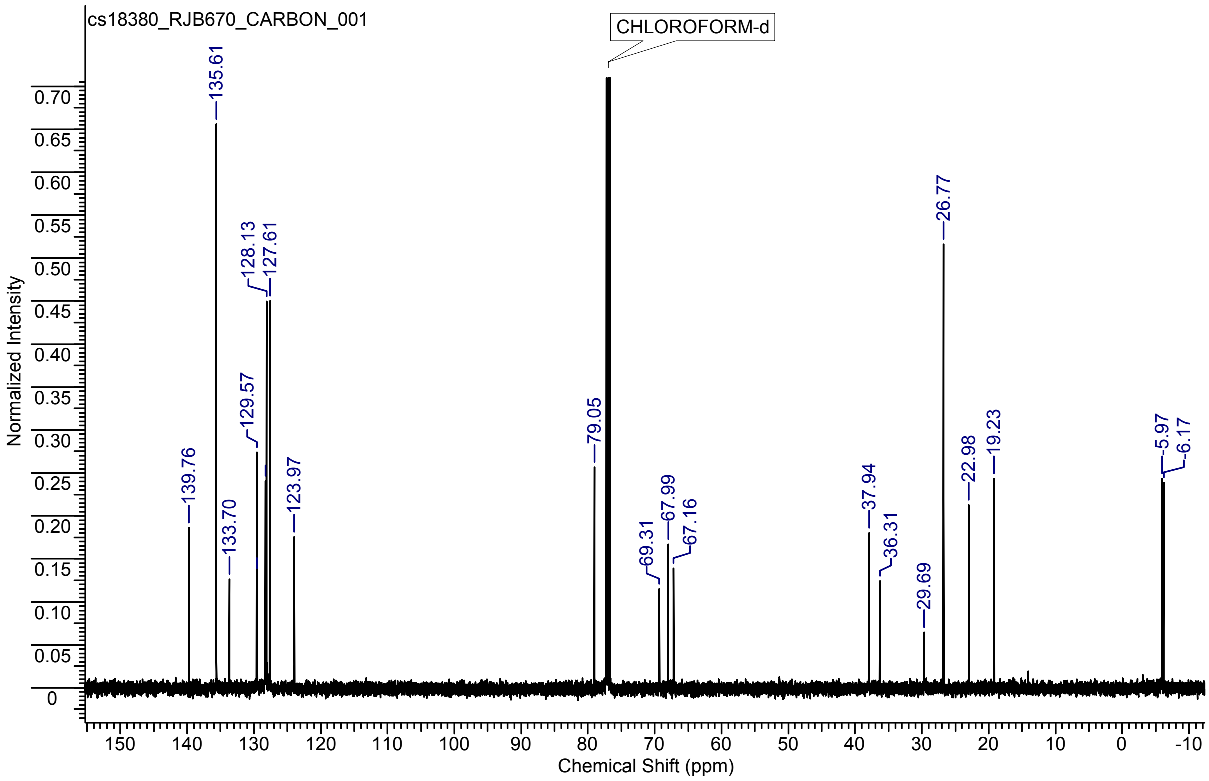

(1*S*,3*S*,5*S*)-3-*O*-Acetyl-1-(benzyldimethylsilyl)-5-(1'-(benzyloxy)methyl)-tetrahydropyran 30

^{1}H NMR ($CDCl_3$, 400 MHz)

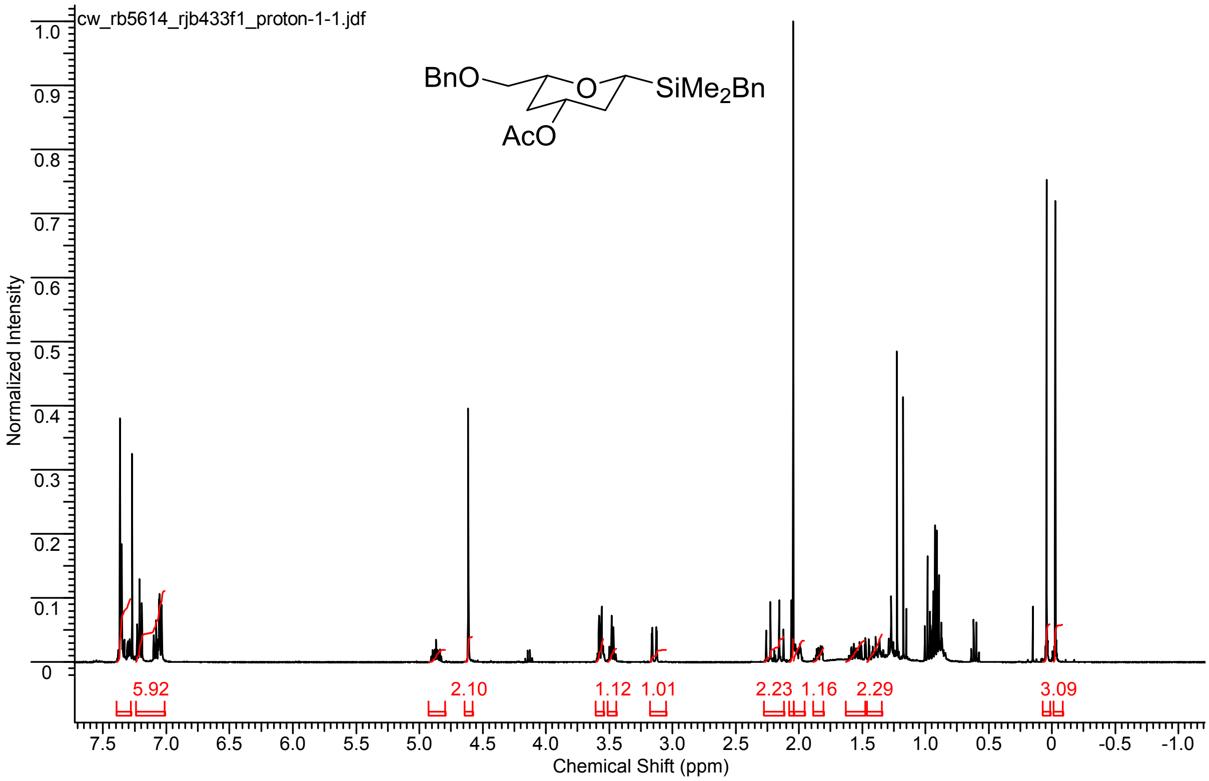

^{13}C NMR ($CDCl_3$, 100 MHz)

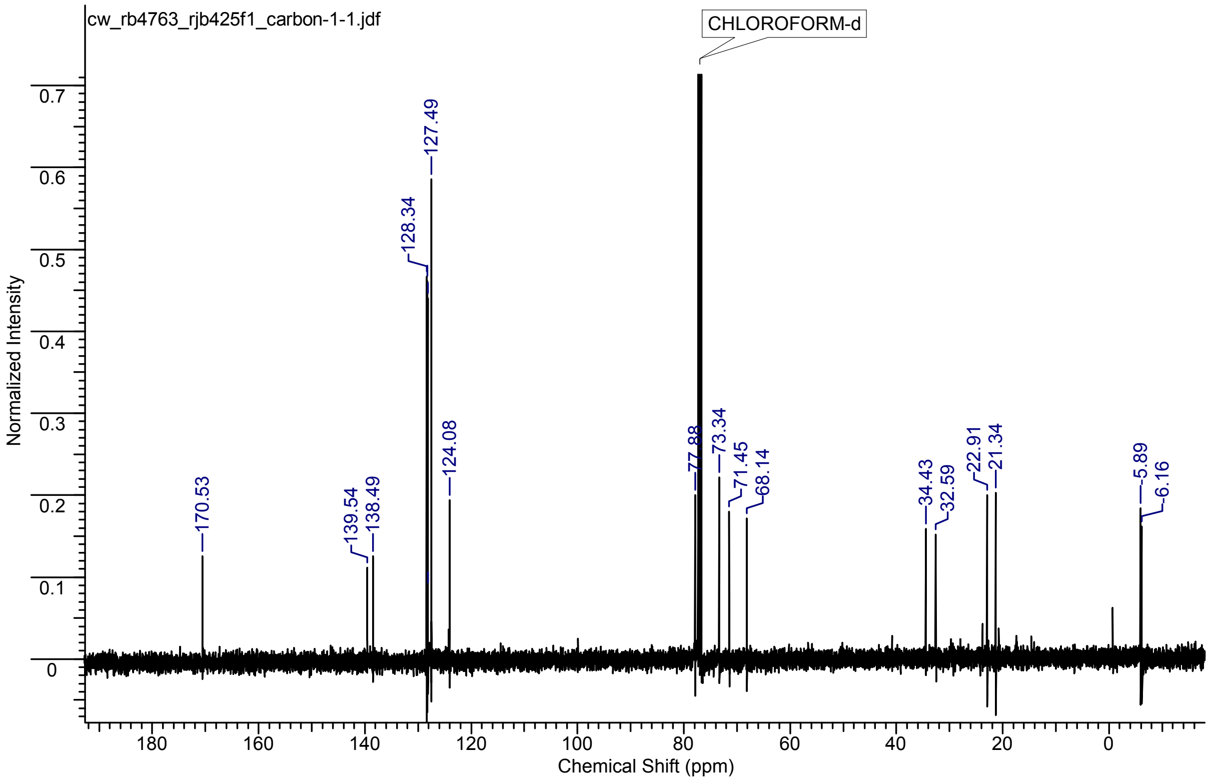

(3*R*, 5*R*)-1,3-*O*-Acteyl-5-(1'-benzyloxymethyl)-tetrahydropyran 31

^{1}H NMR ($CDCl_3$, 400 MHz)

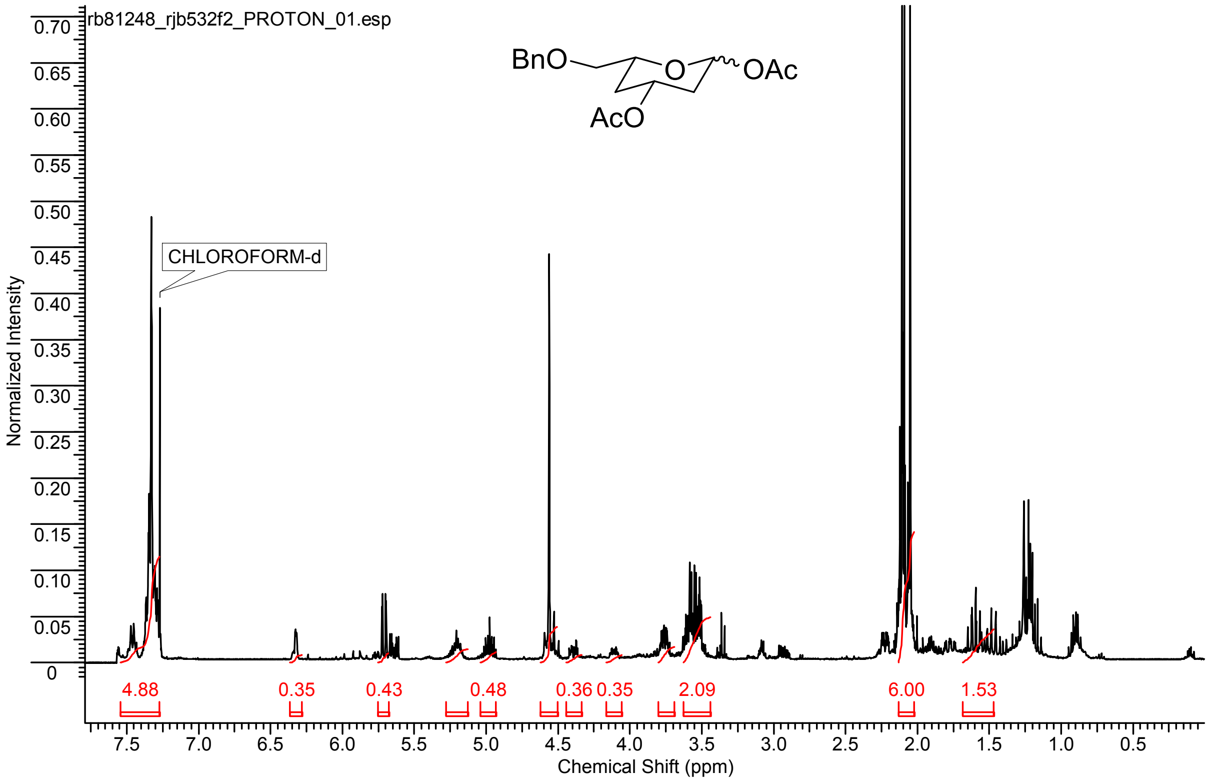

^{13}C NMR ($CDCl_3$, 100 MHz)

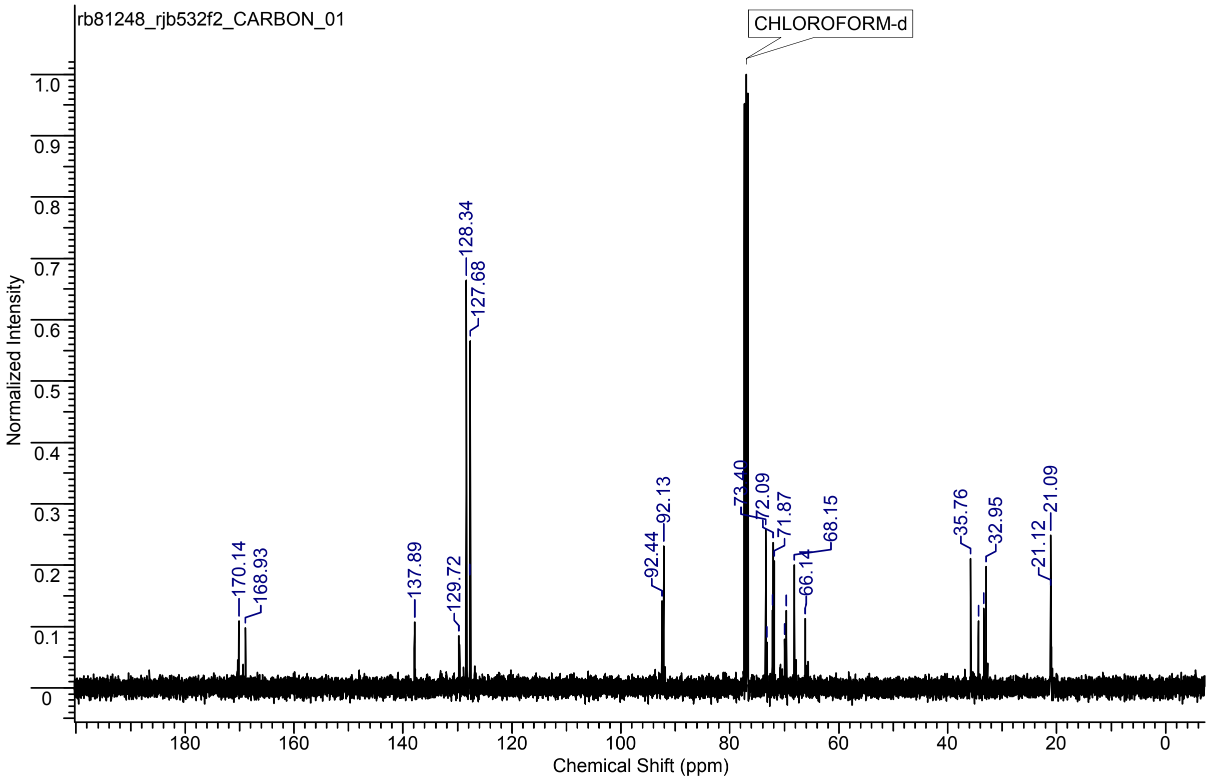

(1R,3*S*,5*S*)-3-*O*-Acetyl-1-O-cyclohexyl-5-(1’-(benzyloxy)methyl)-tetrahydropyran 32

^{1}H NMR ($CDCl_3$, 400 MHz)

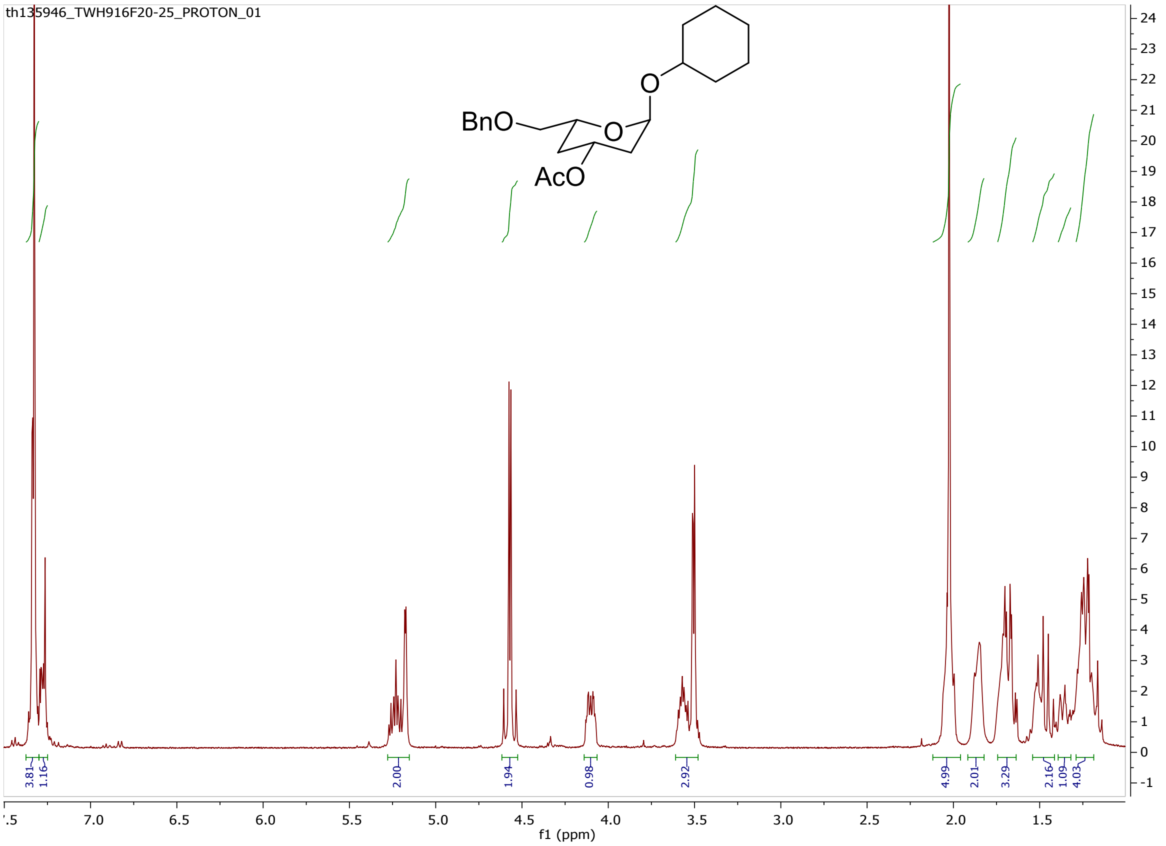

^{13}C NMR ($CDCl_3$, 100 MHz)

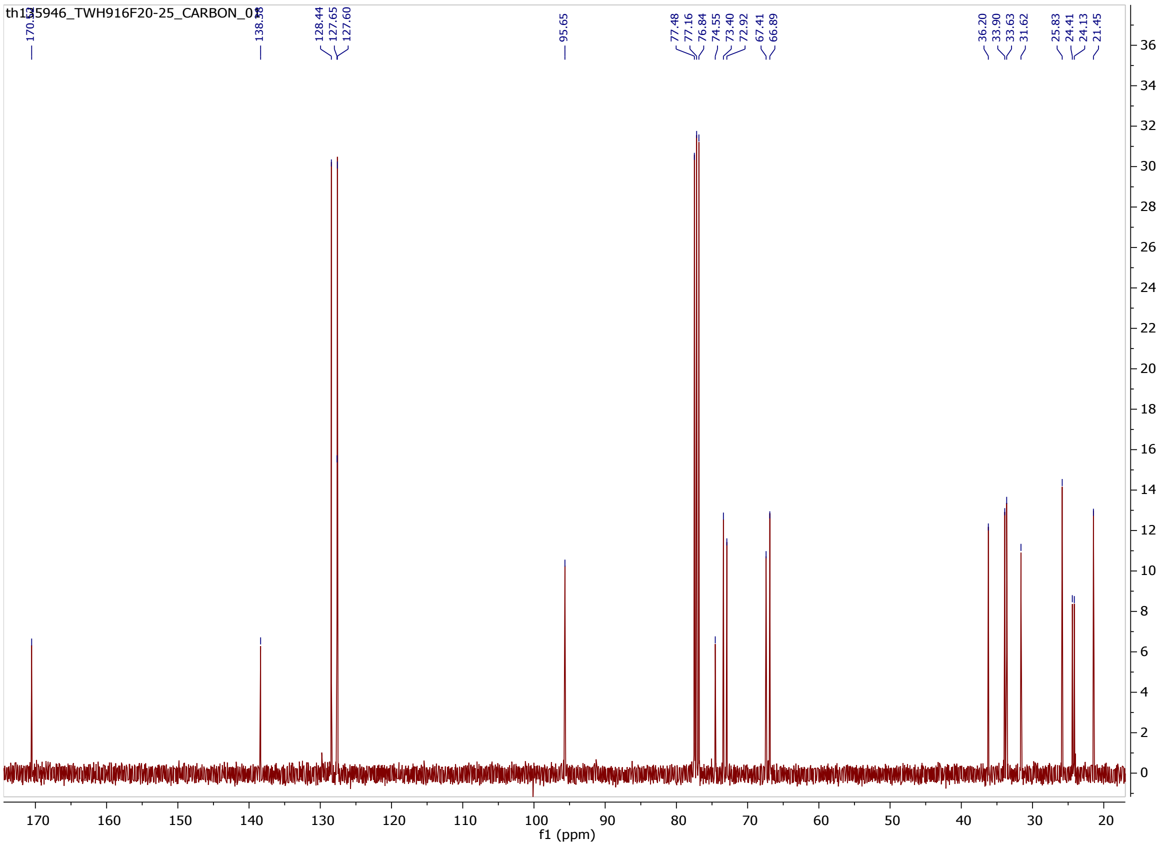

(1*R*, 3*R*, 5*R*)-1-(Benzyldimethylsilyl)-5-(1'-benzyloxymethyl)-3-hydroxy-tetrahydropyran SI-4

^{1}H NMR ($CDCl_3$, 400 MHz)

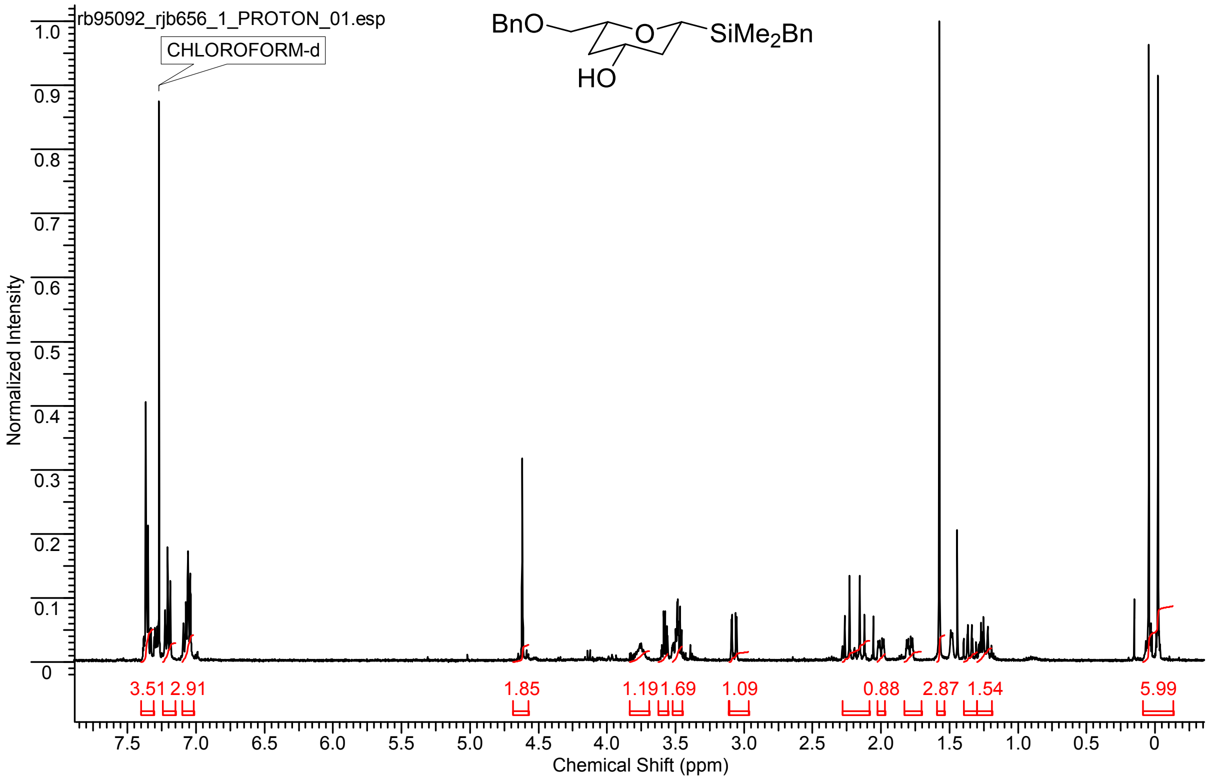

^{13}C NMR ($CDCl_3$, 100 MHz)

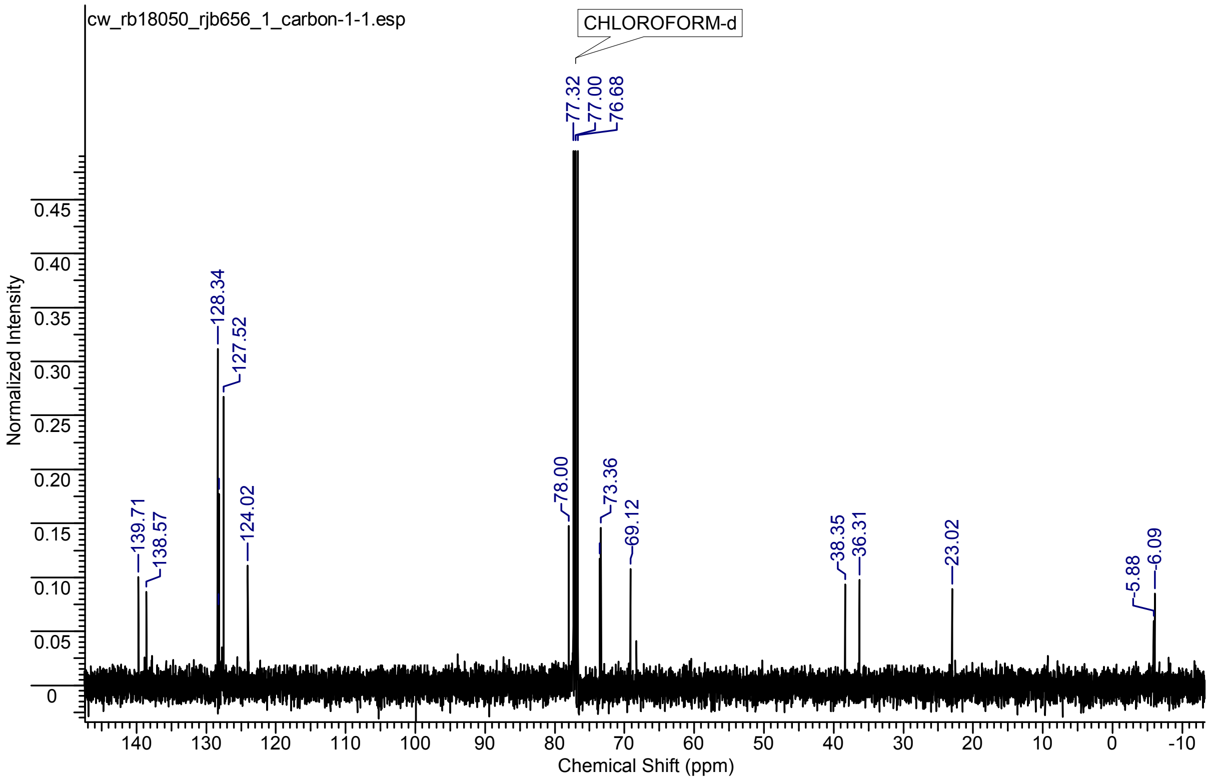

(1*R*, 3*S*, 5*R*)-1-(Benzyldimethylsilyl)-5-(1'-benzyloxymethyl)-3-hydroxy-tetrahydropyran 33

^{1}H NMR ($CDCl_3$, 400 MHz)

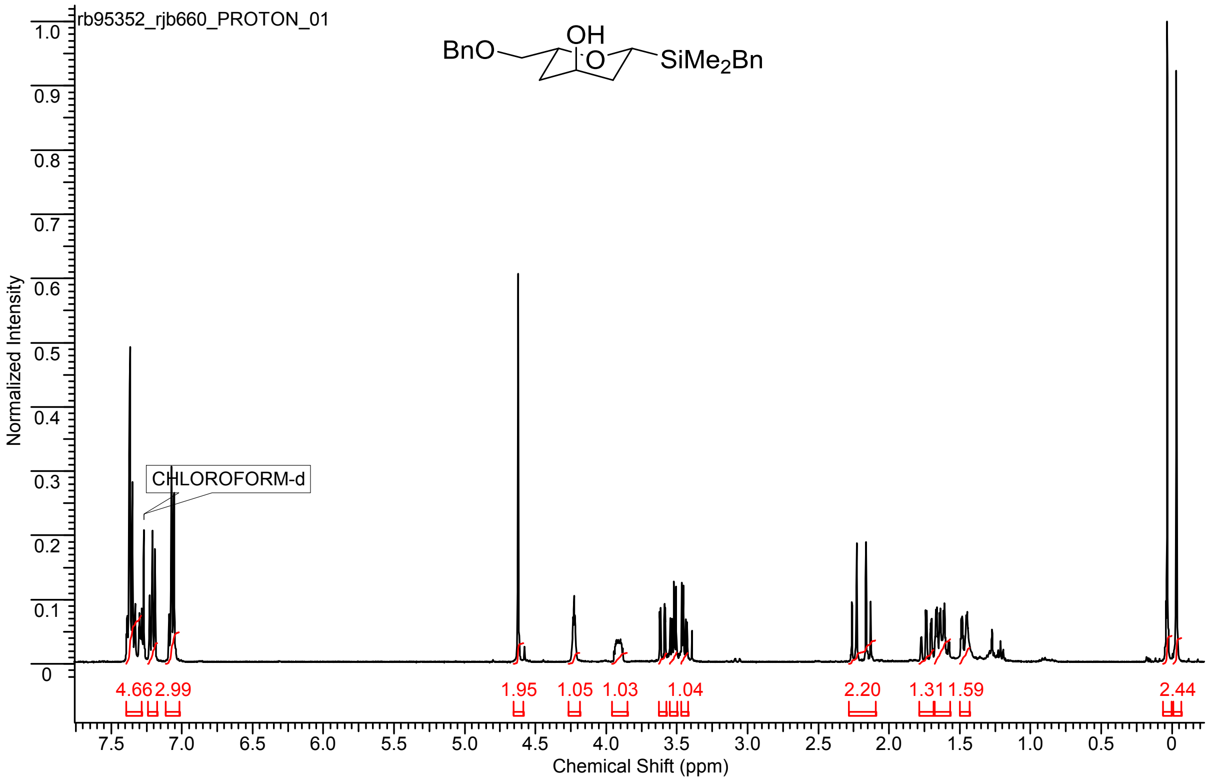

^{13}C NMR ($CDCl_3$, 100 MHz)

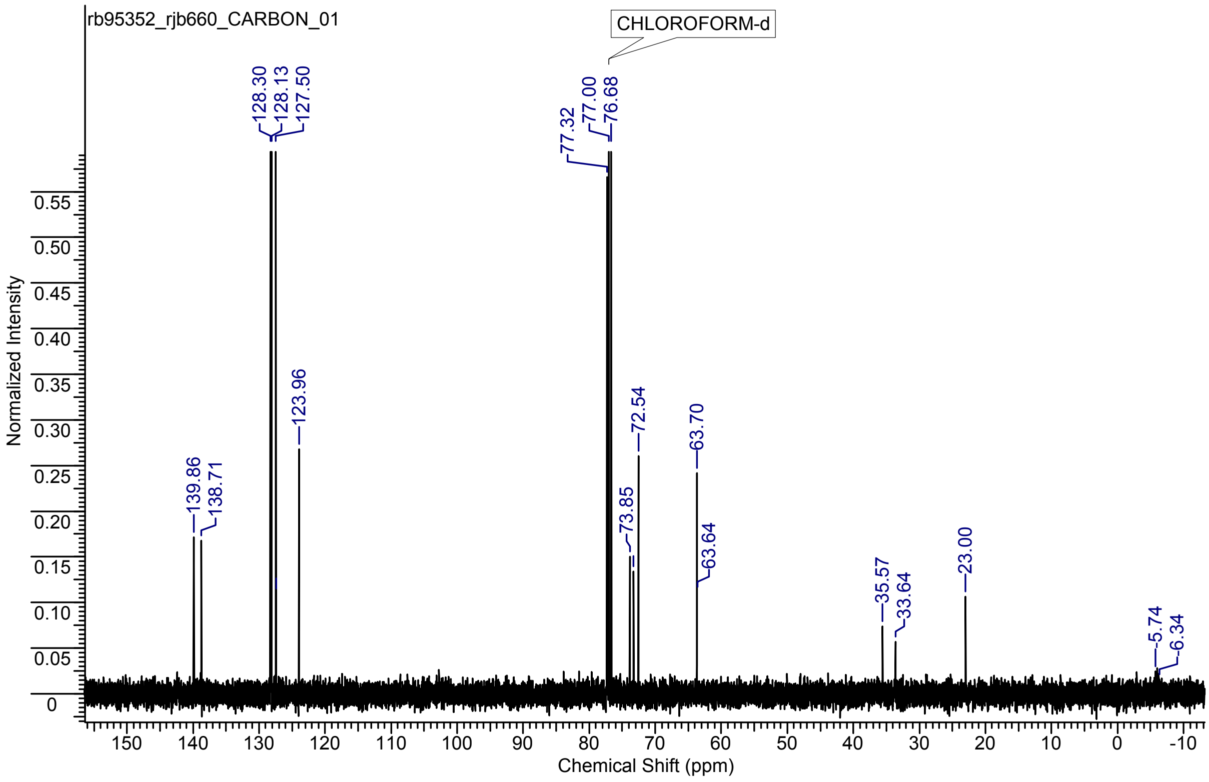

(3*S*,5*R*)-1,3-*O*-Acetyl-5-(1'benzyloxymethyl)-tetrahydropyran 34

^{1}H NMR ($CDCl_3$, 400 MHz)

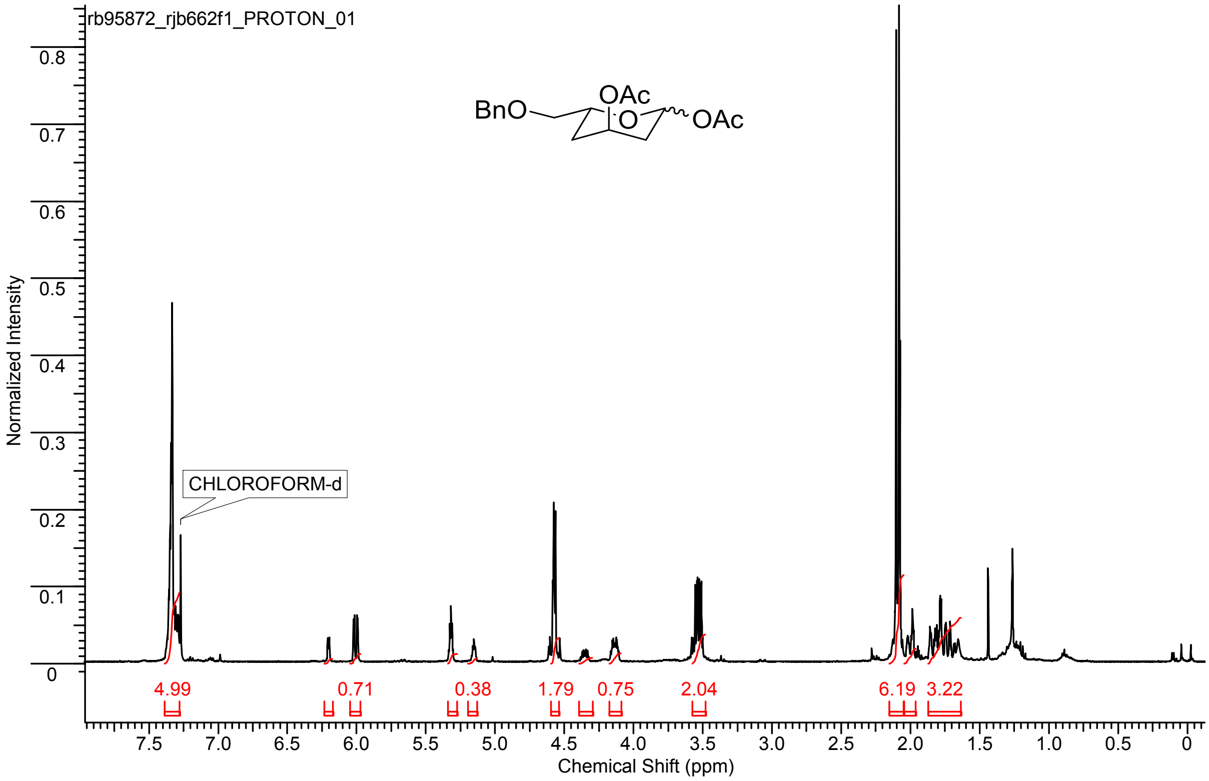

^{13}C NMR ($CDCl_3$, 100 MHz)

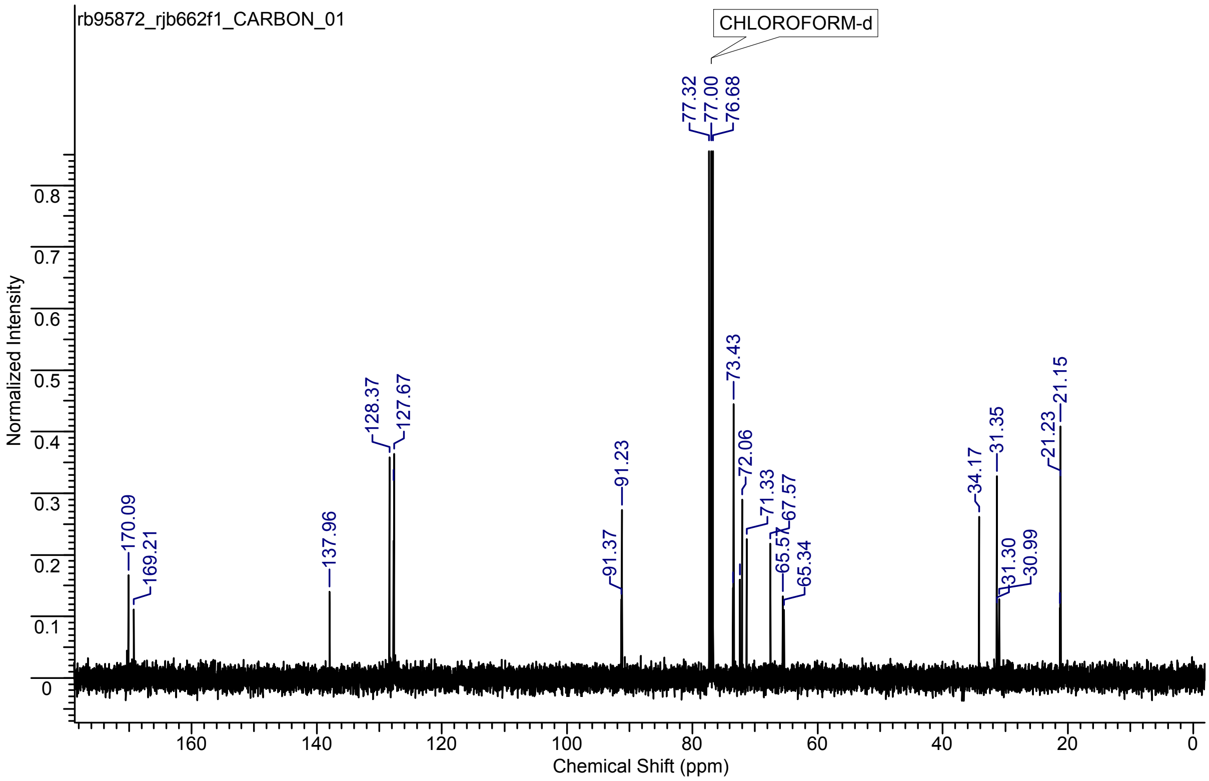

(1*S*,3*R*,4*R*)-1-(Benzyldimethylsilyl)-4-(2'-phenylethyl)-tetrahydrofuran-3-al 36

^{1}H NMR ($CDCl_3$, 400 MHz)

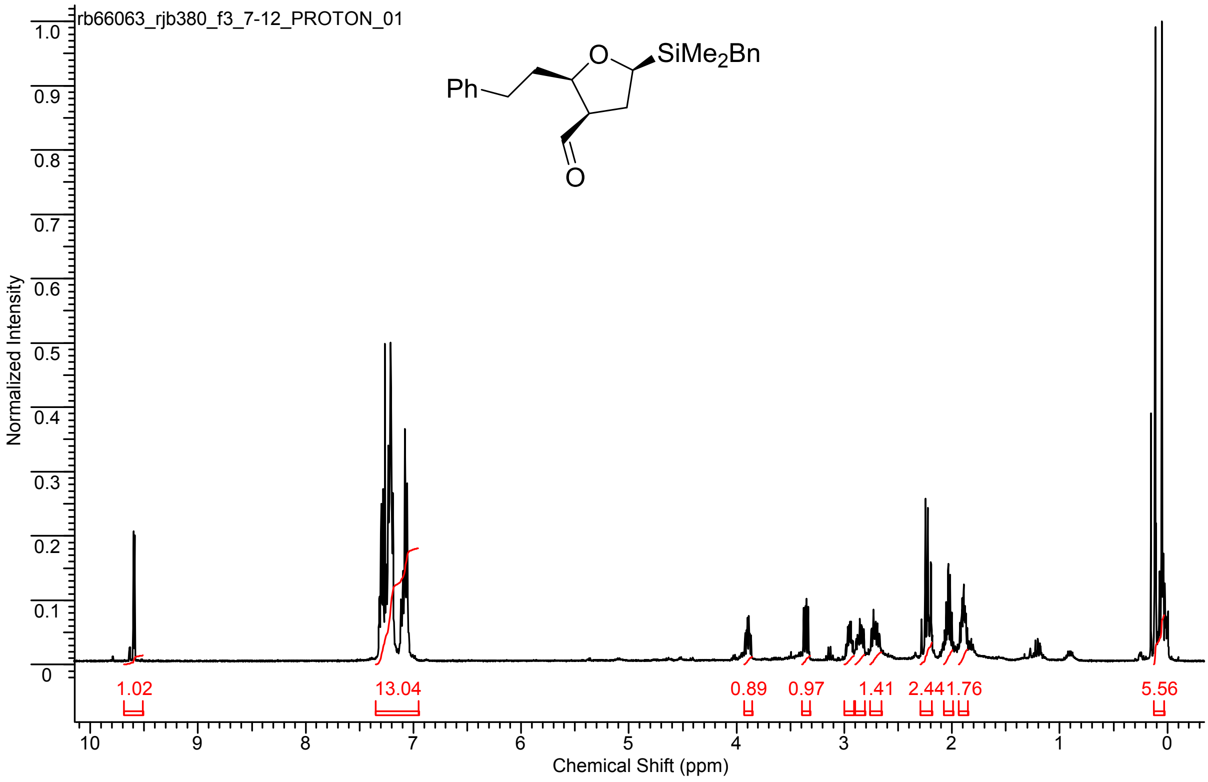

^{13}C NMR ($CDCl_3$, 100 MHz)

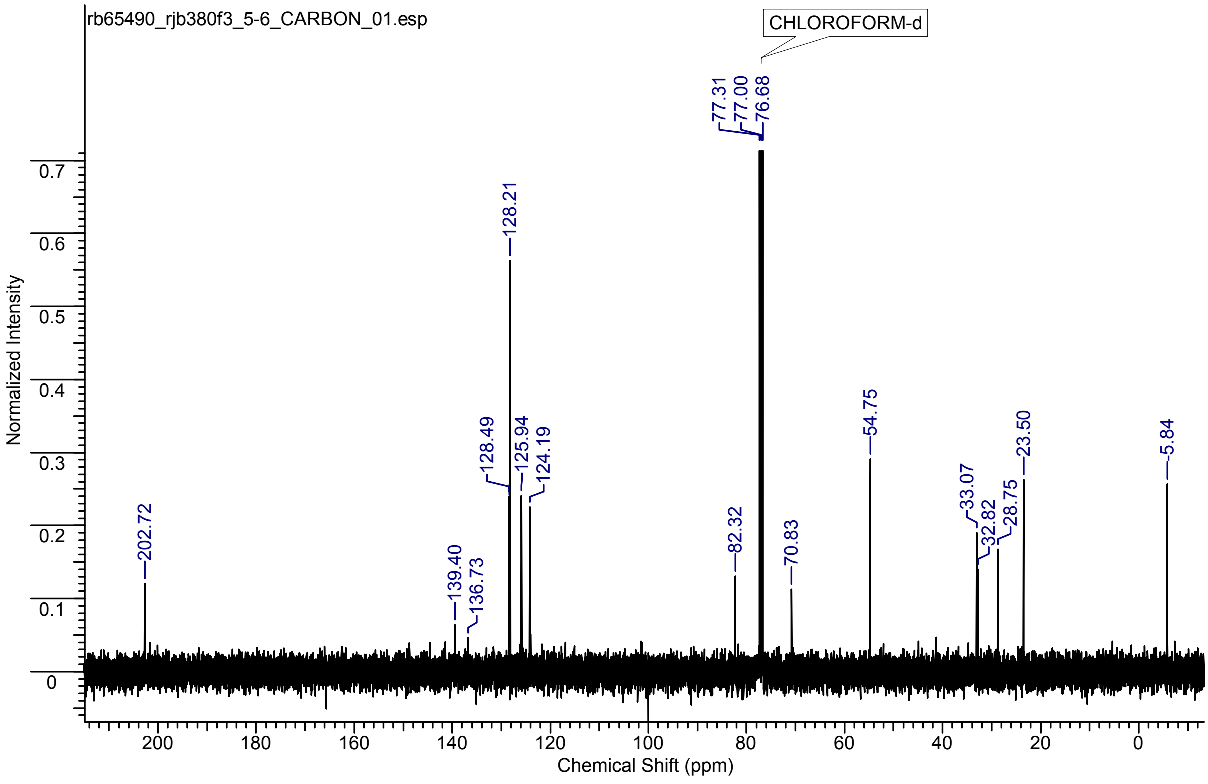

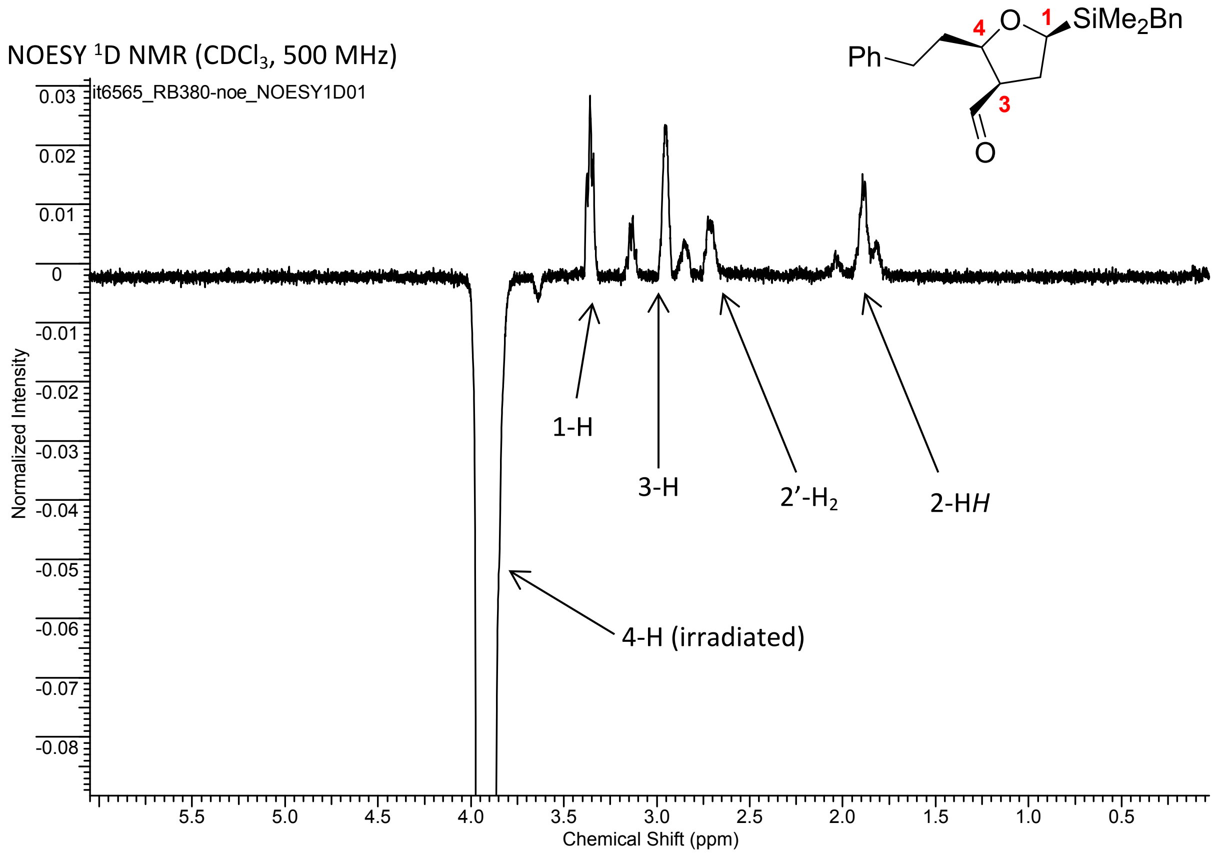
NOESY 1D NMR (CDCl3, 500 MHz)
it6565_RB380-noe_NOESY1D01
Normalized Intensity
Chemical Shift (ppm)
1-H
3-H
2'-H2
2-HH
4-H (irradiated)
Ph
SiMe2Bn
O
4
1
3
O

(2*S*)-1,2-Epoxy-3-*O*-(*N*,*N*-diisopropylcarbamate)-pent-4-en 39

^{1}H NMR ($CDCl_3$, 400 MHz)

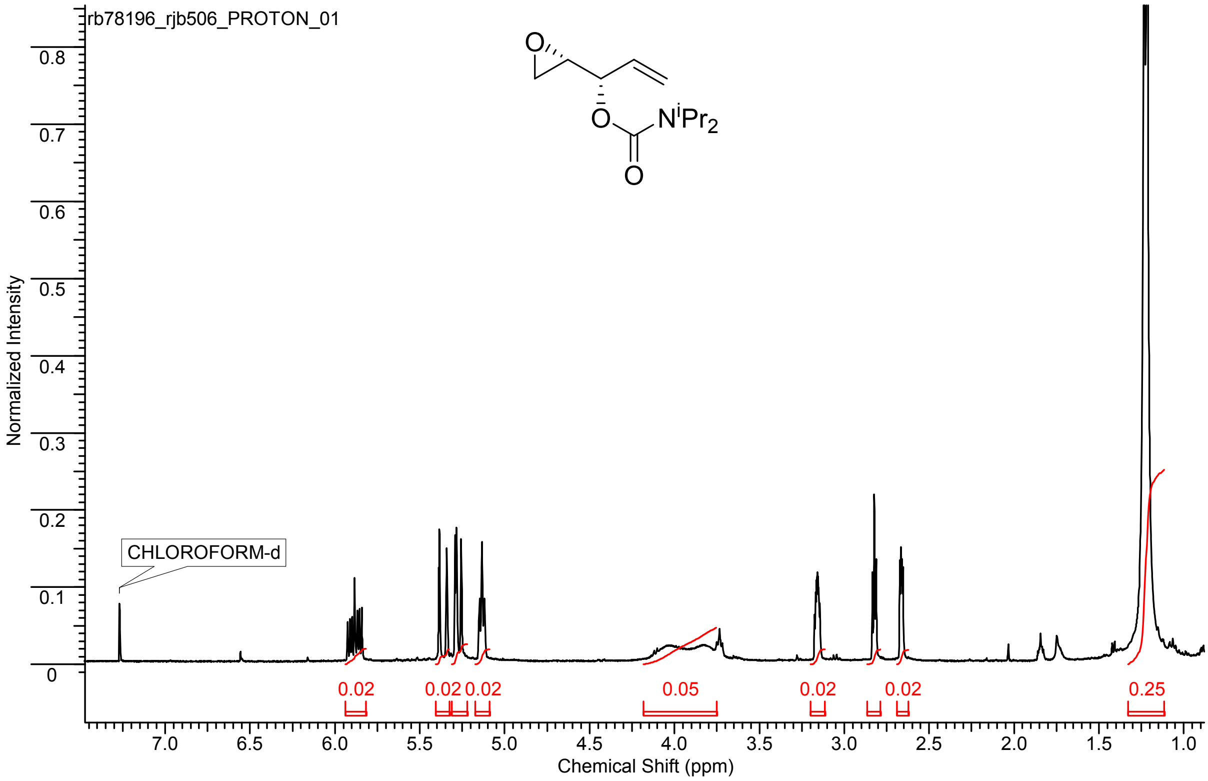

^{13}C NMR ($CDCl_3$, 100 MHz)

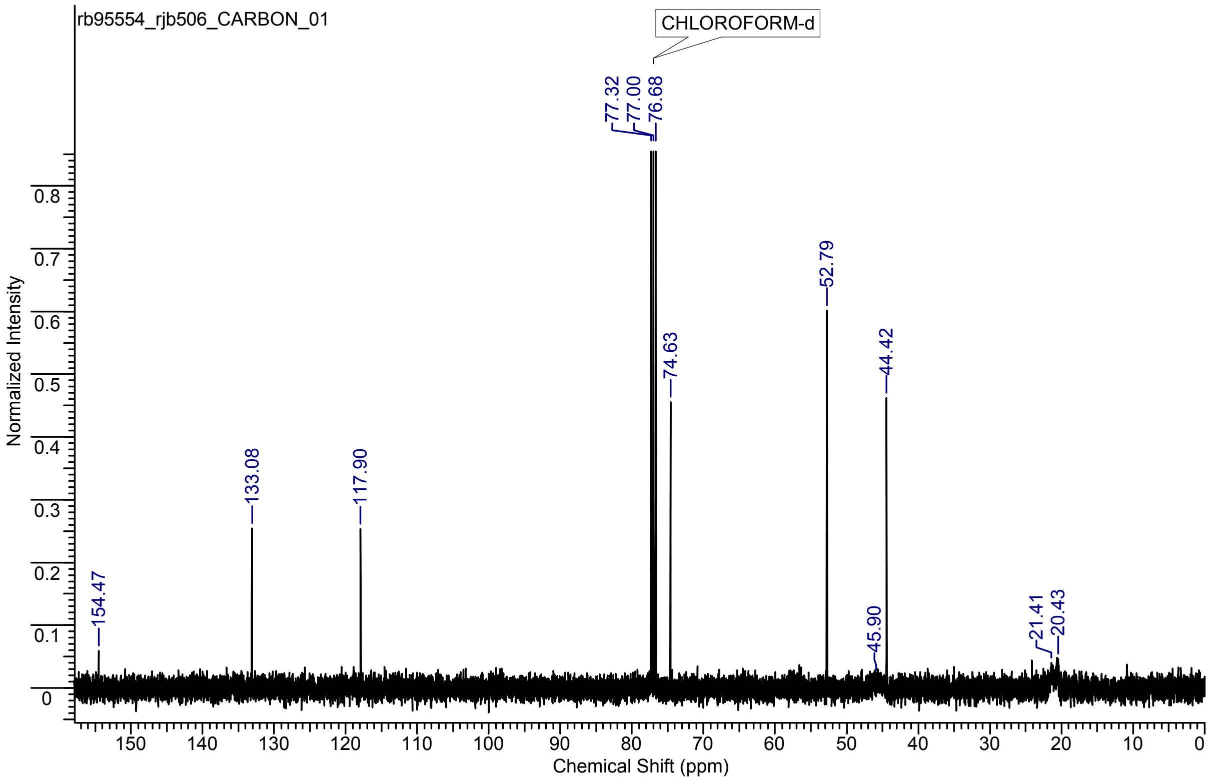

(2*S*,3*S*)-2-Hydroxy-3-*O*-(*N*,*N*-diisopropylcarbamate)-pent-4-en 40

^{1}H NMR ($CDCl_3$, 400 MHz)

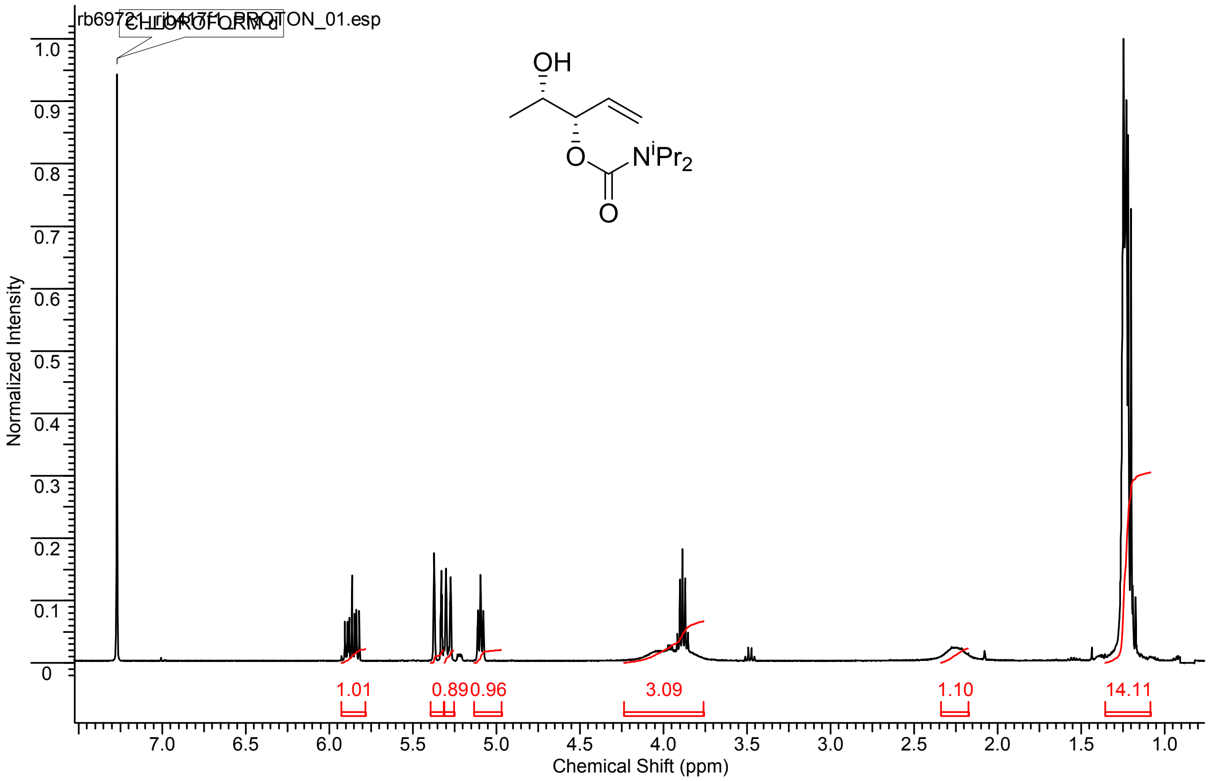

^{13}C NMR ($CDCl_3$, 100 MHz)

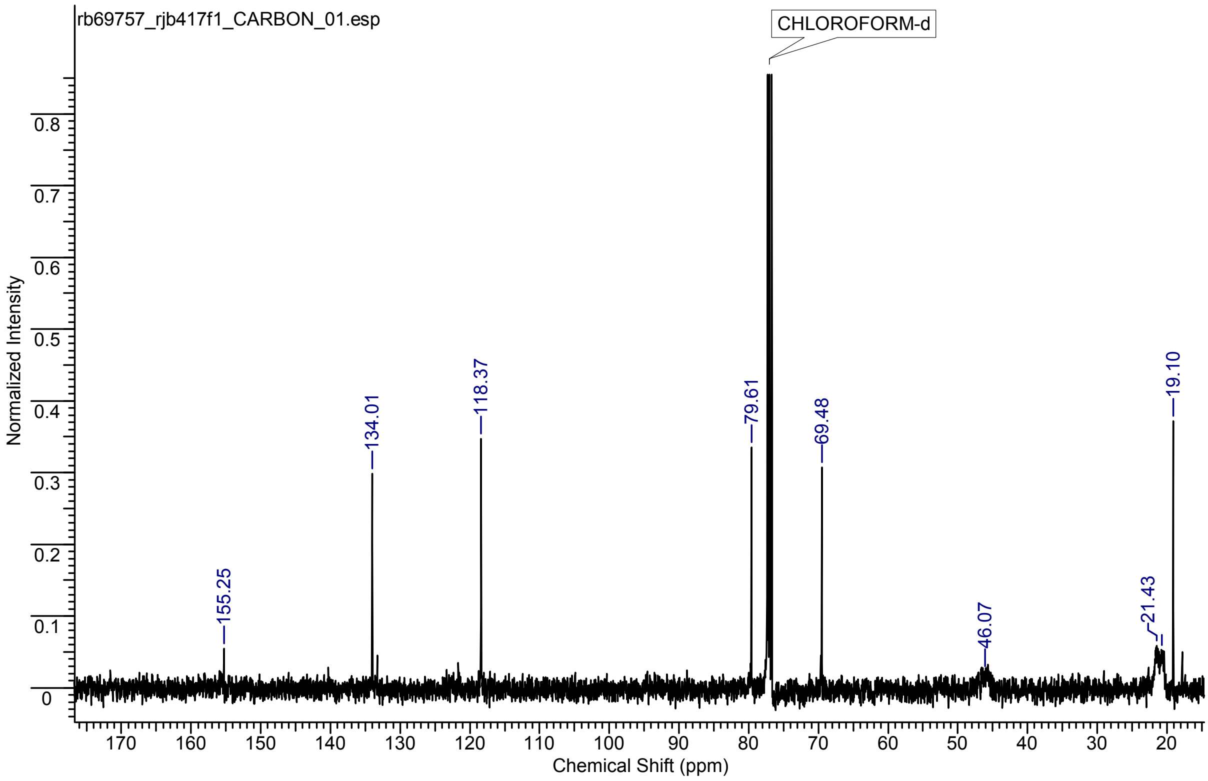

(1*R*,3*S*,4*S*,5*S*)-1-(Benzyldimethylsilyl)-4-*O*-(*N*,*N*-diisopropylcarbamate)-3-hydroxy-5-methyl-tetrahydropyran 41

^{1}H NMR ($CDCl_3$, 400 MHz)

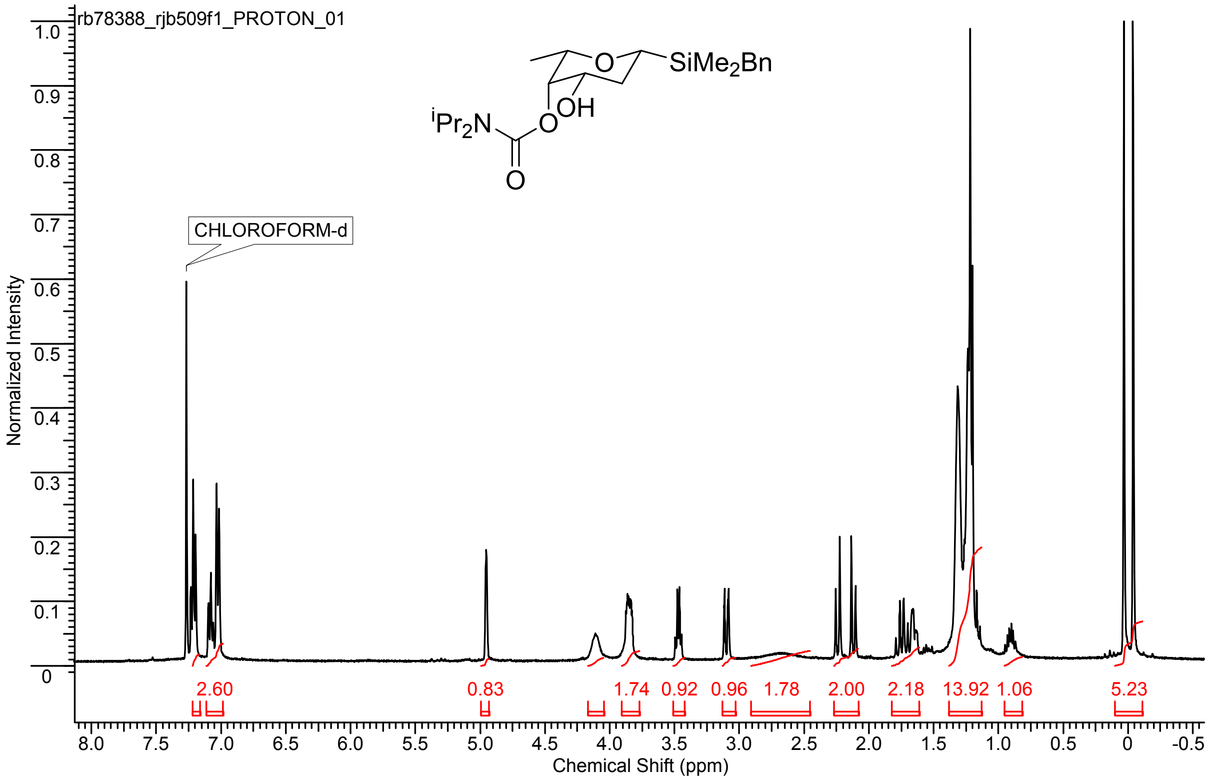

^{13}C NMR ($CDCl_3$, 100 MHz)

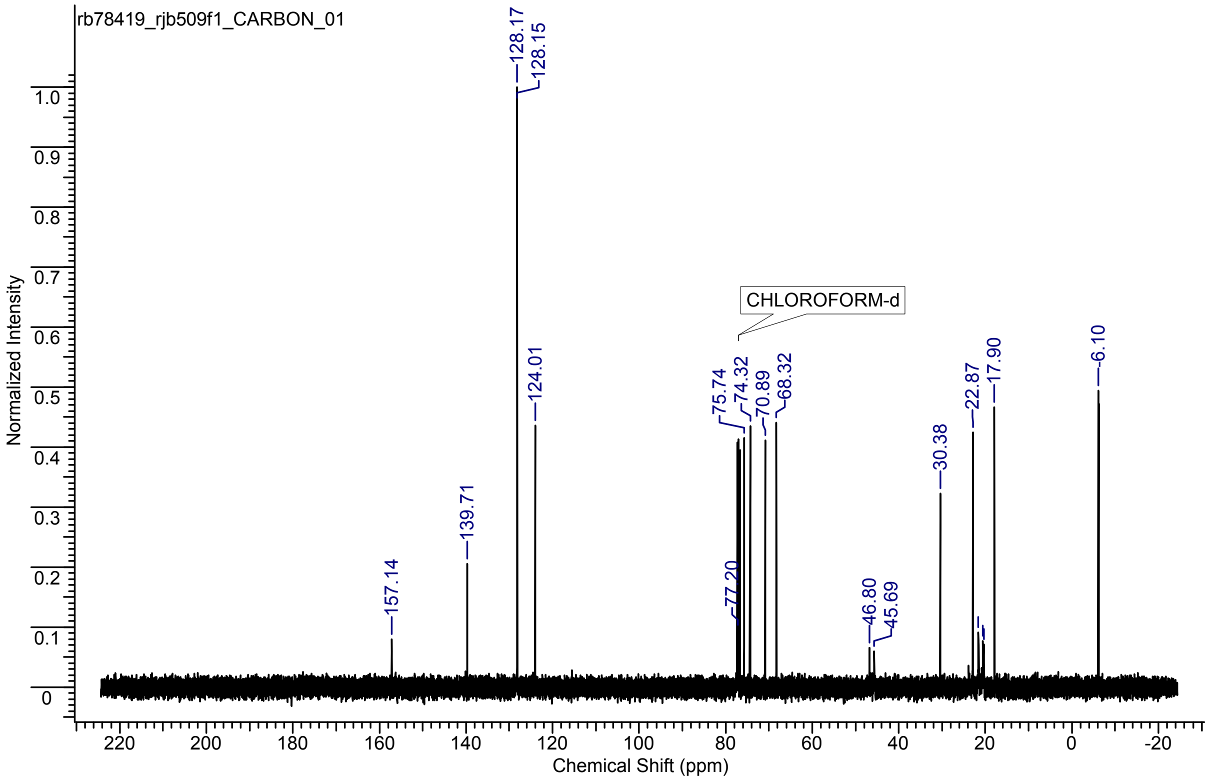

(3*S*,4*S*,5*S*)-1,3-*O*-Acetyl-4-*O*-(*N*,*N*-diisopropylcarbamate)5-methyl-tetrahydropyran 42

^{1}H NMR ($CDCl_3$, 400 MHz)

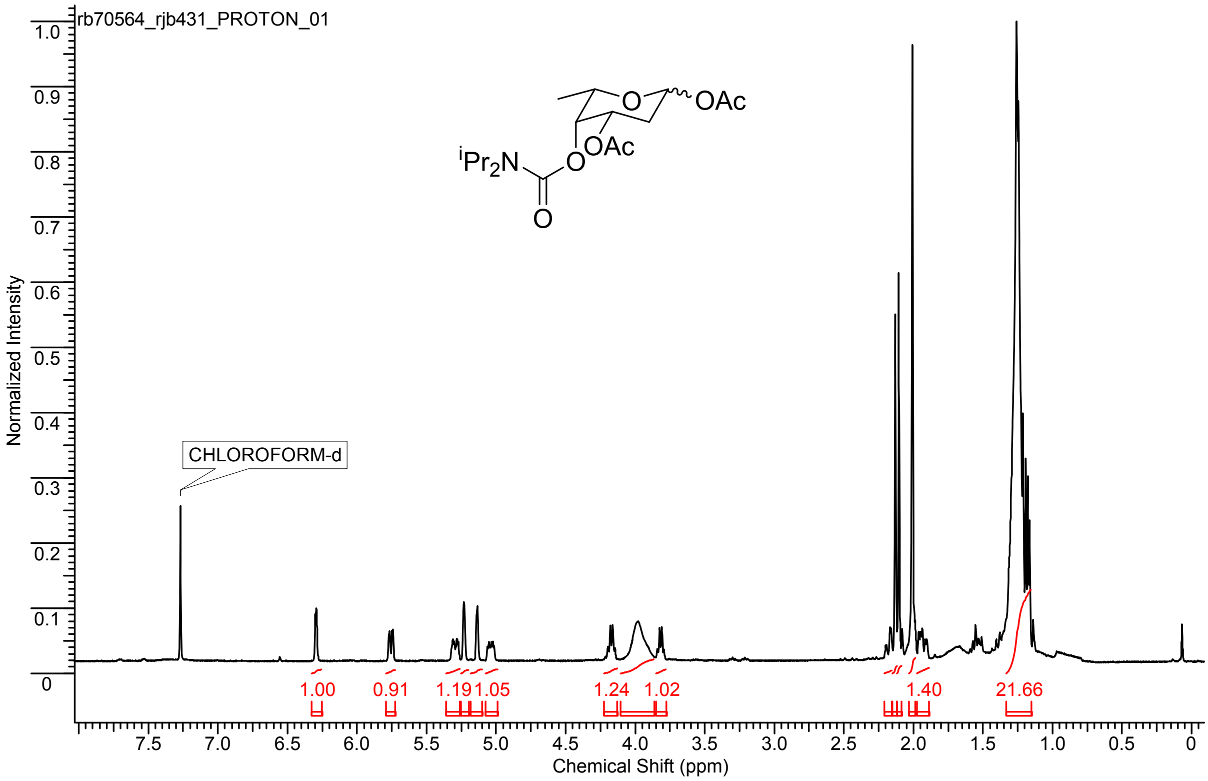

^{13}C NMR ($CDCl_3$, 100 MHz)

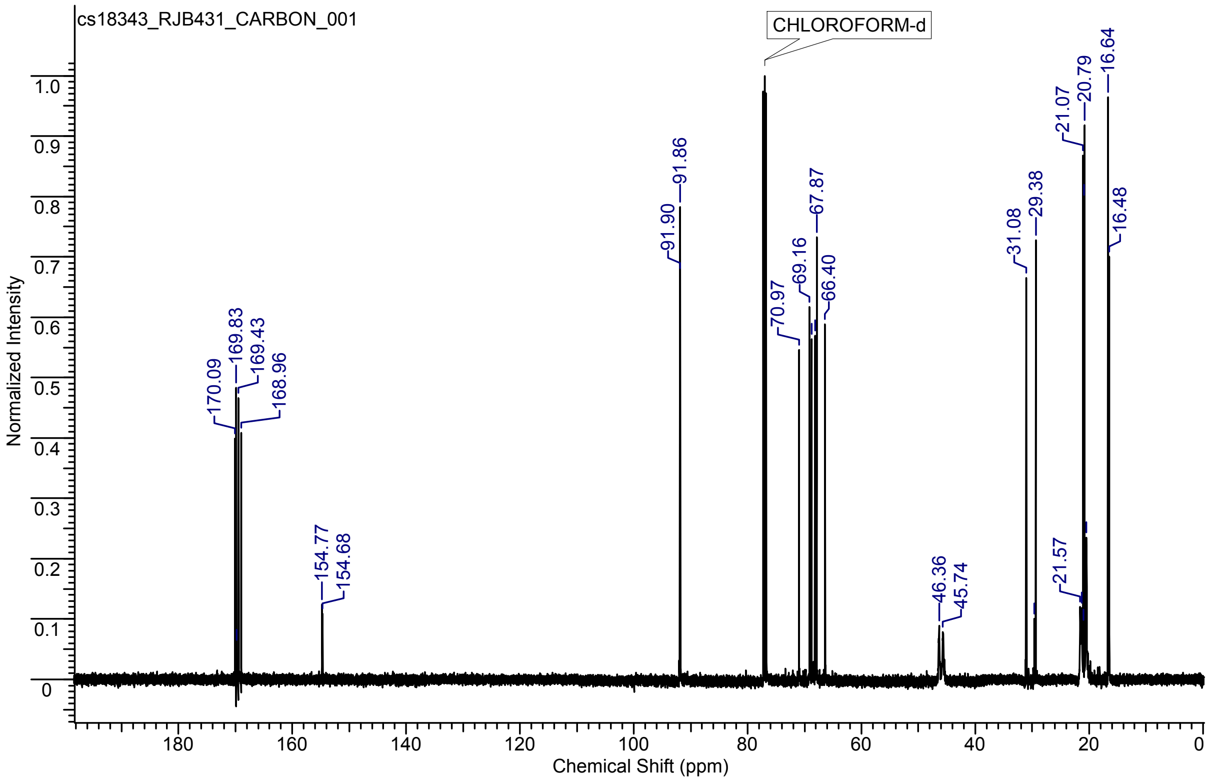

(2*S*,3*R*)-1,2-Epoxy-3-*O*-(*N*,*N*-diisopropylcarbamate)-pent-4-ene 44

^1H NMR ($CDCl_3$, 400 MHz)

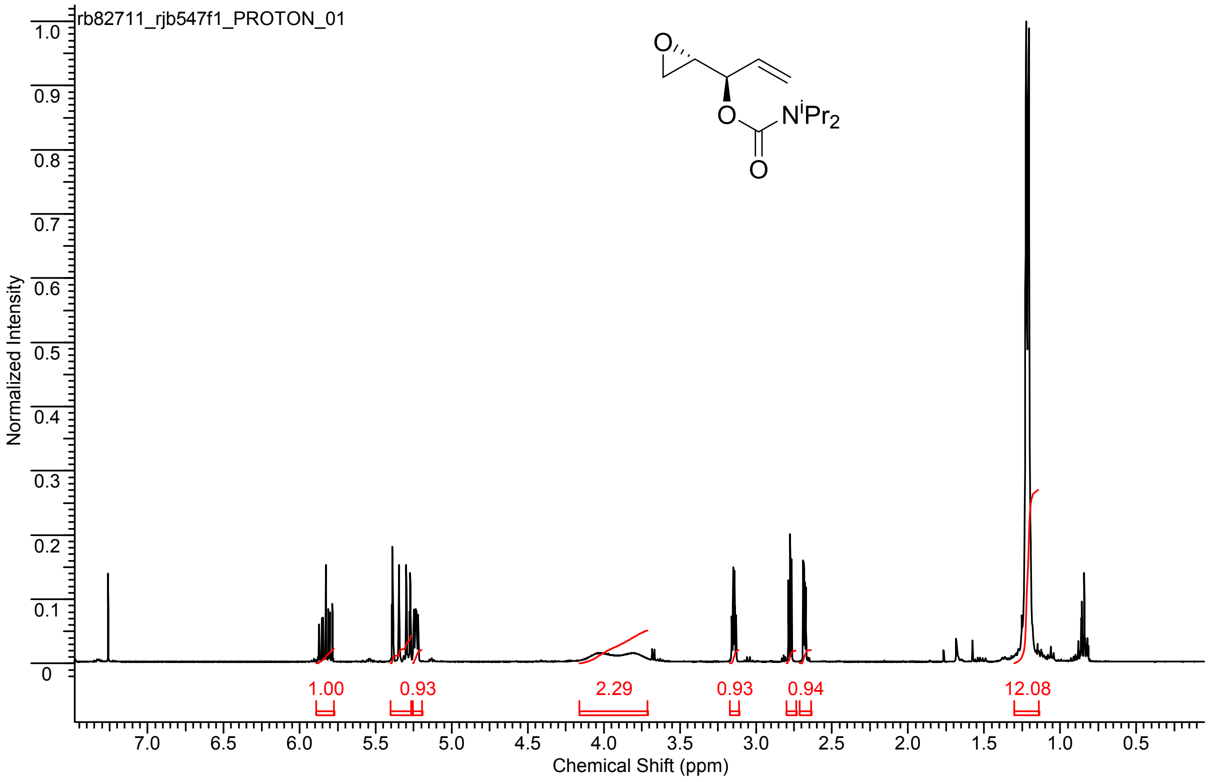

^{13}C NMR ($CDCl_3$, 100 MHz)

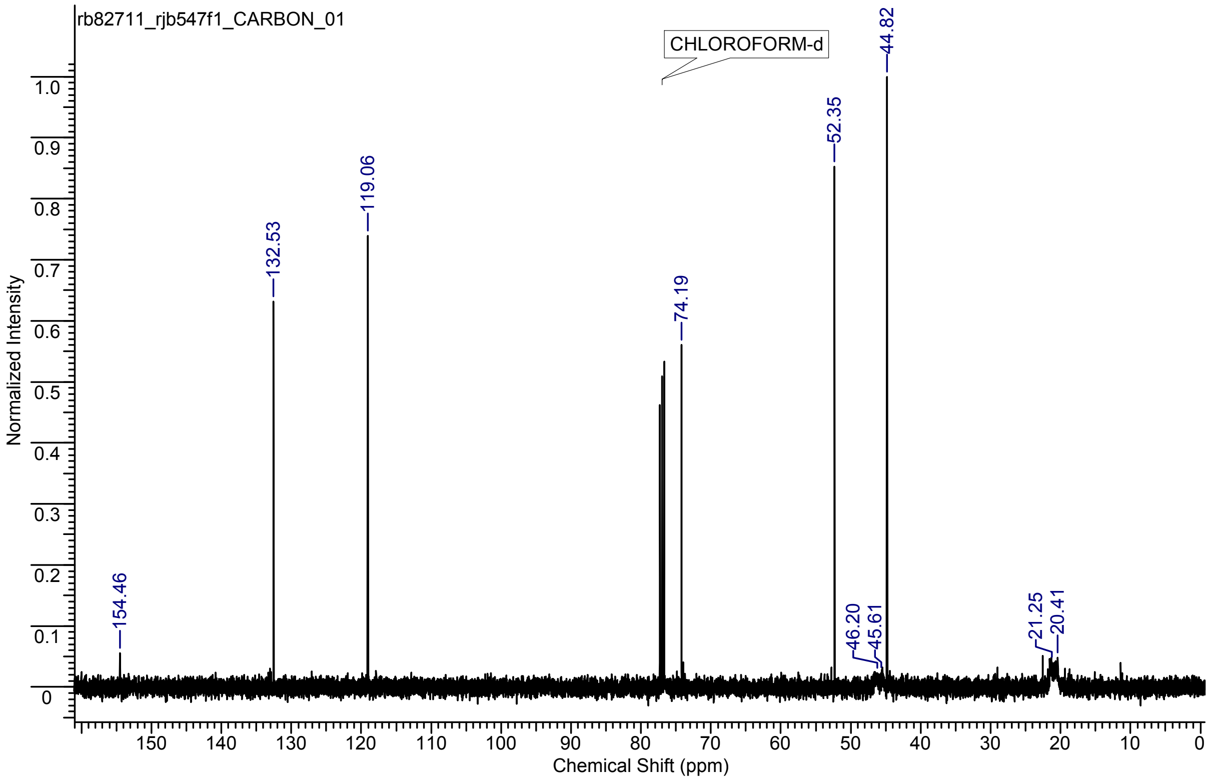

(1*R*,3*S*,4*R*,5*S*)-1-(Benzyl(dimethyl)silane)-3,4-dihydroxy-5-methyl-tetrahydropyran 45

^{1}H NMR ($CDCl_3$, 400 MHz)

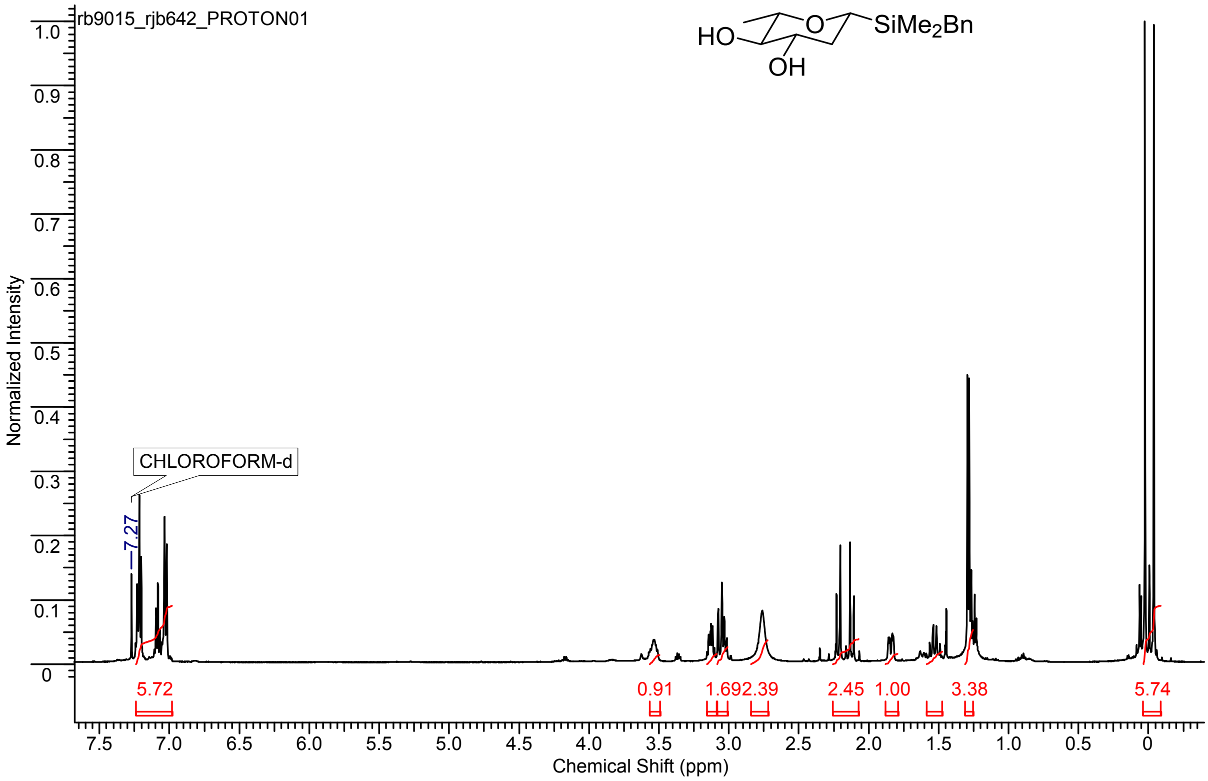

^{13}C NMR ($CDCl_3$, 100 MHz)

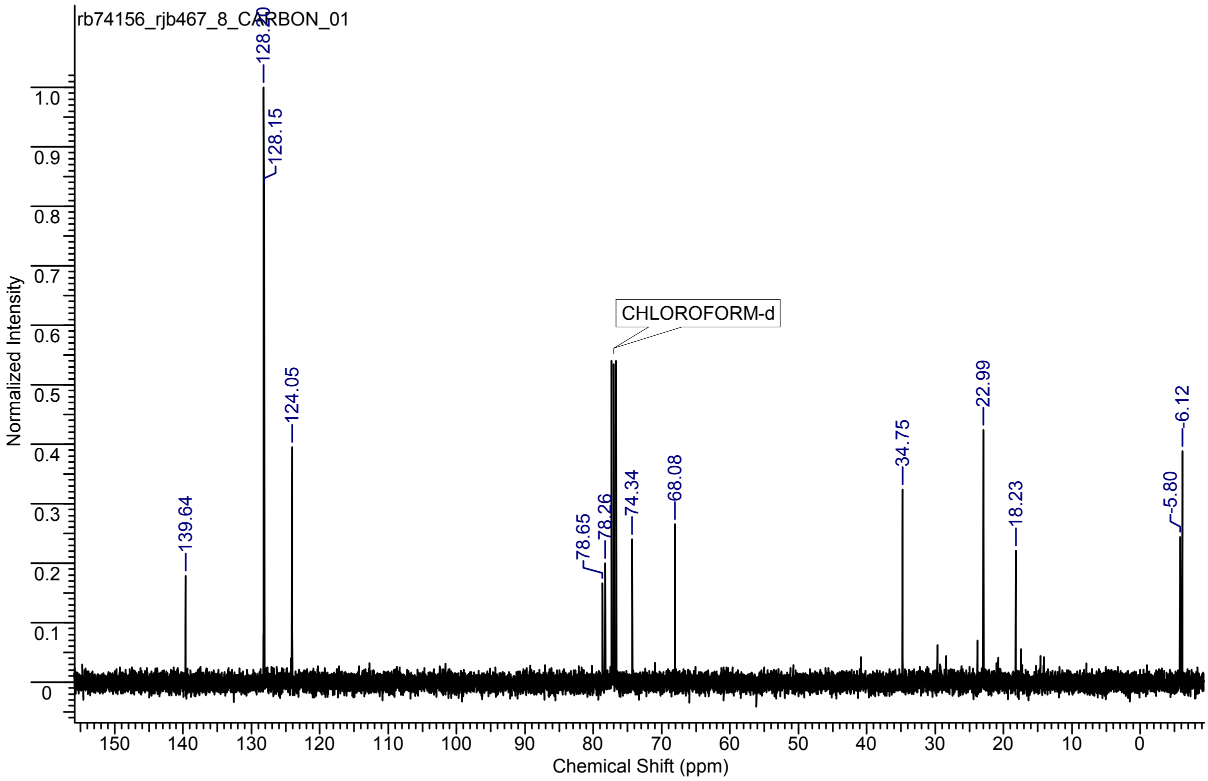

References

(1) Nokami, J.; Ohga, M.; Nakamoto, H.; Matsubara, T.; Hussain, I.; Kataoka, K. *J. Am. Chem. Soc.* **2001**, *123*, 9168.

(2) Díaz, Y.; Bravo, F.; Castillón, S. *J. Org. Chem.* **1999**, *64*, 6508.

(3) Hansen, T. V. *Tetrahedron: Asymmetry* **2002**, *13*, 547.

(4) Dar, A. R.; Aga, M. A.; Kumar, B.; Yousuf, S. K.; Taneja, S. C. *Org. Biomol. Chem.* **2013**, *11*, 6195.

(5) Saikia, B.; Joymati Devi, T.; Barua, N. C. *Org. Biomol. Chem.* **2013**, *11*, 905.

(6) Sabitha, G.; Fatima, N.; Reddy, E. V.; Yadav, J. S. *Tetrahedron Lett.***2008**, *49*, 6087.

(7) Huang, X.-R.; Pan, X.-H.; Lee, G.-H.; Chen, C. *Adv. Synth. Catal.* **2011**, *353*, 1949.

(8) Ito, H.; Nakamura, T.; Taguchi, T.; Hanzawa, Y. *Tetrahedron* **1995**, *51*, 4507.

(9) Romero, A.; Wong, C.-H. *J. Org. Chem.* **2000**, *65*, 8264.

(10) Singh, S.; Guiry, P. J. *J. Org. Chem.* **2009**, *74*, 5758.
